# Proteome-wide analysis of protein abundance and turnover remodelling during oncogenic transformation of human breast epithelial cells

**DOI:** 10.12688/wellcomeopenres.14392.1

**Published:** 2018-05-02

**Authors:** Tony Ly, Aki Endo, Alejandro Brenes, Marek Gierlinski, Vackar Afzal, Andrea Pawellek, Angus I. Lamond

**Affiliations:** 1 Centre for Gene Regulation and Expression, University of Dundee, Dundee, UK; 2 Laboratory for Quantitative Proteomics, University of Dundee, Dundee, UK; 3 Wellcome Centre for Cell Biology, School of Biological Sciences, University of Edinburgh, Edinburgh, UK; 4 Cell Biology Center, Institute of Innovative Research, Tokyo Institute of Technology, Tokyo, Japan

**Keywords:** Cancer, Time-lapse Proteomics, Src Kinase, Protein half-life, Biomarker, Serine protease inhibitor (Serpin), Polycomb Repressive Complex (PRC), Epigenetic

## Abstract

**Background**: Viral oncogenes and mutated proto-oncogenes are potent drivers of cancer malignancy. Downstream of the oncogenic trigger are alterations in protein properties that give rise to cellular transformation and the acquisition of malignant cellular phenotypes. Developments in mass spectrometry enable large-scale, multidimensional characterisation of proteomes. Such techniques could provide an unprecedented, unbiased view of how oncogene activation remodels a human cell proteome.

**Methods**: Using quantitative MS-based proteomics and cellular assays, we analysed how transformation induced by activating v-Src kinase remodels the proteome and cellular phenotypes of breast epithelial (MCF10A) cells. SILAC MS was used to comprehensively characterise the MCF10A proteome and to measure v-Src-induced changes in protein abundance across seven time-points (1-72 hrs). We used pulse-SILAC MS ( Boisvert *et al*., 2012), to compare protein synthesis and turnover in control and transformed cells. Follow-on experiments employed a combination of cellular and functional assays to characterise the roles of selected Src-responsive proteins.

**Results**: Src-induced transformation changed the expression and/or turnover levels of ~3% of proteins, affecting ~1.5% of the total protein molecules in the cell. Transformation increased the average rate of proteome turnover and disrupted protein homeostasis. We identify distinct classes of protein kinetics in response to Src activation. We demonstrate that members of the polycomb repressive complex 1 (PRC1) are important regulators of invasion and migration in MCF10A cells. Many Src-regulated proteins are present in low abundance and some are regulated post-transcriptionally. The signature of Src-responsive proteins is highly predictive of poor patient survival across multiple cancer types. Open access to search and interactively explore all these proteomic data is provided via the EPD database ( www.peptracker.com/epd).

**Conclusions**: We present the first comprehensive analysis measuring how protein expression and protein turnover is affected by cell transformation, providing a detailed picture at the protein level of the consequences of activation of an oncogene.

## Introduction

**Figure 1.**
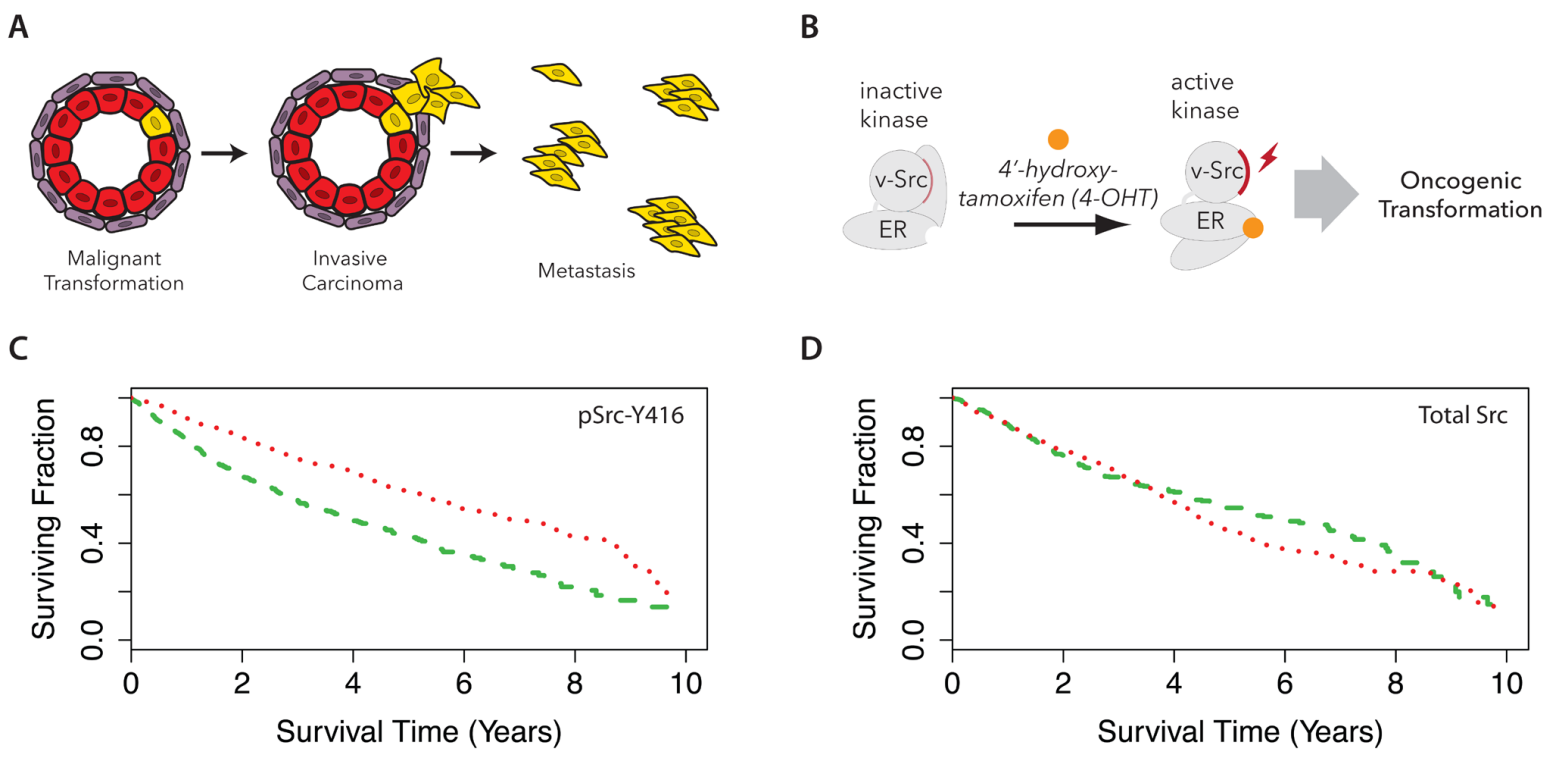
Active Src kinase is a predictor of poor clinical outcome. Cartoon schematics illustrating the development of metastatic phenotypes ( **A**), and the Src-ER model system for oncogenesis ( **B**). ( **C**) The Cancer Genome Atlas patients were stratified into three cohorts based on reverse phase protein array intensities for pSrc-Y416. Kaplan-Maier curves for patients showing the top (green dashed line) and bottom (red dotted line) third signal are plotted. Log rank test p < 1 × 10 ^-10^. ( **D**) A similar survival analysis as ( **C**) for total Src protein level signal.

Cancer malignancies have in common the development of cellular phenotypes that alter the normal behaviour of the respective terminally differentiated cell types. Advanced forms of malignancy that are associated with poor clinical outcomes, including high-grade breast and oesophageal tumours, are characterized by tumour invasion into the surrounding stroma (illustrated in Figure 1A) and the development of metastases at sites distal to the initial tumour. Specific phenotypes, or ‘hallmarks’ ( Hanahan & Weinberg, 2011), are associated with late stage development of cancer and strongly linked with poor clinical outcomes for patients. These include increased motility and invasion, migration and immune cell evasion phenotypes, which are not active in the healthy differentiated cells. This is reflected in corresponding changes in patterns of gene expression in the transformed cells, leading to changes in the ‘properties’, e.g. including the abundance, post-translational modification, half-life and/or activity, of specific subsets of proteins that mediate the metastatic phenotypes. Such profound changes in gene expression can be triggered endogenously by the mutation of proto-oncogenes and tumour suppressors, and/or exogenously, e.g. by viral expression of oncogenes, such as v-Src ( Rous, 1910).

The v-Src gene has played multiple paradigmatic roles in advancing our understanding of cell biology and disease mechanisms ( Yeatman, 2004). v-Src was first discovered over a century ago as a viral oncogene that triggers cellular transformation and cancer malignancy in chicken cells ( Rous, 1910). Decades later, the study of v-Src kinase activity resulted in the discovery of a new form of signalling through protein tyrosine phosphorylation ( Eckhart *et al.*, 1979).

c-Src is the human homologue of v-Src and is one of several Src family kinases (SFKs) encoded in the human genome ( Thomas & Brugge, 1997). Like v-Src, SFKs are potent protein tyrosine kinases. Human SFKs regulate diverse functions in cells, including T-cell activation ( Seddon & Zamoyska, 2002), cell motility ( Hsia *et al.*, 2003), and cell focal adhesions ( Jones *et al.*, 2000). In common with many kinases, the switching of Src from an inactive to active form is stimulated by its phosphorylation in the activation loop, i.e. on tyrosine Y416. c-Src is also regulated by reversible phosphorylation on a C-terminal tyrosine residue (Y527) ( Thomas *et al.*, 1991), which, when phosphorylated, auto-inhibits kinase activity. v-Src lacks this Y527 residue, and is therefore thought to be constitutively active. Consistent with these observations, unlike c-Src, overexpression of v-Src alone is sufficient to drive tumourigenicity in human cells.

To understand better the downstream phenotypic consequences of Src kinase-mediated cell transformation, we have undertaken a detailed characterization of the molecular mechanisms triggered by Src, using a comprehensive, unbiased proteomic approach ( Bekker-Jensen *et al.*, 2017; Geiger *et al.*, 2012; Ly *et al.*, 2014; Mann *et al.*, 2013). Our hypothesis is that the resulting protein-level data may provide important new insights that reveal key pathways, downstream of SFK activity, which drive the changes in cell phenotype associated with oncogenic transformation.

To perform these analyses, we have taken advantage of the well characterized MCF10A Src-ER (Src-ER) human epithelial cell model for oncogenic transformation resulting from activation of v-Src kinase activity ( Hirsch *et al.*, 2010; Iliopoulos *et al.*, 2009). These cells constitutively express a fusion protein between v-Src and the oestrogen-responsive, repressive domain of oestrogen receptor. Under basal conditions, the fusion protein is expressed, but the cells are not transformed, because the Src-ER fusion shows only low levels of kinase activity. However, when these cells are exposed to the steroid hormone, 4-hydroxytamoxifen (4-OHT), there is a resulting elevated burst of v-Src tyrosine kinase activity that triggers events causing the cells to undergo phenotypic transformation within 48–72 hrs ( Figure 1B). As a result, the cells lose contact-inhibition, show increased motility, display heterogeneous morphologies and become tumourigenic in mouse models ( Iliopoulos *et al.*, 2009).

We recently used the MCF10A Src-ER model to study how cell transformation affects specifically the chromatin proteome ( Endo *et al.*, 2017). In this current study, we have significantly expanded both the scope and scale of our unbiased proteomic characterization of this cell model. Using a quantitative, mass spectrometry (MS)-based approach, we have characterized in depth the global proteome of untransformed, human epithelial Src-ER cells and also measured the proteome at seven time points, spanning 1 to 72 hr, after activation of v-Src kinase. We also performed a global, MS-based analysis of protein synthesis and turnover, both in untransformed cells and in the same cells following Src-induced transformation.

Open access to all of the resulting data, representing the most comprehensive, quantitative description to date of the detailed changes in protein abundance and protein dynamics accompanying oncogenic transformation, is provided via the Encyclopedia of Proteome Dynamics (EPD) ( Brenes *et al.*, 2017), a searchable online database.

## Results

With the aim of identifying a relevant cellular model in which to characterize in depth how oncogenic transformation remodels the cell proteome, we first performed a meta-analysis of existing data sets provided by The Cancer Genome Atlas (TCGA) ( Cancer Genome Atlas Research Network *et al.*, 2013) to identify proteins correlated with poor clinical outcome. Kaplan-Meier (KM) survival curves were generated from each antigen in the TCGA reverse phase protein array (RPPA) data set ( Akbani *et al.*, 2014). We compared how variation in the expression levels of each of these antigens correlated with patient survival ( Supplementary Table 1).

One of the most striking effects seen in this analysis was a significant decrease in median survival time of approximately 4 years observed for patients showing the highest expression levels of Src-pY416 ( Figure 1C), a marker for SFK activity (log rank test p < 0.001). In contrast, no significant difference in median patient survival time was observed for stratification based on total levels of Src protein ( Figure 1D).

These data are consistent with previous reports ( Elsberger *et al.*, 2010) that it is the levels of Src kinase activity, not total Src protein expression levels, that correlate with poor patient outcome across multiple cancer types in the clinic. Based on these data, we therefore focused our quantitative proteomic analysis on characterising a cellular transformation model driven specifically by activation of Src kinase activity in human epithelial cells ( Iliopoulos *et al.*, 2009).

### Proteomic analysis of v-Src activation: overview of experimental design

**Figure 2.**
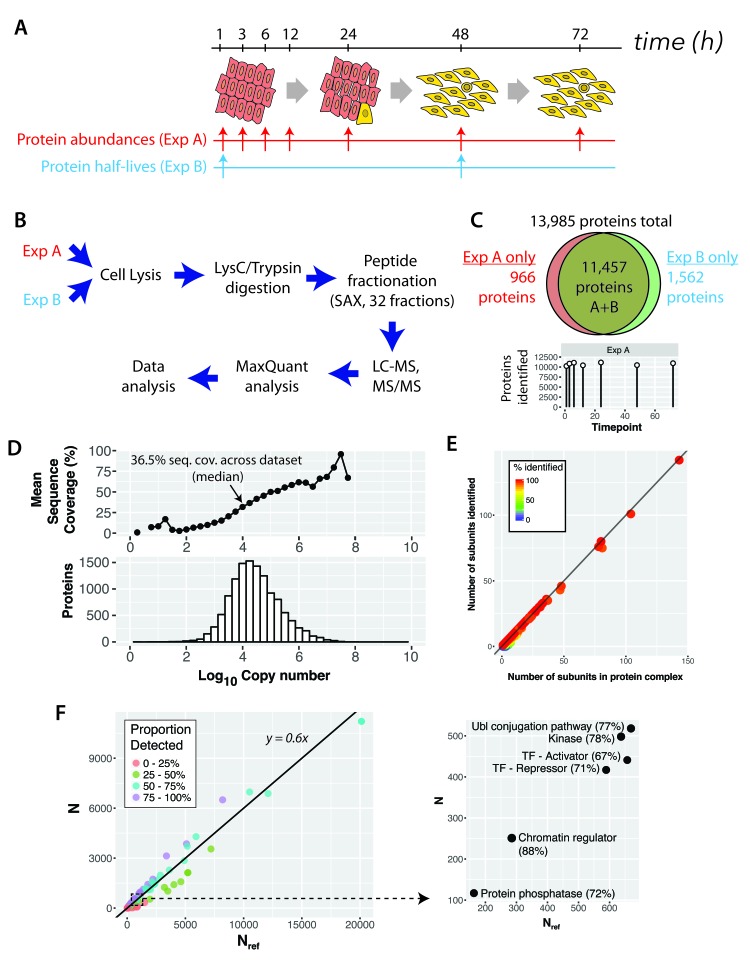
A comprehensive proteomic analysis of basal and Src-transformed human epithelial cells. ( **A**) Experimental design to characterise the changes to protein abundances (“Exp A”) and half-lives (“Exp B”) proteome-wide in a cellular model of oncogenic transformation (MCF10A Src-ER). ( **B**) Sample preparation and analysis workflow, including extensive peptide pre-fractionation prior to liquid chromatography-tandem mass spectrometry (LC-MS/MS). ( **C**) Venn diagram showing the number of proteins (protein groups) identified in each and in both experiments. The bottom panel shows the number of proteins identified in each time point of Experiment A. ( **D**) The distribution of protein abundances (bottom panel) spans 8 orders of magnitude. An analysis of mean sequence coverage in protein abundance bins (top panel). The average sequence coverage (%, top panel) is 36.5% across the entire data set, but approaches 100% for the bins containing the most abundant proteins. ( **E**) A plot of number of CORUM (Comprehensive Resource of mammalian Protein Complexes) subunits experimentally detected versus number of subunits listed in CORUM. Each point represents a different CORUM complex. The line approximates to y = x, a situation where all CORUM complexes are completely detected. ( **F**) An analysis of UniProt keywords comparing the number detected versus the total number in the reference proteome (UniProt) for all keywords (left). The line is a best-fit regression. A zoomed-in section is also shown (right) to highlight selected keywords.

To assess the effect of Src-induced cell transformation at the protein level, we designed a two-part experimental strategy to analyse changes in both protein abundance (Experiment (Exp) A) and protein turnover (Exp B), as summarised in Figure 2. Exp A involved characterizing in depth the proteome of human MCF10A Src-ER epithelial cells and then systematically analysing global proteome changes in these cells across seven time points, following activation of v-Src kinase activity i.e., 1 hr, 3 hr, 6 hr, 12 hr, 24 hr, 48 hr and 72 hr ( Figure 2A, Exp A). Exp B involved measuring protein half-lives in both the basal, untransformed cell state and in the transformed state, i.e. comparing cells -/+ 48 hours of v-Src activation, using our previously described pulse SILAC labeling strategy ( Boisvert *et al.*, 2012) (see Methods and Supplementary File 1). The overall experimental design is illustrated in Figure 2B. All experiments, in both Exp A and Exp B, were performed in biological triplicate, with each replicate harvested on a different day.

Briefly, the proteomic workflow involved SILAC labelling of MCF10A Src-ER cells, either comparing control- versus OHT-treated cells (Exp A) or pulse-SILAC to measure protein turnover (Exp B). In both cases, SILAC labelled cells were then mixed in a 1:1 cell number ratio, lysed, and the extract digested with lysyl endopeptidase C (LysC) and trypsin. The resulting peptides were fractionated using hydrophilic Strong Anion Exchange (hSAX) chromatography into 32 fractions and each fraction was analysed on a Q-Exactive Plus Orbitrap mass spectrometer (MS) instrument, using 2-hr nano LC gradients.

The proteomic analyses in this study generated >2,000 raw MS files, all of which are freely available via the ProteomeXchange PRIDE repository (PRIDE accession PXD009270). In total, >33 million MS1 spectra and >95 million MS2 spectra were acquired. Analysis of the spectra (see Methods for details) resulted in >19 million peptide spectrum matches (PSMs), which identified >350,000 unique peptides (including post translationally modified peptides), with >200,000 corresponding to unique, unmodified peptide sequences. These peptides were mapped to ~13,900 protein groups, with a median protein sequence coverage of ~36% per protein ( Figure 2C). For further discussion of the numbers of proteins and isoforms expressed and methods for estimating integrated protein false discovery rates (FDR), see Methods.

Most of the protein groups were identified in both the time course (Exp A) and protein turnover (Exp B) experiments (cf. Figure 2A), with 966 and 1,562 protein groups exclusively detected in Exp A and Exp B, respectively ( Figure 2C). We identified >10,000 protein groups at each of the seven time-points analysed after v-Src activation ( Figure 2C).

### Overview of the epithelial proteome in untransformed cells

First, we characterised the proteome of untransformed epithelial cells with respect to protein expression and protein turnover. The protein groups identified by MS analysis represent ~55% of the reference SwissProt total human proteome (see Methods). This level of coverage is comparable to recent deep proteome analyses reported for transformed human cell lines, e.g. ( Bekker-Jensen *et al.*, 2017). Collectively, these findings suggest that a differentiated human cell may typically express at the protein level up to ~70% of the protein coding genes in the human genome.

### Depth of epithelial proteome coverage

To investigate further how comprehensively our present data set describes the human epithelial cell proteome, we evaluated the depth of proteome coverage using several approaches. First, we compared how protein sequence coverage was affected by protein copy number (see below for discussion of copy number estimations). While the median sequence coverage across the entire data set was ~36.5% per protein, this rose to >95% for many of the most abundant proteins ( Figure 2D). This is consistent with the data set providing a detailed picture of the proteins expressed in these cells.

Second, we determined the subunit coverage across the core human protein interactome, i.e., complexes curated by CORUM ( Ruepp *et al.*, 2008). Figure 2E shows that the subunit coverage for proteins expressed in this cell line is near 100% for almost all complexes. This indicates that our data set captures a broad spectrum of expressed protein complexes, spanning highly multimeric complexes with >100 subunits, to smaller complexes with <10 subunits.

Third, we addressed what range of known biological functions and protein classes were included amongst the proteins expressed in this epithelial cell line. To do this, we compared the numbers of proteins detected with each UniProt keyword annotation between our data set and the entire reference SwissProt proteome ( Figure 2F). The relationship between our empirically determined data set and the SwissProt reference data set is well described by linear regression analysis (r ^2^ = 0.97), with an average UniProt keyword annotation coverage of ~60%. For comparison, a recent comprehensive study of the HeLa cell proteome reported an average UniProt keyword coverage of ~66% ( Bekker-Jensen *et al.*, 2017). This again suggests that our data set provides a comprehensive view of the proteins expressed in this cell line.

Further analysis of the data set ( Supplementary Table 2), shows essentially complete detection (~100%) of proteins annotated with the UniProt keywords describing most core cell and metabolic functions (covering >100 Keywords). This is consistent with comprehensive detection of most proteins expressed from so-called ‘housekeeping’ genes. In contrast, we detect expression of ~50% of the proteins annotated with ~500 further UniProt Keywords, which describe a broader range of protein classes and cell type-specific expression patterns.

**Figure 3.**
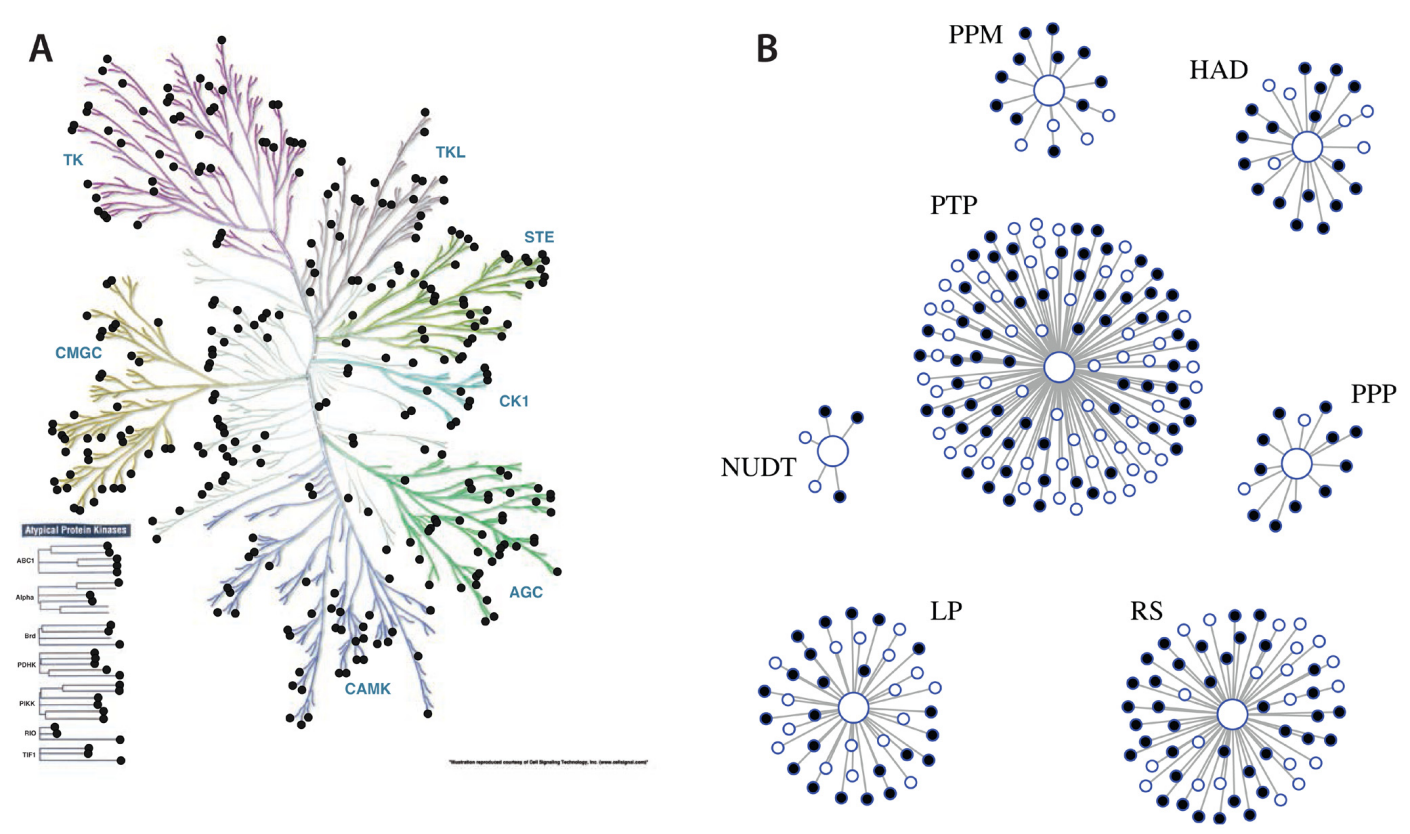
Coverage of the human kinome and phosphatome. Illustrations of ( **A**) the kinome and ( **B**) the phosphatome, with the proteins detected in human epithelial MCF10A cells indicated by solid points.

Coverage of the epithelial cell proteome is further illustrated in Figure 2F, (right panel), which shows selected protein classes, namely kinases, phosphatases, proteins involved in protein ubiquitination and transcription factors (TFs), including both transcriptional activators and repressors. For each of these well characterised protein families, the proportion of annotated family members in the human genome we detected here was >60%. For example, of the 523 kinases in the manually curated kinome ( Manning *et al.*, 2002), 330 (~63%) were identified in our data set ( Figure 3A). This compares, for example, with a total of 349 protein kinases that were previously reported as being expressed at the protein level in the 2014 ‘draft human proteome’, which collated proteome data from multiple human cell types and tissues ( Kim *et al.*, 2014; Wilhelm *et al.*, 2014). Similarly, of the 267 genes encoding phosphatases and phosphatase-regulatory subunits in the human genome ( Sacco *et al.*, 2012), we identified expression at the protein level of 178 (~67%) in this epithelial cell data set ( Figure 3B).

In summary, we conclude that our data set has identified most, and potentially in some cases all, of the members of each of the gene families that are expressed at the protein level in these differentiated human epithelial cells.

### Protein expression levels

**Figure 4.**
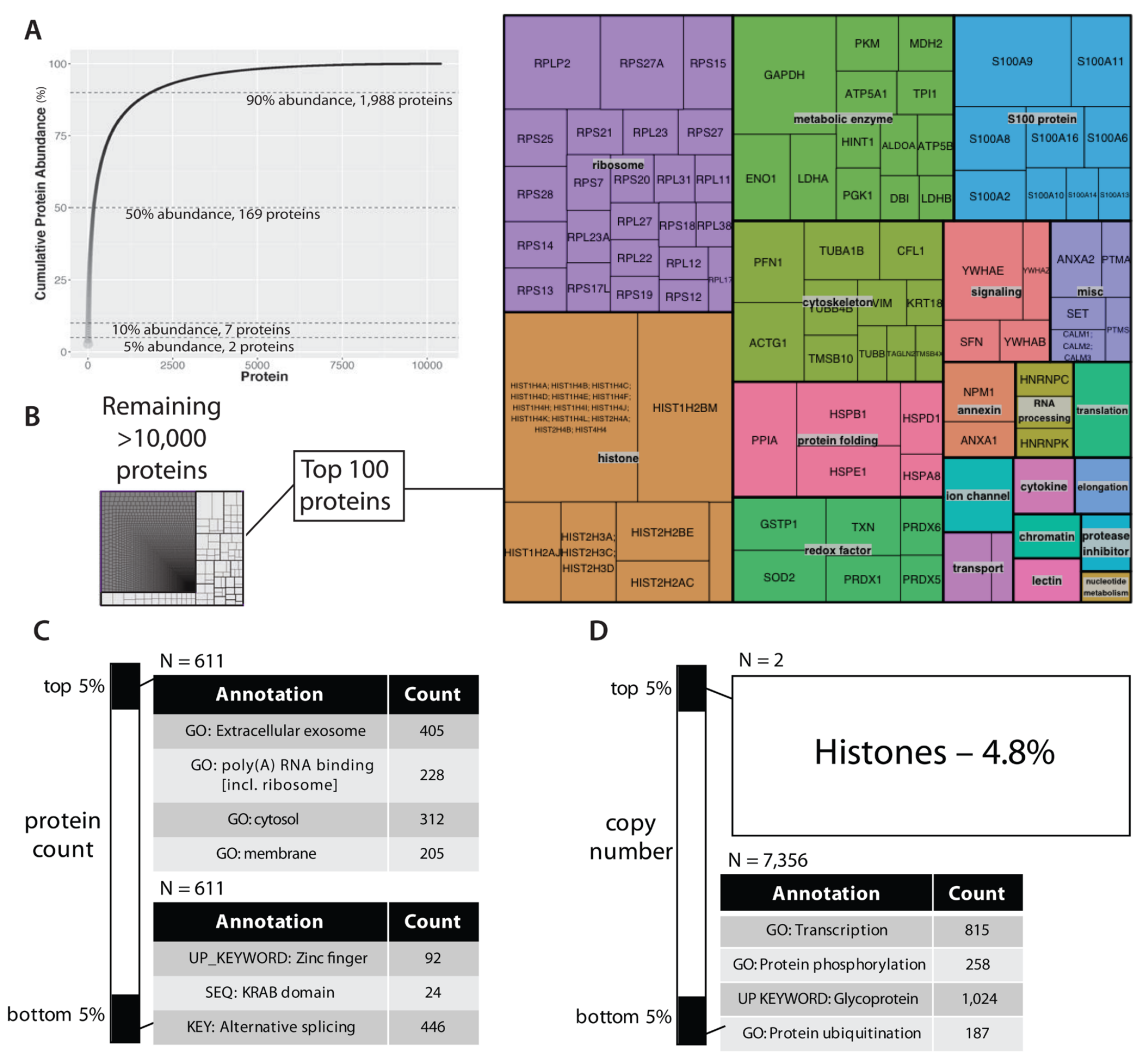
The copy number profile of the untransformed epithelial cell proteome. ( **A**) A plot of cumulative sum of protein abundance versus protein ranking by abundance (most abundant protein on the left). ( **B**) A tree diagram where each internal box represents a different protein and the box size indicates the proportion of total abundance. A zoomed-in view of the most abundant 100 proteins (right panel). ( **C**) Gene ontology analysis based on rank percentiles, comparing the top versus bottom 5% ranked proteins. ( **D**) Gene ontology analysis based on abundance percentiles.

Having established that this data set is of sufficient depth, protein copy numbers could be estimated using the ‘proteome ruler’ approach ( Wisniewski *et al.*, 2014), which normalizes protein abundances to the intensities measured for core nucleosome histones. The estimated protein copy numbers for the basal, untransformed epithelial proteome follow a log-normal distribution (cf. Figure 2D, Supplementary Table 3). Figure 4A shows a plot of cumulative copy number, ranked from highest to lowest copy number protein (left to right). As previously reported for other mammalian cell lines ( Beck *et al.*, 2011; Bekker-Jensen *et al.*, 2017; Hukelmann *et al.*, 2016; Ly *et al.*, 2014; Nagaraj *et al.*, 2011), a small number of proteins constitute the major proportion of the cumulative protein abundance measured. For example, ~5% of the cumulative protein abundance in this epithelial cell line is contributed by histones alone ( Figure 4). Further, the top 169 most abundant proteins make up 50% of the total protein abundance, while the top 1,988 proteins contribute 90% of the cumulative protein abundance ( Figure 4A). The corollary is that the great majority (>85%) of proteins detected, together represent less than 10% of the total protein abundance in the cell.

The top 100 most abundant proteins in this data set (representing ~41% of the cumulative protein abundance), are dominated by ribosomal proteins, histones, metabolic enzymes and cytoskeletal proteins ( Figure 4B). Notably, S100 calcium-binding proteins also contribute significantly to the bulk protein composition of these epithelial cells, contributing ~4% of the total protein by copy number.

To assess any potential relationship between protein copy number and cellular function, we performed enrichment analysis using the DAVID analysis tool v6.8 ( Huang *et al.*, 2009). In addition to gene ontology terms, enrichment analysis by DAVID considers annotations from multiple sources, including structural databases and the UCSB transcription factor binding database. We used two approaches to calculate quantiles, i.e. (i) rank order and (ii) copy number. We then asked whether these protein sets, representing extreme quantiles, either in rank, or copy number, were significantly enriched in any gene annotations.

Using rank quantiles, the top and bottom 5% represent the top and bottom 611 proteins ranked by abundance, respectively. As illustrated in Figure 4C, the top ranked proteins are enriched in ribosomal proteins and proteins that are localised in either extracellular exosomes, or in cytosolic and membrane-associated subcellular compartments. The bottom ranked proteins are enriched in zinc-finger transcription factors, many of which contain the Krueppel-associated box (KRAB) zinc-finger associated domain and also proteins annotated with alternative splicing.

Using copy number quantiles ( Figure 4D), there is a dramatic difference in the protein composition of the highest and lowest 5% protein groups. Thus, the top 5% of protein copies per cell is composed of only histone proteins, whereas the bottom 5% is composed of a diverse array of >7,300 proteins. This low abundance group is enriched in transcription factors, kinases (UniProt keyword: ‘protein phosphorylation’), glycoproteins and enzymes that add ubiquitin to proteins. For example, of the ~330 kinases detected, 258 (~78%) are in the bottom 5% protein copy number bin.

### Steady state protein turnover

**Figure 5.**
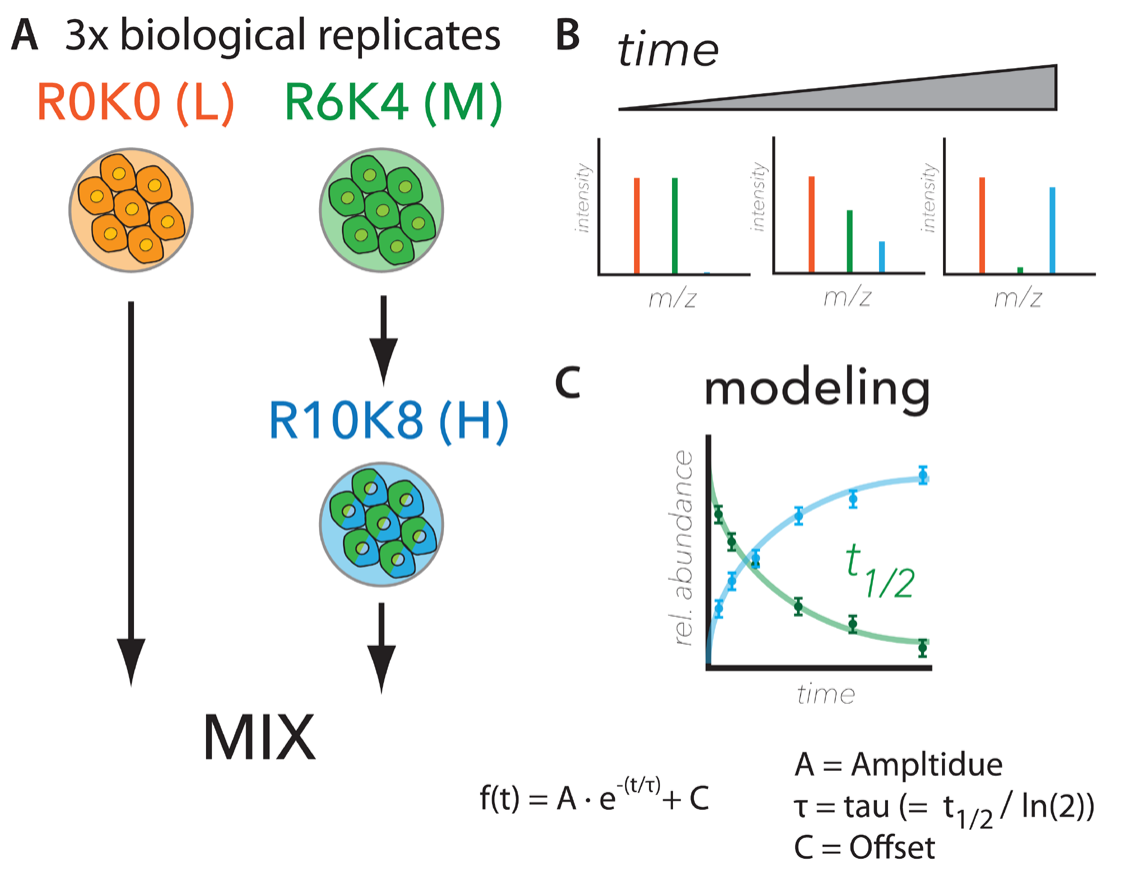
Workflow for comprehensive analysis of protein synthesis and degradation. ( **A**) Metabolic labelling strategy, ( **B**) expected ratiometric measurements, and ( **C**) modelling for measuring protein turnover. The experimental design used to measure protein half-life proteome-wide is largely based on ( Boisvert *et al.*, 2012). Briefly, cells were labelled with Arg0-Lys0 (R0K0, “Light”) or Arg6-Lys4 (R6K4, “Medium”) stable isotope labelling with amino acids in cell culture (SILAC) media. The R6K4 labelled cells were then switched to Arg10-Lys8 (R10K8, “Heavy”) media and cultured for seven time points (i.e., 1, 3, 6, 12, 24, 48 and 72 hrs) before mixing with R0K0 and cell harvest. A schematic is shown of the expected SILAC MS data (top right panel). Data were modelled using an exponential fit, where t _1/2_ is half-life.

We used our previously described control + pulse-SILAC approach ( Ahmad *et al.*, 2012; Boisvert *et al.*, 2012) to measure steady-state protein synthesis and degradation rates in both control, untransformed epithelial cells and in the same cell line after it had been transformed by activation of v-Src kinase activity for 48hrs (described above as Exp B, cf. Figure 2). Briefly, the pulse-labeling protocol ( Figure 5) involved differentially labeling MCF10A Src-ER cells with isotopologues of arginine and lysine, i.e. either Arg0-Lys0 (R0K0, ‘light’, L), or Arg6-Lys4 (R6K4, ‘medium’, M). The culture media for fully R6K4 (M) labelled cells was then replaced with Arg10-Lys8 (R10K8, ‘heavy’, H) media. At 1, 3, 6, 12, 24, 48, and 72 hrs after the media switch, cells were harvested, mixed with equal numbers of R0K0 (L) cells at each time point, then the combined control + pulsed cells were lysed and processed for in-depth, MS-based proteomic analysis ( Figure 5A).

Using this experimental design, the anticipated MS measurements over time are: (i) decreasing signal from R6K4-labeled peptides, due to the decrease in intracellular protein levels resulting from either degradation, or via secretion, and (ii) increasing signal from the R10K8-labelled peptides, due to increasing intracellular protein levels resulting from nascent protein synthesis ( Figure 5B). The R0K0-labelled peptides, which are mixed in at a constant 50% level at each time point, are used as a reference internal standard that normalizes the data for potential technical variation, e.g. associated with either sample processing and/or cell count precision, etc. ( Ahmad *et al.*, 2012; Boisvert *et al.*, 2012).

Synthesis and degradation rates were estimated by modeling the change in isotope-labeled peptide ratios over time as an exponential fit, as shown in Figure 5C. The model assumes steady state equilibrium conditions, where the rate of increase is counterbalanced with the rate of decrease, leading to stable intracellular protein levels ( Boisvert *et al.*, 2012). In brief, the model has three parameters: amplitude (A), tau (equal to half-life / ln(2)), and offset (C). A is the difference in the ratios of pulsed protein abundance/control (as judged from SILAC data) between t = 0 and t = 72 hrs. C is the estimated asymptotic limit of the exponential curve, resulting from the combined effect of amino acid recycling, as previously described ( Boisvert *et al.*, 2012; Jovanovic *et al.*, 2015) and the average proportion of protein that is refractive to degradation over the timescale of the experiment, i.e., 72 hrs (see Methods). Errors in the three parameters were determined both from individual peptide measurements and from comparison of the three biological replicates. Fit qualities were estimated separately using chi-squared, least-squares regression (r ^2^) and root-mean-squared (rms) analyses ( Supplementary Table 4). For further description of the model, see Methods.

Kinetic half-life data were obtained for 9,013 proteins in the combined data set (i.e., basal + transformed, *vide infra*). Under basal conditions, kinetic data were measured for 8,682 proteins, corresponding to ~60% of the different protein species detected in this cell line ( *vide supra*). The proteins for which kinetic data were measured span a wide dynamic range of expression levels, ranging between an estimated average of <500 to >96 million, copies per cell. As discussed further below, this represents measurements of the turnover of >97% of the total protein molecules in the untransformed epithelial cells.

**Figure 6.**
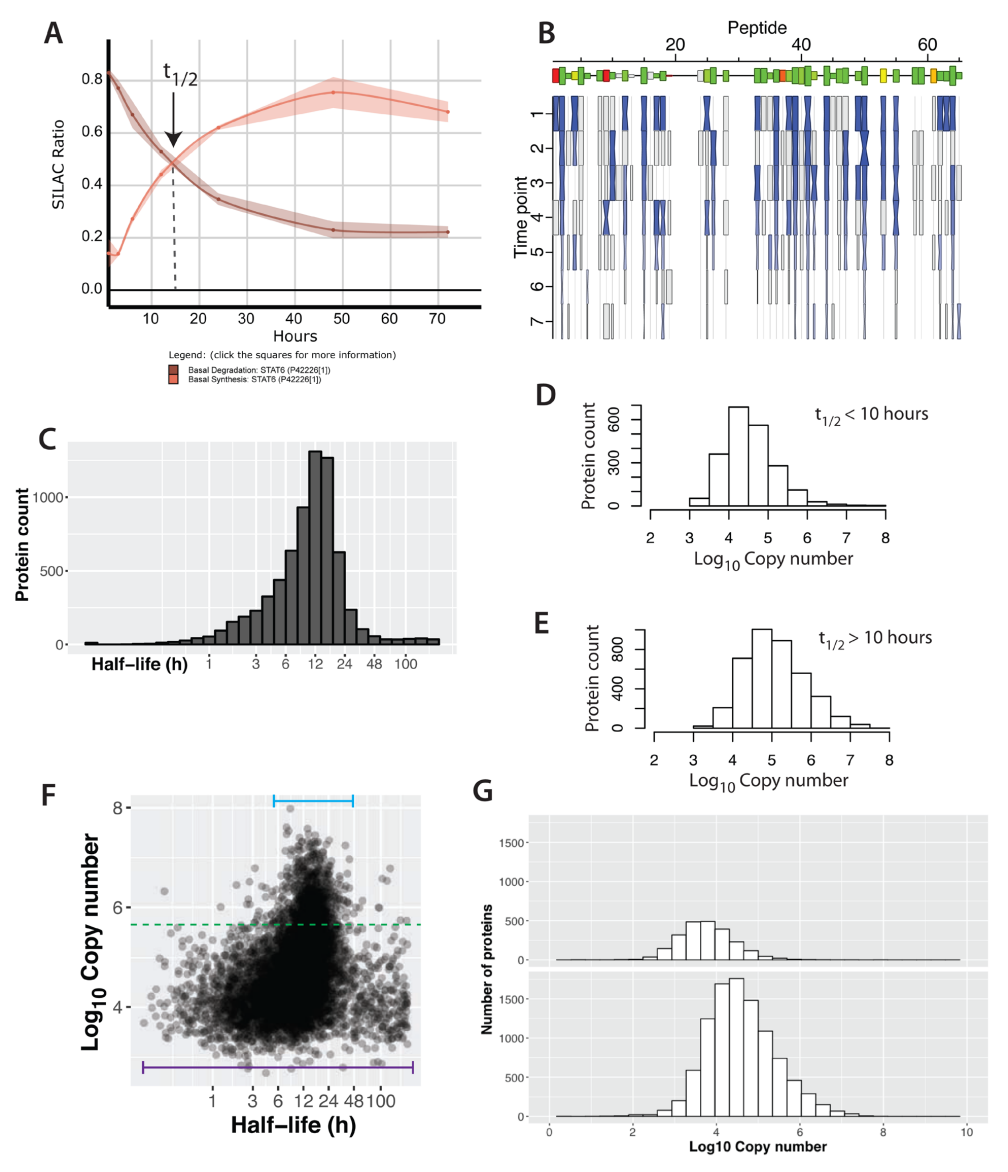
Protein half-lives in a contact-inhibited epithelial cell monolayer. ( **A**) A plot showing the synthesis (red) and degradation (brown) curves for STAT6. The lines and ribbons show the mean ratios and standard errors, respectively, from three biological replicates. The half-life point is indicated with an arrow. ( **B**) A ‘carrot’ plot showing the per-peptide analysis of half-life. The x-axis is peptide number (ordered by sequence position). Size of the box indicates the intensity for the peptide and the colour indicates the correlation between the individual peptide half-life versus the mean aggregate half-life for the protein (green – high correlation, red – low correlation). The y-axis is time and the hourglass shapes represent individual ratio measurements, with centre widths indicating standard error, and end widths and blue shading indicating the mean ratio across three biological replicates. Grey boxes indicate single replicate measurements. ( **C**) The distribution of half-lives measured in the basal state. ( **D**, **E**) The distribution of protein abundance for proteins that have either half-lives > 10 hrs ( **D**) or half-lives < 10 hrs ( **E**). ( **F**) A plot of log _10_ copy number versus half-life (x-axis is log _2_ space). The bars illustrate the range of half-lives for the highest and lowest copy number proteins. ( **G**) Copy number distributions for proteins whose half-lives could not be determined (top) versus proteins whose half-lives were measured (bottom).

Figure 6A shows an example of these kinetic data for the protein STAT6, which is a transcription factor associated with interleukin (e.g. IL-4 and IL-13) signalling ( Goenka & Kaplan, 2011) that is expressed here at a typical intermediate level, (i.e. ~70,000 copies per cell). We detect expression of all seven known STAT transcription factors in these epithelial cells, albeit at varying abundance levels. STAT6 is amongst the three most highly expressed STAT factors. Synthesis and degradation curves for STAT6 are plotted, showing errors as ribbons, with the crossover point of these curves identifying the half-life (t _1/2_ = 11 ± 0.5 hr). This plot is calculated as the mean of each peptide assigned to the STAT6 protein for which values were measured. Figure 6B shows the corresponding data for each of the individual peptides (N=46) that were mapped to the STAT6 protein. Most of these peptides show high correlation in the values of their individual half-lives (green boxes), with the mean half-life calculated for the STAT6 protein.

### Protein abundance and half-life profiles across the proteome

In untransformed MCF10A Src-ER cells, protein half-lives show a log-normal distribution, with a median half-life of ~11.6 hrs ( Figure 6C). An example of a protein with a short half-life in this data set is the hypoxia-induced angiogenesis factor ANGPTL4, (t _1/2_ 0.42 ± 0.1 hrs). Conversely, the longest protein half-lives estimated from this data set exceeded 200 hr. An arbitrary limit was set for tau (300 h), which corresponds to a half-life of 208 hr. As expected, the error associated with very long half-life measurements is generally large, because they significantly exceed the value of the final time point of the experimental time course (i.e. 72 hr).

These data show that many shorter-lived proteins (t _1/2_ < 10 hours) have relatively low copy numbers ( Figure 6D; median copy number 30,000). In contrast, longer-lived (t _1/2_ > 10 hours) proteins, on average show approximately three-fold higher copy number ( Figure 6E; median copy number ~100,000). These results support the hypothesis that proteins with shorter half-lives in these epithelial cells tend to show lower steady state expression levels. The trend is robust towards a range of quality thresholds for the exponential fitting (e.g., r ^2^), making it unlikely that that these differences are due to variation in fit quality.

However, when half-life values are analysed across the whole proteome, rather than considering specifically the highest and lowest half-life bins, protein half-life has only a poor correlation overall with protein intensity (r ~ 0.3), as shown in Figure 6F. A likely explanation for this observation is that the preponderance of lower abundance proteins in the proteome show a much wider distribution of half-life values than the smaller number of high abundance proteins ( Figure 6F, cf. purple and blue horizontal bars).

### Abundance weighted proteome turnover

Considering the high dynamic range of protein expression levels measured for the epithelial proteome (cf. Figure 4A), we next evaluated how the measurement of protein turnover is impacted by copy number. As predicted, proteins whose half-lives were not determined show a bias towards low copy number proteins ( Figure 6G). However, the 8,682 proteins for which we have measured half-life values in the untransformed epithelial cells, (corresponding to ~62% of the basal proteome), accounts for >97% of the protein molecules in the cell. This striking observation suggests an alternative approach for evaluating the rate at which the global cell proteome turns over. Specifically, since we determined that the protein products of only 169 genes account for ~50% of the total protein abundance, the half-life values of this small subset of all the genes expressing proteins will disproportionately affect the rate at which the total number of protein molecules in the cell are turned over.

Therefore, we next calculated an ‘abundance weighted’ average proteome turnover value, taking into account the copy numbers of each expressed protein for which a half-life was measured. This abundance weighted average turnover value provides an estimate of the intracellular half-life of a theoretical population of ‘average’ protein molecules in the cell.

For untransformed epithelial cells, the abundance weighted median protein half-life value is 14.2 hrs, as compared with the unweighted median value of 11.6 hrs (i.e. calculated from the individual protein half-life values measured without reference to their abundance), a difference of ~20%. Calculation of the mean, rather than median, half-life value across all of the proteins measured similarly shows an increase (~10%) in the average protein turnover value, when abundance weighting is taken into account (weighted mean turnover, ~15.5 hrs).

### Profiling protein half-life with protein function

**Figure 7.**
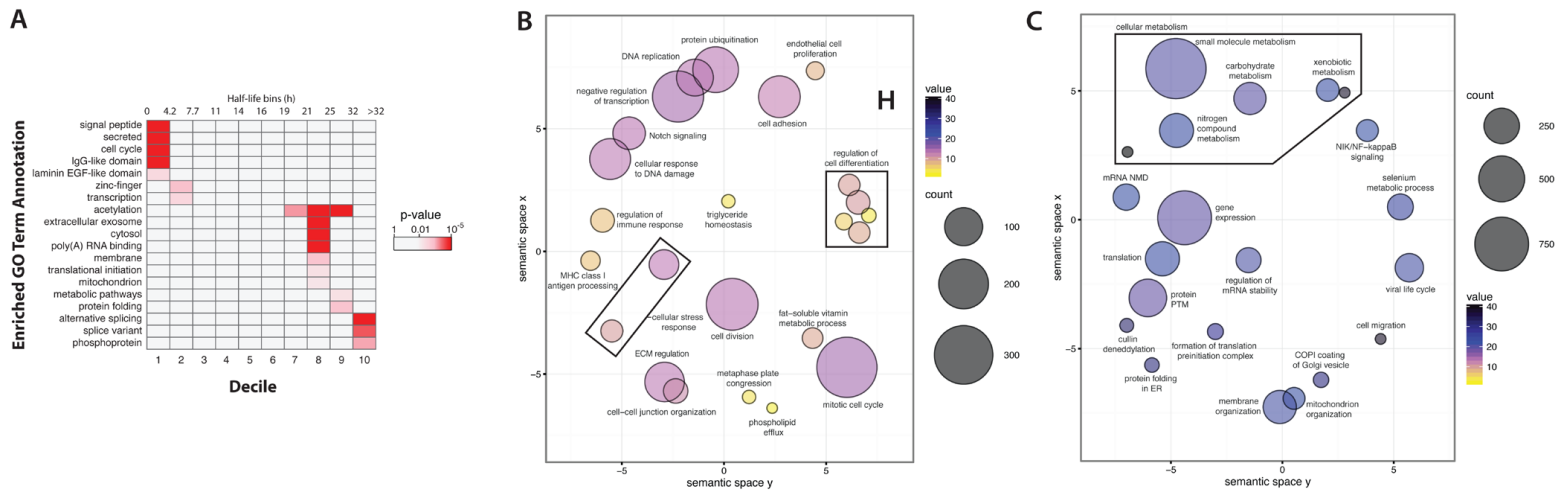
Gene ontology annotation analysis of short-lived and long-lived proteins. ( **A**) A heatmap of gene ontology annotation versus proteins, binned into 10 deciles. The colour indicates the magnitude of the p-value, i.e. the significance of the enrichment. ( **B**, **C**) ReviGO plots with enriched GO ontology terms associated with short-lived ( **B**) and long-lived ( **C**) proteins. The bubble colour and size represent half-life and number of proteins, respectively.

To investigate potential links between the cellular function and half-life of proteins, we binned the protein half-life distribution into deciles and asked whether any functional annotations were statistically enriched in each bin. Figure 7A shows a heatmap of the annotations that had p-value False Discovery Rates (FDR) of 0.01 or less. The decile containing the shortest-lived proteins shows an enrichment in proteins that are secreted and/or have a secretion signal peptide, cell cycle proteins, IgG-domain containing proteins and laminin proteins. This suggests that in this analysis, with little or no cell division during the time course of the pulse-SILAC experiment and with the cells showing contact inhibition, protein secretion is a significant mechanism contributing to proteins measured with short *intracellular* half-lives.

Interestingly, there is a difference in the categories of proteins enriched between the first (<4.2 hrs) and second (4.2–7.7 hrs) deciles. Thus, the second decile is enriched in zinc finger domain containing proteins and transcription factors, which is not seen in the first decile. In contrast, the third through sixth deciles (containing proteins with half-lives from 7.7–19 hrs) do not show any significant annotation enrichments. This represents the large group of proteins with half-life values centred around the median proteome half-life. For proteins with longer half-lives, enrichment for extracellular exosome associated proteins is detected across the seventh through ninth deciles, representing proteins with half-lives between 19–32 hrs.

The eighth decile (21–25 hrs), also contains many of the ribosomal proteins, along with enrichment of annotation terms such as translational initiation and poly(A) RNA binding. This is consistent with the known long half-lives of proteins in the cytoplasmic translation machinery ( Boisvert *et al.*, 2012; Lam *et al.*, 2007). Interestingly, membrane and mitochondrial-associated proteins are also enriched in the eighth decile. The ninth decile (25–32 hrs), is enriched in metabolic enzymes and protein folding chaperones. Finally, the longest-lived proteins (>32 hrs), are associated with annotations for alternative spliced variants and phosphoproteins. Interestingly, these very long half-life proteins tend to be present in relatively low copy number, as discussed below.

A bootstrap-based gene ontology (GO) enrichment analysis was performed to compare the annotations enriched in short-lived, versus long-lived proteins (see Methods). In brief, a per-GO term mean half-life was calculated for annotated proteins. Iteration over each GO term creates a distribution of mean half-lives. Distribution extremes were identified using permutation-based scoring (Pscore). GO terms with Pscore < 0.001 or > 0.999 indicate enrichment in significantly short-lived and long-lived proteins, respectively. Enriched GO terms were then visualized using ReviGO ( Supek *et al.*, 2011) to group GO terms with similar meaning. Figures 7B and 7C show the enriched GO terms for short-lived and long-lived proteins, respectively.

As shown in Figure 7B, short-lived proteins are significantly enriched in annotations associated with cell division (mitotic cell cycle, DNA replication), the cellular stress response, cell-cell adhesion, cell-cell communication (Notch signalling), MHC class I antigen presentation, and regulation of cellular differentiation. In contrast, long-lived proteins are associated with different functional annotation terms. Thus, long-lived proteins are enriched in terms for mitochondrial organization factors, metabolic enzymes (many of which localize to mitochondria) and proteins regulating gene expression ( Figure 7C). For example, enzymes in the glucose metabolic pathway have an average t _1/2_ of 15.8 hr (unweighted), compared with a proteome-wide, unweighted median value of 11.6 hr.

### Profiling protein half-life with protein complex formation

We previously observed that average protein half-life values measured in total cell extracts can mask situations where the same protein shows differential stability in separate subcellular compartments. This was shown for several protein complexes, including RNA polymerases as well as ribosomal subunits, analysed in cancer cell lines ( Boisvert *et al.*, 2012; Boulon *et al.*, 2010; Lam *et al.*, 2007). We therefore analysed the current data set for a relationship between protein stability and membership of protein complexes. To test this, we took the CORUM database of human complexes ( Ruepp *et al.*, 2008) and asked whether there was any difference in the similarity of protein half-lives among subunits ascribed to the same complex, as compared with the same number of proteins chosen at random.

**Figure 8.**
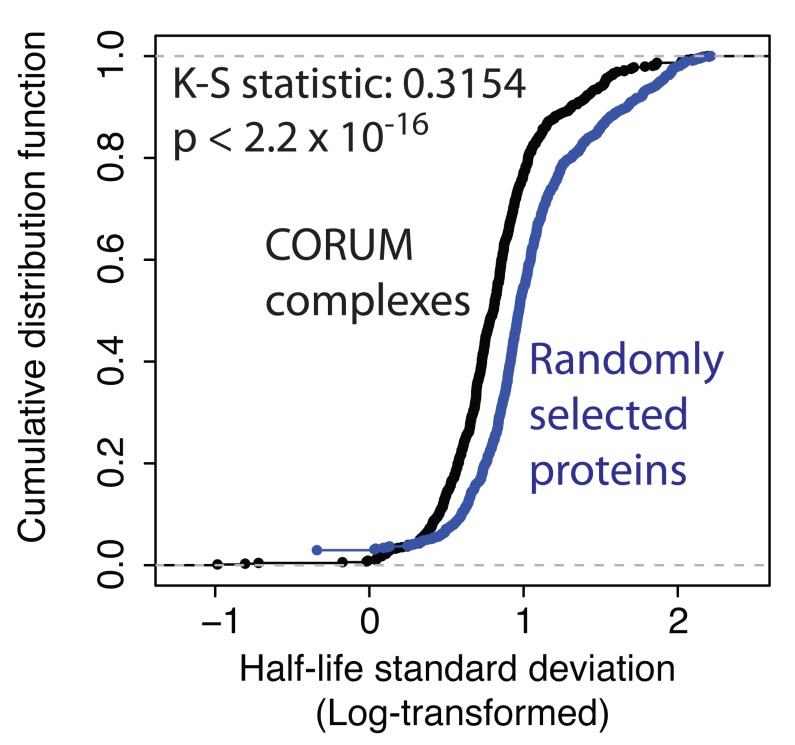
Proteins belonging to the same complex are more likely to have similar half-lives. The cumulative distribution function for standard deviation in half-life, for proteins either belonging to the same complex, as listed in CORUM (black), or proteins randomly grouped into decoy pseudo-complexes, identically-sized to CORUM (blue).

Figure 8 shows a plot of the cumulative distribution functions of calculated variances, comparing half-lives of subunits within a complex (black line), with proteins chosen at random (see Methods section for bootstrapping procedure) from the epithelial proteome (blue line). The two distributions show a statistically significant difference, with proteins in the same CORUM complex having smaller variance in half-life values than seen for random protein sets. These data thus support the hypothesis that proteins that associate in the same complex can be co-regulated by mechanisms affecting protein stability ( McShane *et al.*, 2016).

### Src kinase-induced remodeling of protein expression (Exp A)

**Figure 9.**
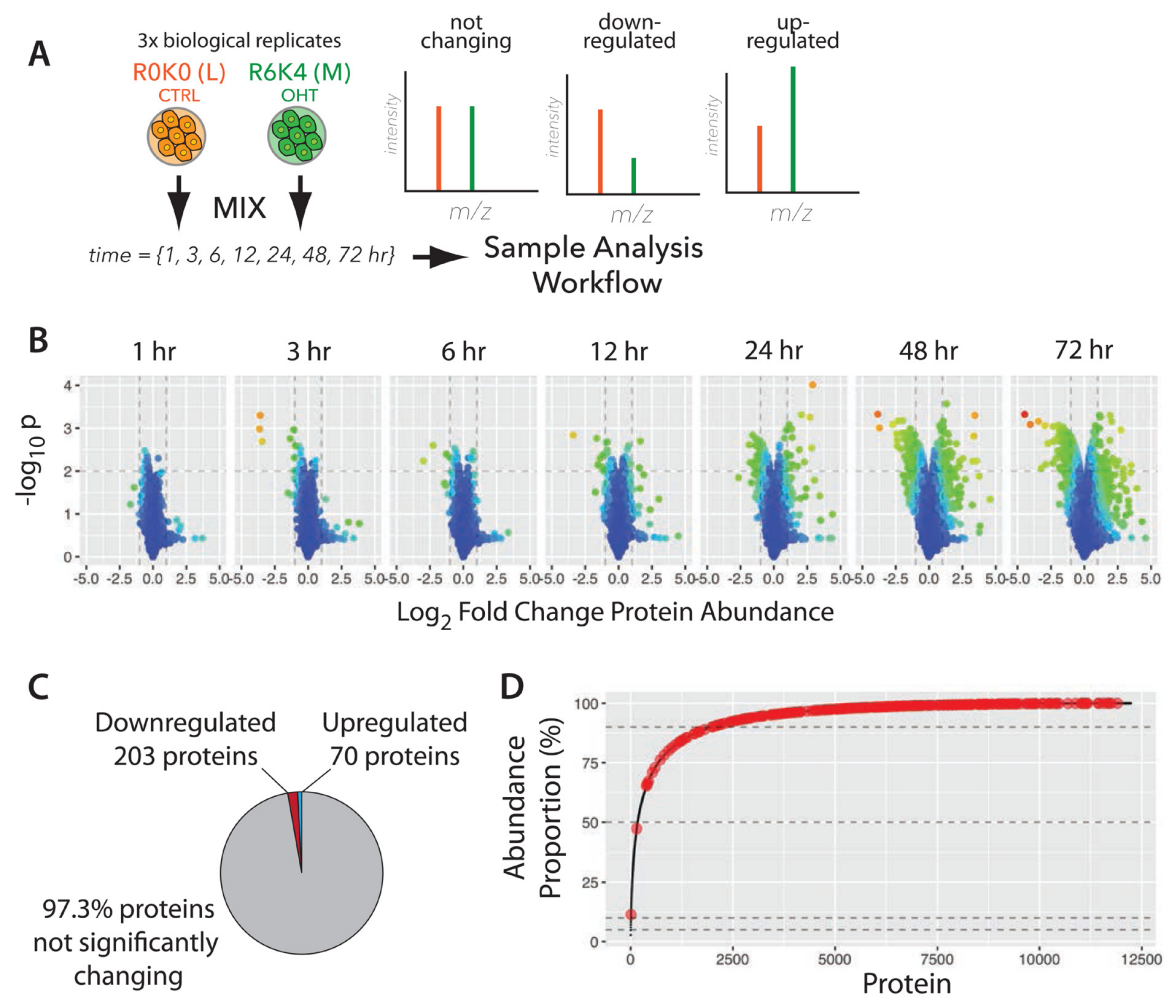
Dynamic remodeling of the proteomic landscape in response to v-Src kinase activation. ( **A**) The experimental design for measuring protein abundance changes. R0K0 and R6K4 labelled cells were treated either with vehicle control, or 4-hydroxytamoxifen (4-OHT) and incubated for the indicated times, prior to mixing and harvest. SILAC MS data expected from the time course are illustrated (right). ( **B**) ‘Volcano’ plots of –log _10_ p-value versus log _2_ fold change for the seven time points. ( **C**) Proportion of downregulated, upregulated, and not significantly changing proteins. ( **D**) Cumulative abundance plot (cf. Figure 4A) with significantly changing proteins highlighted in red.

Having characterized the proteome of untransformed epithelial cells, we next analysed how this proteome is affected by cell transformation induced by activating v-Src kinase, starting with the effect on protein abundance. We measured protein expression at seven time points, from 1–72 hrs, during which the MCF10A Src-ER cells undergo profound phenotypic transformation (cf. Figure 2A, Exp A). Ratiometric SILAC-based measurements ( Ong & Mann, 2006), were performed in biological triplicate at each of the seven time points ( Figure 9A). Proteins were classified as changing ‘significantly’ during this time course if their abundance altered by at least 2-fold, with an associated p-value <0.01 (using a shrink-variance t-test, see Methods and Supplementary Table 5).

**Figure 10.**
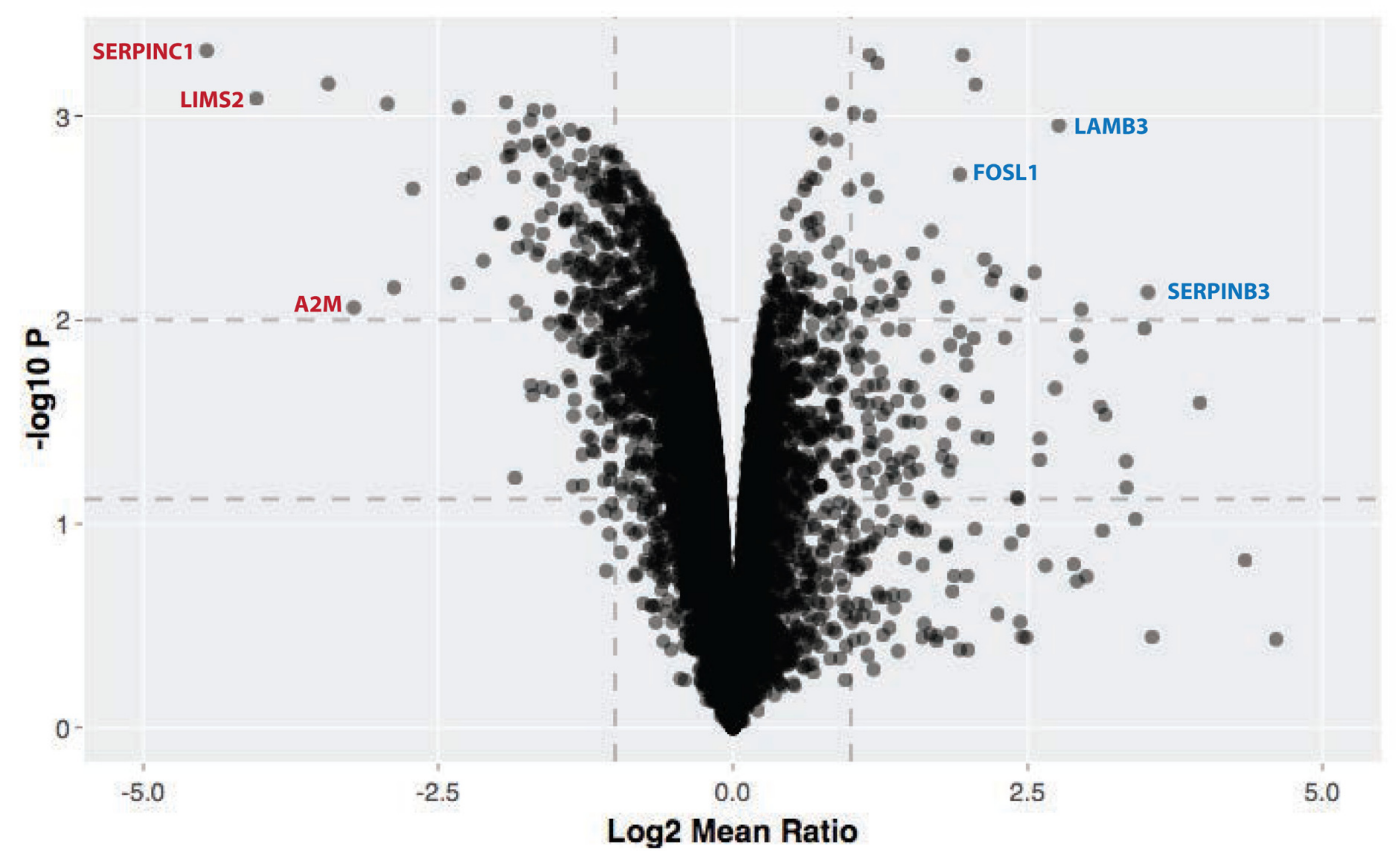
Proteomic changes 72 h after Src kinase activation. Interactive volcano plot showing -log10 p-value versus log2 fold change for the 72 h timepoint. Horizontal dotted lines indicate 0.05 and 0.01 significance, and vertical dotted lines indicate 2-fold change. The online version of this figure is interactive.

We observe that activation of v-Src kinase activity promotes reproducible changes in the abundances of only a small subset of the epithelial cell proteins (~2.7% of total proteome), as shown in the respective volcano plots for each time point ( Figure 9B). An interactive volcano plot showing data for the 72 h timepoint is shown in Figure 10. The majority of affected proteins show a reduction in abundance, with the first responses detected at the 3 hr time point. These ‘immediate early’ decreasing proteins include protein phosphatase 1D (PPM1D), which has been shown to inactivate the checkpoint proteins p53 and Chk1 ( Lu *et al.*, 2005) and the sprouty homologue 4 (SPRY4), which suppresses insulin receptor- and epidermal growth factor-dependent ERK/MAPK signalling ( Sasaki *et al.*, 2003).

At every time point, downregulated proteins outnumbered upregulated proteins. At these cut-off values, 273 proteins show differential expression with 203 proteins downregulated and 70 upregulated ( Figure 9C). A less stringent cut-off of 0.05 increases the total number of differentially expressed proteins to 456 ( Supplementary Table 5). Proteins just over the p <0.01 threshold include NFKIA (NF-kappa-B inhibitor), which shows decreased abundance at 24 hr.

Due to the previously described high dynamic range of protein expression levels (see the cumulative abundance plots discussed above; cf. Figure 4A), the ~2.7% of proteins showing a significant abundance change during cell transformation could represent either a relatively minor, or a large fraction, of the total protein copies in the cell. Therefore, it was important to evaluate the observed changes in protein abundance in the context of protein copy number. This analysis showed that the proteins significantly changing in abundance represent only ~1.5% of the total protein molecules in the cell. Interestingly, the great majority of the significantly changing proteins are in the lowest abundance region (i.e. <10%) of the cumulative protein abundance curve (cf. Figure 4A and Figure 9D).

We conclude that the activation of Src kinase activity predominantly affects expression of low abundance class proteins, many of which are not present in the TCGA reverse phase protein array dataset ( Akbani *et al.*, 2014) and also may have escaped detection in previous analyses.

### Protein response kinetics

**Figure 11.**
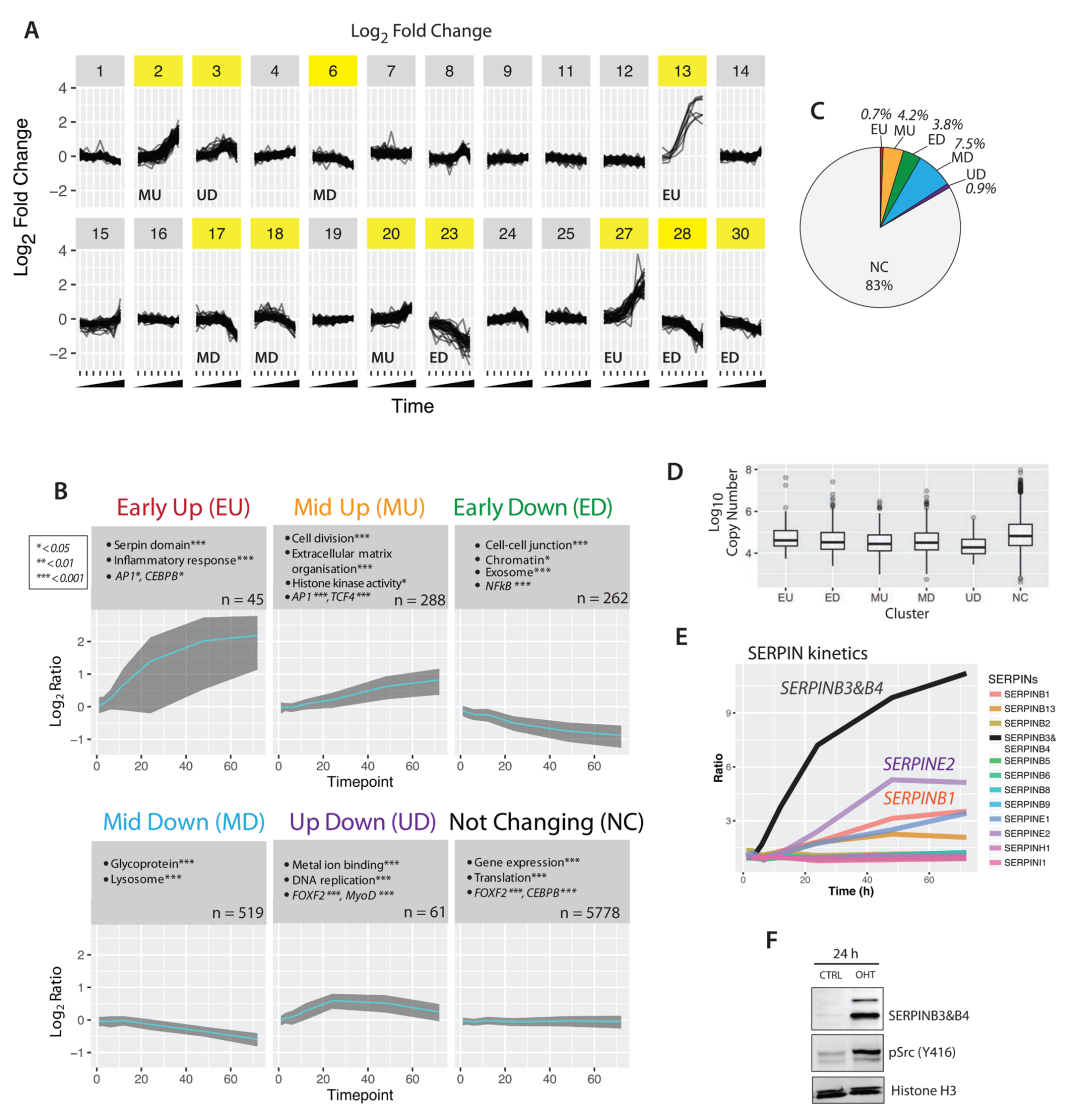
Clustering of Src response kinetics. ( **A**) Expression profiles for all proteins with complete time course data (n = 6,890) were grouped into 30 clusters, using k-means. ( **B**) Gene ontology enrichment analysis of six agglomerated clusters. The enrichment p-values are indicated by asterisks (* < 0.01, ** < 0.001 *** << 0.001), and n is the number of proteins in each cluster/group. ( **C**) Proportion of each cluster relative to the entire proteome data set. ( **D**) Box plots of protein copy number versus protein cluster. **E**) Line graph of serine protease inhibitor proteins measured across the complete time course. SERPINB3&B4 (black) increases rapidly upon v-Src activation. **F**) Immunoblot analysis of control- and 4-OHT-treated lysates shows upregulation of SERPINB3&B4 protein upon v-Src activation, consistent with the MS measurements.

We next used clustering analysis to characterize patterns of protein abundance changes across the time course of cell transformation. In this case proteins that behave similarly – but that individually may not meet the stringent cut-offs established above – are grouped together, increasing the analysis sensitivity. To focus specifically on the highest quality data in this study, average temporal profiles were calculated only for proteins that were detected both (a) in all three biological replicates and (b) at all seven time points. This very high stringency selection resulted in 6,890 ‘complete’ protein profiles. These highest quality data profiles were grouped into clusters, using k-means ( Figure 11A).

The number of clusters (k) was chosen as 30, corresponding to the point where the decrease in within-group sum of squares became asymptotic with increasing *k*. Clusters where the maximum fold change across the time-course was 3-fold or greater (clusters highlighted in yellow in Figure 11A) were manually agglomerated, based on their mean profiles, into six qualitatively similar groups: ‘early up’ (EU), ‘mid up’ (MU), early down’ (ED), ‘mid down’ (MD), ‘up down’ (UD), and ‘not changing' (NC). Figure 11B shows the mean profile (cyan) and standard deviation (grey ribbon) for each cluster.

Consistent with our observation that transformation affects the abundance of only a small fraction of the epithelial proteome, the largest number of proteins (~83%) belonged to the ‘non-changing’ (NC) cluster ( Figure 11C). There were varying numbers of proteins mapped to each respective varying cluster. However, despite differences in the numbers of proteins in each cluster, they each had a similar distribution of protein copy numbers ( Figure 11D).

Next, we asked whether specific biological functions were differentially represented in the respective clusters. To do this, each cluster was subjected to gene ontology term enrichment analysis, as described above (cf. Figure 4). Selected GO terms with high enrichment p-values are shown in the grey boxes in Figure 11B. Each of the clusters appear to contain functionally distinct proteins, as shown by their differential enrichment of gene functions and predicted transcription factor binding.

For example, the EU cluster is enriched in the serine protease inhibitor (serpin) domain and proteins involved in the inflammatory response. Members of the EU cluster include the serine protease inhibitors B3 and B4 (SERPINB3 & SERPINB4). While we detect expression of 13 members of the serpin protein family in untransformed cells (cf. Supplementary Table 1), only 5 of these serpins show an increased abundance after v-Src activation ( Figure 11E). Of these, serpin B3/B4 shows the most rapid response and the largest overall increase of abundance, reaching ~10 fold or greater abundance by 72 hrs post Src activation. This dramatic and rapid change in serpin B3/B4 seen by MS analysis was also confirmed by independent detection of serpin B3/B4 using protein blotting ( Figure 11F).

Early downregulated (ED) proteins are characterized by factors involved in cell-cell junctions, exosome constituents and genomic targets of the NF-kappa-B transcription factor. Later upregulated events (i.e., ‘mid up’) are enriched in proteins involved in cell division and extracellular matrix organization. Late downregulated events are enriched in lysosomal proteins and glycoproteins. Notably, the great majority of epithelial cell proteins are in the ‘not changing’ group, which is enriched in factors associated with housekeeping functions, e.g. bulk gene expression and protein translation.

### Dynamics of proteome remodeling induced by activated Src kinase

This comprehensive proteome analysis provides an opportunity to compare how specific signalling pathways and protein families respond to Src kinase activation. To illustrate this, we highlight here selected examples of the responses of individual pathways and protein families. Further analysis on a wider range of pathways and protein families can be performed using the Encyclopaedia of Proteome Dynamics (EPD), as described below.

**Figure 12.**
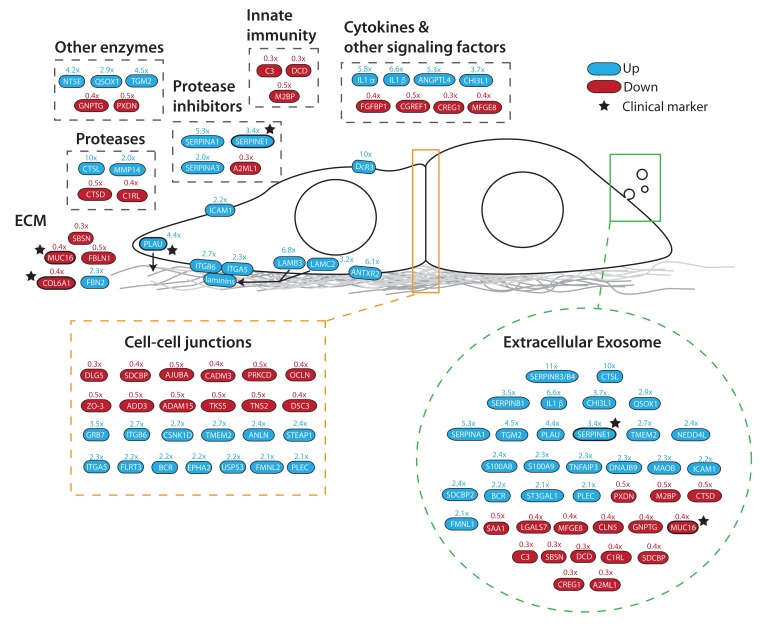
Src activation remodels cell-cell and cell-matrix interactions. Diagram highlighting significantly changing proteins associated with extracellular communication and remodeling. Colours indicate direction of change (blue – upregulated, red – downregulated). Stars indicate current clinical markers. Arrows indicate relationships, e.g. secretion of laminins (LAMB3, LAMC2) and plasminogen activator (PLAU).

The data show that cell transformation is accompanied by an increase in the abundance of a subset of secreted proteins and extracellular matrix (ECM) remodelling factors ( Figure 12). For example, Src-responsive ECM components include laminin proteins (LAMB3, LAMC2), cell surface receptors that interact with ECM, such as integrins (ITGB, ITGA5), enzymes that remodel ECM (PLAU, MMP14) and transcription factors that have been shown to play a role in regulating ECM factors (DLG5).

Many of the clustered proteins detected to change abundance by 2-fold or more have been previously shown to associate with multivesicular bodies and extracellular exosomes, as annotated by GO and UniProt. For example, changes in exosome-associated proteins include an increase in the abundance of a subset of serine protease inhibitors (i.e., serpins B1/B3/B4/E1/A1, Figure 11E), an increase in IL1alpha and a decrease in the abundance of the innate immunity factors C3, DCD & M2BP ( Figure 12). Many of these changes are detected within 12 hrs.

**Figure 13.**
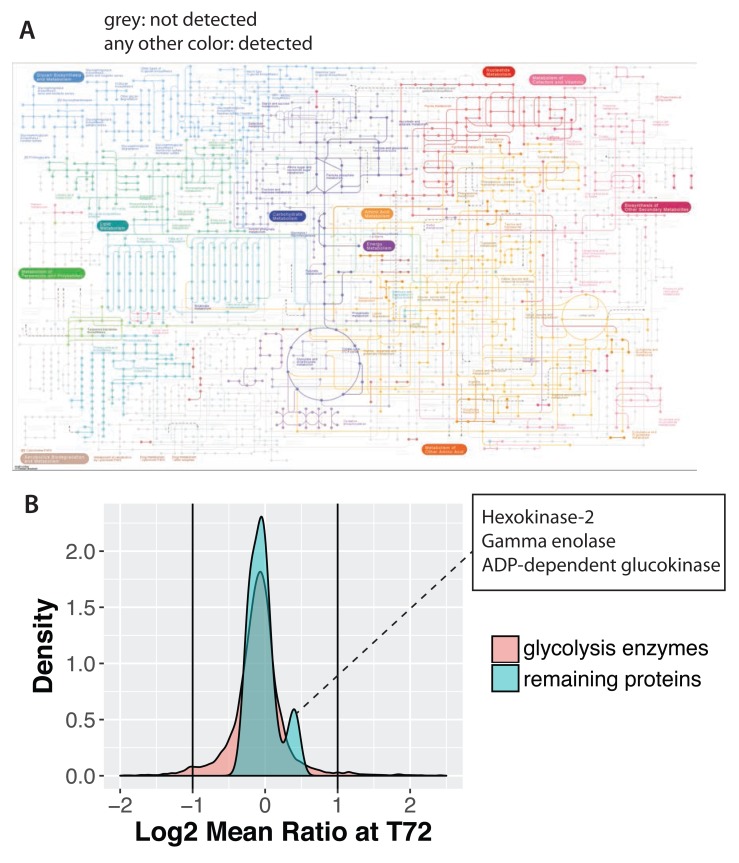
Src has minimal effect on metabolic enzyme abundances. ( **A**) KEGG metabolic map showing the metabolic enzymes detected in this data set as colour nodes and connections. Enzymes not detected are shown in grey. ( **B**) Log ratio distributions of glycolysis enzymes, versus the total proteome.

We looked for evidence of v-Src-mediated transformation triggering significant changes in enzyme abundances, consistent with rewiring in central metabolic pathways. Such changes could provide evidence of potential mechanisms involved in the Warburg effect and would be consistent also with our observations that 4-OHT-treated cells produce more lactate compared to control cells (unpublished observations, Endo and Ly). Our total data set detects expression of most enzymes (~72%) in the KEGG map of metabolism (KEGG, hsa01100), including essentially all major isoforms of enzymes that drive glycolysis, the TCA cycle and oxidative phosphorylation ( Figure 13A). In contrast, the enzymes in KEGG that we do not detect are mainly associated with pathways at the periphery of the KEGG metabolic map, e.g., connected with the metabolism of xenobiotics, terpenoids, chlorophylls and porphyrins.

In considering the impact of Src activation on cell metabolism, we note that several of the enzymes involved in steroid and cholesterol metabolism change in abundance by 2-fold or more, including upregulation of the low-density lipoprotein receptor (LDLR) and enzymes involved in catabolic steroid recycling (i.e., AKR1C2 & HSD11B1). Additionally, the glucose transporter GLUT3 increases by 2-fold. Interestingly, however, the core metabolic enzymes that drive cellular production of ATP show little to no change in abundance at any of the time points following activation of v-Src kinase activity. For example, the majority of glycolytic enzymes show no change, although a small subset of 3 enzymes (i.e., gamma enolase, hexokinase-2 and ADP-dependent glucokinase), show a small percentage increase of ~25–40% ( Figure 13B, second peak on right shoulder of distribution). Further work is required to determine whether these modest abundance changes in a subset of enzymes in the glycolysis pathway contribute to the changes in glycolytic activity that occur in Src-transformed cells, or whether alternative mechanisms, such as changes in either phosphorylation, or other post-translational modifications, are predominantly responsible.

**Figure 14.**
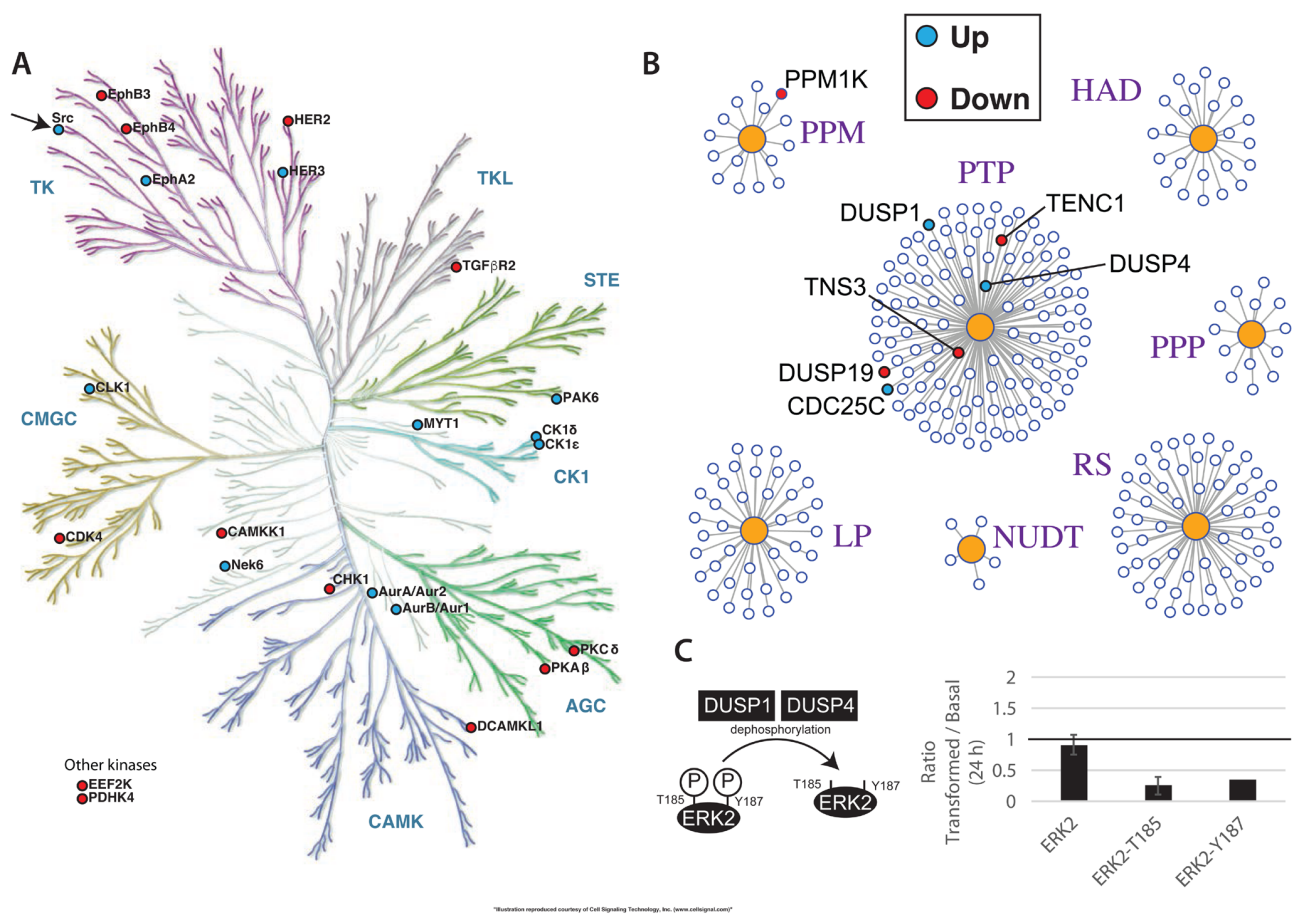
Src-mediated remodeling of epithelial kinome and phosphatome. Illustration of ( **A**) the human kinome and ( **B**) the phosphatome, with significantly changing proteins indicated by filled circles. ( **C**) Schematic of the regulation of ERK2 phosphorylation by DUSP1 and DUSP4 (left panel) and the measured ratios of total ERK2 protein, pERK2-T185, and pERK2-Y187 (right panel).

As shown above, we detected protein expression of ~63% of the human kinome in this data set (cf. Figure 3). The kinetic data show that only a minor fraction of the kinases expressed in untransformed cells change in abundance after Src activation, as illustrated for the 72 hr time point ( Figure 14A). The kinases that change in abundance include HER-family, Ephrin receptor, Aurora and casein kinases. Interestingly, this also includes Src kinases. Consistent with the MS data, immunoblot analysis confirms that both the endogenous c-Src and exogenously expressed v-Src-ER fusion proteins increase in abundance (data not shown).

A similar analysis of the phosphatome ( Figure 14B), shows again that only a small subset of these enzymes change in abundance after v-Src activation. Specifically, we detect increased abundance of CDC25C, a protein involved in regulating the activity of the master cyclin-dependent kinase CDK1 and increased abundances of the dual-specificity tyrosine and serine/threonine protein phosphatases, DUSP1 and DUSP4. Furthermore, preliminary analysis of changes in phospho-peptide levels following v-Src activation, are consistent with these changes in protein phosphatase abundance levels altering cell signalling pathways. For example, we detect a clear decrease in the levels of phosphorylation at residues T185 and Y187 on the protein ERK2 ( Figure 14C). Both of these sites are known to be dephosphorylated by DUSP1 & DUSP4 (as reviewed in ( Caunt & Keyse, 2013)), whose abundances increase after Src-induced cell transformation ( Figure 14B). A more detailed, global analysis of the effect of v-Src activation on the phospho-proteome and signalling pathways will be reported separately.

### Role of Polycomb complexes in cell transformation

Previously, we showed that the increased motility phenotypes seen after v-Src activation are mediated, at least in part, by decreased abundance of the chromatin assembly factor 1 (CAF1) subunits ( Endo *et al.*, 2017). Furthermore, siRNA-mediated depletion of CAF1 subunits could increase cell motility and invasiveness in the absence of v-Src activation. Therefore, we examined whether v-Src activation caused any effects on epigenetic pathways and/or epigenetic factors known to be involved in reprogramming cellular phenotypes. For this, protein expression ratios for cells at the 72 hr time point -/+ v-Src activation were ranked according to p-value and filtered for relevant UniProt keywords, e.g. “epigenetic” and “chromatin” ( Supplementary Table 6). This revealed decreases in the abundances of the histone lysine demethylase PHF8, MCM proteins and the polycomb repressive complex 1 (PRC1) subunits, PHC3 and CBX6.

**Figure 15.**
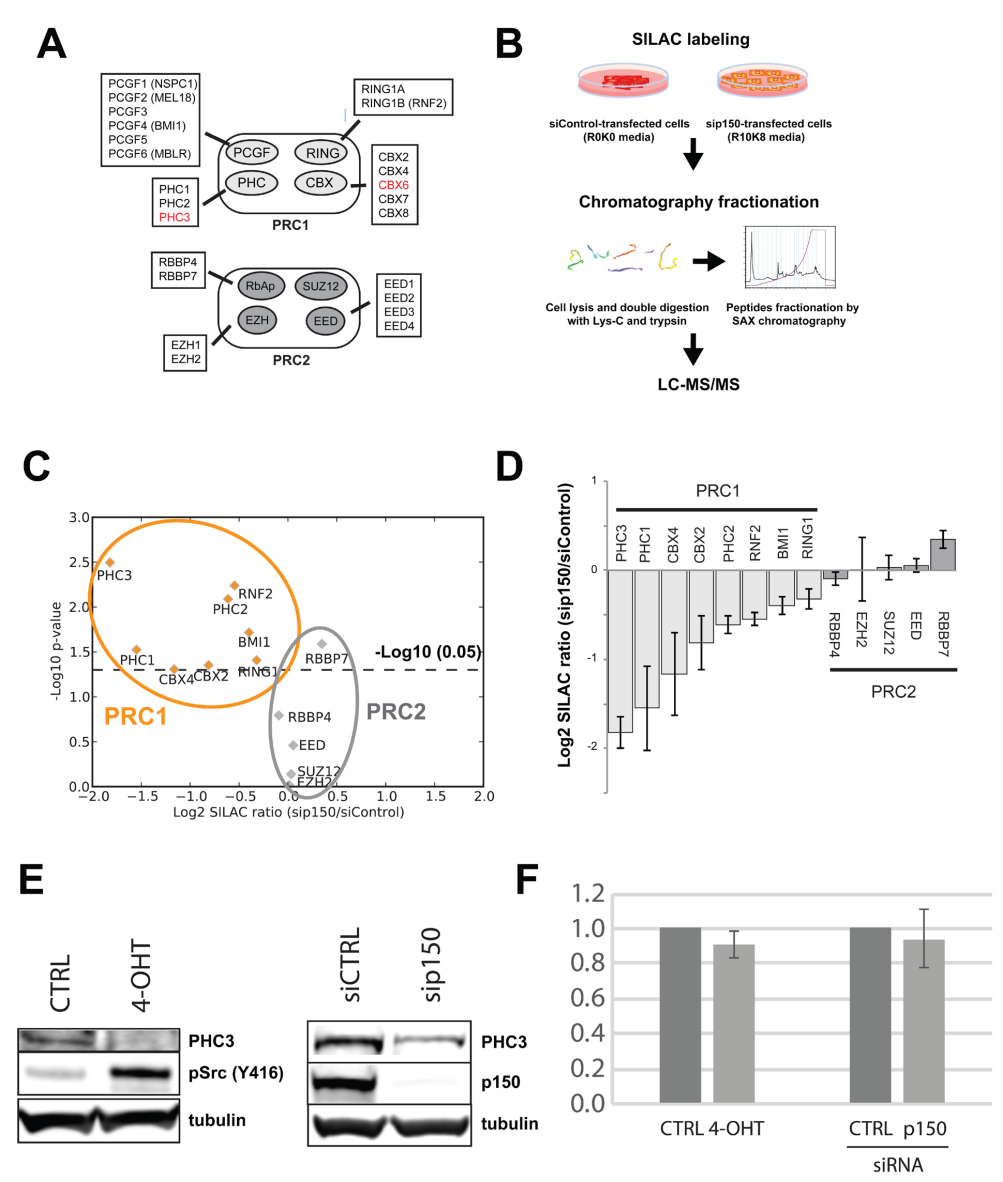
Src activation and CAF1-depletion both decrease levels of PRC1 subunits. ( **A**) Diagram of the polycomb repressive complexes (PRC1 & PRC2) and their subunits. Subunits in red show significantly decreased abundance after v-Src activation. ( **B**) Experimental diagram of a SILAC experiment to measure proteome differences between cells treated with non-targeting siRNA (siControl) versus cells treated with siRNA targeting CHAF1A/p150, a critical component of the CAF1 complex. ( **C**) Plot of –log _10_ p-value versus fold change shows downregulation of all PRC1 subunits measured. In contrast, PRC2 subunits remain either unchanged, or slightly increased. ( **D**) Bar chart showing fold change data from ( **C**). PRC1 and PRC2 subunits are shaded light and dark grey, respectively. ( **E**) Immunoblot analysis of PHC3 either (left) comparing -/+ Src induction conditions, or (right) comparing siCTRL versus siRNA targeting CAF1 p150 subunit (sip150). **F**) qPCR analysis of PHC3 mRNA, comparing same conditions as ( **E**).

Only two of the total PRC1 and PRC2 subunits identified, i.e., PHC3 and CBX6, change in abundance by two-fold or more, as summarized in Figure 15A. While smaller abundance decreases in other PRC1 subunits are also observed, all of the PRC2 subunits either show small increases in abundance, or do not change.

We next examined the effect on polycomb complexes of CAF1 depletion, in the absence of v-Src activation. To do this, we carried out a SILAC proteomic screen ( Figure 15, B–E, see Methods), comparing MCF10A Src-ER cells depleted of CAF1 subunits by siRNA, with control cells treated with a non-targeting siRNA. As observed with Src induction, CAF1 depletion also resulted in a decrease in the levels of several PRC1 subunits ( Figure 15, C–D). In contrast, no significant change in abundance was observed for PRC2 subunits ( Figure 15, C–D). Downregulation of PHC3 by either Src induction, or CAF1 depletion, was also observed by immunoblot analysis ( Figure 15E). Downregulation of PHC3 protein levels appears to occur via a post-transcriptional mechanism(s), because there is no parallel decrease detected in PHC3 mRNA levels, either following CAF1 knock-down, or after v-Src-induced cell transformation ( Figure 15F).

In summary, either CAF1-depletion, or v-Src-induced cell transformation, both resulted in a similar, reproducible decrease in the protein levels of the PRC1 subunit PHC3, compared to control cells, without a corresponding change in the levels of PRC1 mRNA.

### PHC3 is a suppressor of cell motility

**Figure 16.**
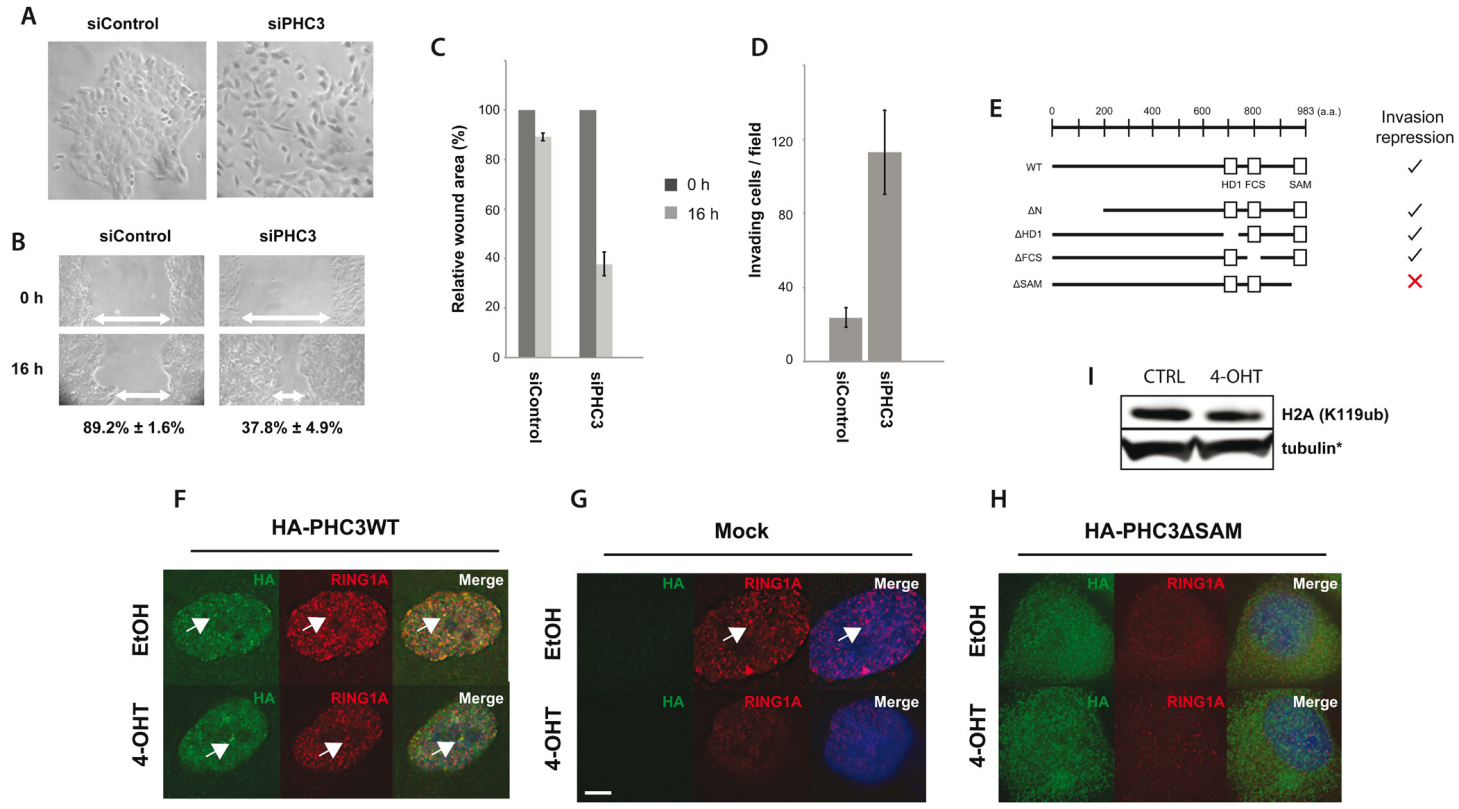
PHC3 depletion alters RING1A localization, cellular motility and invasion. Cells treated with either non-targeting siRNA (siControl), or siRNA targeting PHC3, were analysed by light microscopy for ( **A**) morphological changes, ( **B**, **C**) wound healing assays, and ( **D**) transwell Matrigel invasion assays. Error bars indicate standard error between three biological replicates. **E**) A summary of data comparing the ability of full length PHC3 and PHC3 truncation mutants to repress the invasion phenotype induced by v-Src activation. ( **F**, **G**, **H**) Cells were transduced with lentivirus encoding for either ( **F**) HA-PHC3WT (full length, wild-type), empty ( **G**), or ( **H**) HA-PHC3ΔSAM (SAM domain deletion mutant). Cells were then treated either with ethanol (control), or 4-OHT, and immunostained for HA tag (green) and RING1A (red). Arrows indicate RING1A nuclear foci. Representative images of three replicates. Scale bar: 5 µm. ( **I**) Immunoblot analysis of H2AK119ub -/+ Src activation. Note that the loading control shown is identical to Figure 15E.

We next tested the functional consequences of PHC3 downregulation on cellular phenotypes associated with oncogenic transformation and metastasis induced by activation of v-Src kinase, including altered cell morphology, wound healing and invasion. To do this, we compared MCF10A Src-ER cells depleted of PHC3 protein by siRNA, with control cells treated with a non-targeting siRNA, which retained unaltered levels of PHC3 ( Figure 16, A–D). The PHC3-depleted cells exhibited a ‘cell scattering phenotype’ ( Ridley *et al.*, 1995), which is characterized by loss of cell-cell adhesion, a more homogeneous distribution of cells across the 2D tissue culture dish and increased motility, which was not seen in the control cells ( Figure 16A). Consistent with reduced PHC3 levels promoting an increase in cell motility, the relative wound area remaining after 16 hrs was lower for PHC3-depleted (~40%), versus control cells (~90%), ( Figure 16, B & C). PHC3 depletion also led to increased numbers of cells positive for invasion (n = ~120), compared to the control cells treated with non-targeting siRNA (n = ~30), as measured in a Matrigel-coated transwell migration assay ( Figure 16D).

The PHC3 depletion data indicate that basal levels of PHC3 protein are important for suppressing cell motility in untransformed cells. We next explored this further by testing how PHC3 protein domains and expression levels influence the phenotypes mediated by activation of Src kinase activity. To do this, we analysed the effect of exogenous expression, from lentiviral vectors, of either wild-type HA-tagged PHC3, or various HA-tagged PHC3 truncation mutants, in cells -/+ Src activation ( Figure 16E). This showed that the number of cells positive for invasion, following Src activation, was significantly reduced in cells expressing wild type HA-PHC3, as compared with cells transduced with an empty HA vector control ( Figure 16E and Figure Supplement 1 – (Figure 16)).

These expression data using HA-tagged, wild type PHC3 support the conclusion that PHC3 suppresses the increased cell invasion caused by activation of v-Src kinase. A comparison of the ability of transduced HA-tagged PHC3 truncation mutants to suppress Src-mediated, increased cell invasiveness, indicates that this requires the carboxy-terminal sterile alpha motif (SAM) domain in PHC3. In contrast, either a deletion of the amino terminal domain, or several short internal PHC3 deletions, each still show suppression of motility ( Figure 16E).

The SAM domain of PHC3 is thought to be important for homo-oligomerisation and transcriptional repression ( Frey *et al.*, 2016; Robinson *et al.*, 2012). Fluorescence microscopy analysis, immunostaining for the HA epitope in wild type HA-PHC3, reveals the expected pattern of prominent punctate nuclear foci, i.e. ‘polycomb bodies’ ( Figure 16F, arrows). This staining is specific, because no signal is detected with the anti-HA antibody in the mock-transduced control cells ( Figure 16G). In contrast, immunostaining for HA-PHC3ΔSAM shows that this mutant fails to concentrate in the nucleus, does not form a similar pattern of foci to wild type PHC3 and instead produces granular staining throughout the cell ( Figure 16H).

We conclude that the SAM domain is critical for the function and nuclear organization of the wild type PHC3 protein.

Given the known role of PHC3 in formation of PRC1 complexes and the importance of the PHC3 SAM domain in forming protein-protein interactions, we next investigated the immunostaining patterns of other PRC1 subunits in cells transduced with either wild type, or mutant, HA-tagged PHC3 ( Figure 16F). RING1A, a PRC1 subunit with E3 ligase activity, predominantly colocalises in a similar punctate staining pattern to wild type PHC3 ( Figure 16F; white arrows indicate co-localisation of RING1A and wild type HA-PHC3 proteins in nuclear foci).

Following activation of v-Src kinase activity by treatment of cells with 4-OHT, both the levels of PHC3 seen by protein blotting ( Figure 15E) and the intensity of RING1A foci seen by immunostaining ( Figure 16F, compare upper and lower panels), decreases. A similar decrease in the intensity of RING1A foci seen by immunostaining is evident in the mock-transduced, control cells following 4-OHT treatment ( Figure 16G, compare upper and lower left panels). Further, there is a striking disruption in the pattern of RING1A localization in cells expressing the HA-PHC3ΔSAM mutant protein, independent of v-Src kinase activation ( Figure 16H, right panels). These data suggest that the HA-PHC3ΔSAM mutant may act as a dominant negative, reducing the ability of endogenous WT PHC3 to suppress invasive phenotypes. Indeed, expression of the HA-PHC3ΔSAM mutant construct led to an increase in invasion compared to mock transduced control ( Figure 16 FS1B).

We conclude that the SAM domain of PHC3 is important for the correct nuclear localization of both PHC3 and RING1A proteins.

To assess whether PRC1 E3 ligase activity is reduced after v-Src activation, we probed lysates for H2AK119ub. No significant change in the total H2AK119ub signal was observed after v-Src activation ( Figure 16I). In contrast, as a positive control, siRNA co-depletion of both RING1A & RING1B proteins was seen to result in a significant reduction in the H2AK119ub signal (data not shown). PHC3 is thus not required to maintain overall H2AK119ub levels, at least not at the majority of loci ( Figure 16F).

### Effect of v-Src Activation on Protein Turnover (Exp B)

In Exp B we evaluated the effects of Src-mediated cell transformation on the rates of protein synthesis, degradation and turnover, using the same pulse-SILAC method described above for untransformed cells (cf. Figure 2 & Figure 5). Measurements were made in cells that had been transformed by v-Src kinase activation for 48 hr, before starting the heavy isotope amino acid pulse (see Methods). This time point was chosen to coincide with the transformed cells reaching apparent steady state in global protein abundance changes and altered morphologies (cf. Figure 9).

Pulse-SILAC measurements of protein turnover (i.e. Exp B, both basal and transformed cell states; see Methods), were merged and filtered to include only data meeting the following stringent criteria: (i) tau (cf. Figure 5), measured in both conditions must not exceed the duration of the pulse-SILAC experiment (72 hr), (ii) the errors for tau must be less than 12 hr and (iii) the offsets (cf. Figure 5) must be greater than zero. The resulting data table ( Supplementary Table 7) comprises 8,412 proteins, along with their measured half-lives under both basal and transformed conditions and the corresponding time course data.

**Figure 17.**
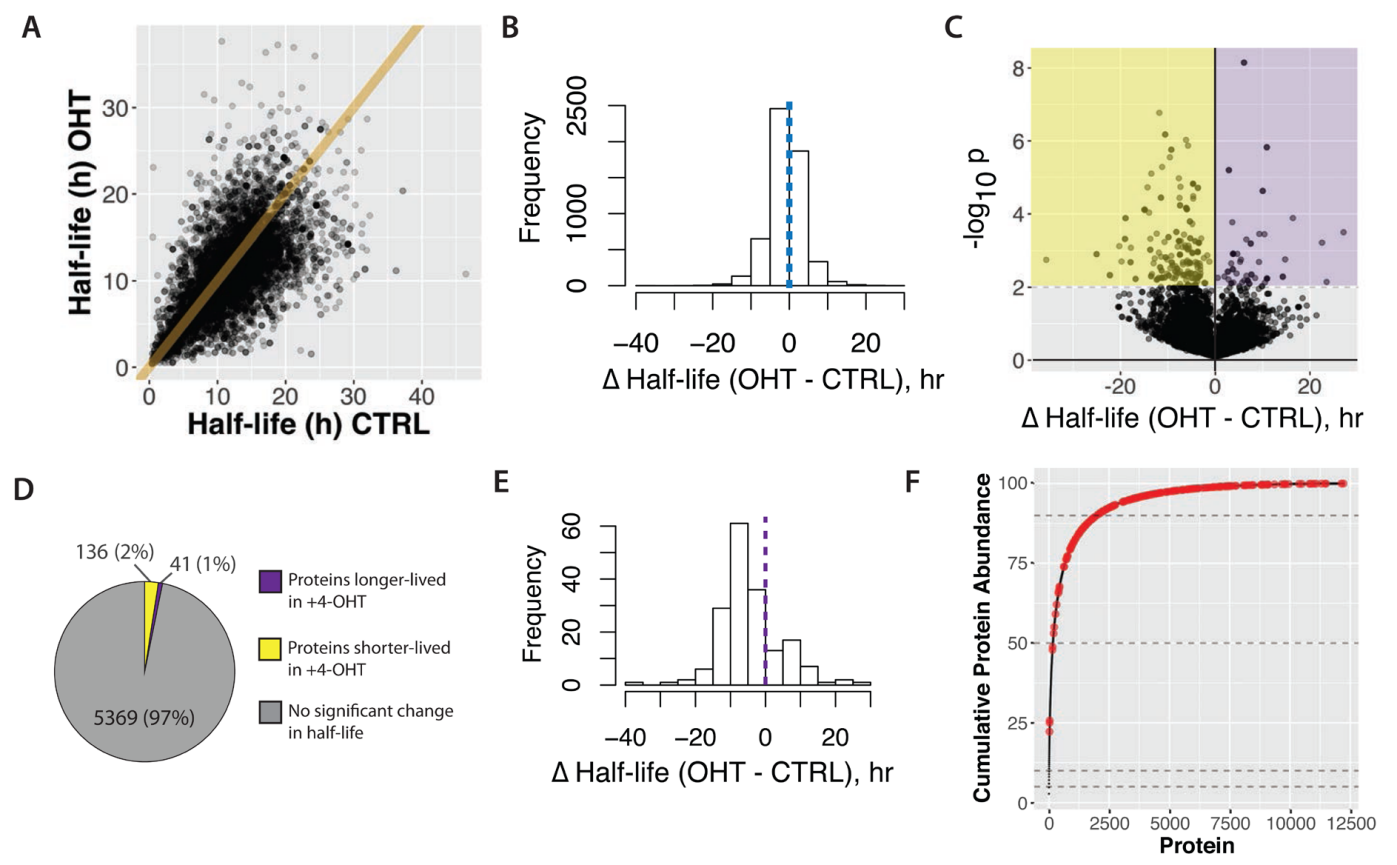
Comparison of half-lives in basal (CTRL) versus transformed (4-OHT) cells. **A**) Scatterplot of half-lives in 4-OHT- versus control-treated cells. The equation of the line shown is y = x. **B**) A histogram of half-life difference between 4-OHT- and control-treated cells shown for all proteins. The dotted line in the centre indicates 0, i.e. no difference between the two conditions. **C**) Volcano plot of –log _10_(p-value) versus difference in half-life. Yellow and purple shading demarcates cut-offs for proteins either significantly shorter-lived, or longer-lived, respectively, in 4-OHT-treated cells. **D**) Proportion of proteins that significantly change half-life. **E**) Histogram of half-life difference between 4-OHT- and control-treated cells shown for proteins that significantly change half-life. The dotted line in the centre indicates 0, i.e. no difference between the two conditions. **F**) Cumulative abundance plot (cf. Figure 4A) with proteins that significantly change half-life highlighted in red.

From the data presented in Supplementary Table 7, the (unweighted) median protein half-life in transformed cells is 10.9 hr, compared with 11.6 hr for untransformed, control cells (p < 0.001, t-test). Figure 17A shows a scatter plot, comparing protein half-lives measured in control (CTRL), versus transformed (4-OHT), cells. A line of best fit from linear regression, calculated across the entire data set, has a slope of 0.86 (r ^2^ = 0.88). The bias towards decreased half-lives in 4-OHT treated cells is more easily observed in the histogram of half-life differences shown in Figure 17B, consistent with our finding that the *unweighted*, median protein half-life is reduced in transformed cells.

We also calculated the *abundance*- *weighted*, median protein half-life in transformed cells, which was 12.7 hrs. This increase in median half-life of ~17% in transformed cells, when abundance weighting is accounted for, is similar to the abundance weighted increase of ~20% measured in untransformed cells (cf. Figure 6). Thus, both the weighted and unweighted median half-life calculations show that cell transformation results in a proteome-wide reduction of average protein half-lives. The weighted median half-life decreased by ~1.5 hrs, consistent with cell transformation causing an increase in protein turnover.

Regression analysis was also performed on a subset of 4,954 proteins, selected for having the highest quality data, as defined by an exponential fit r ^2^ > 0.95 under both the control and transformed cell conditions. This results in a slope of 0.88 (linear regression r ^2^ = 0.91; data not shown). We conclude that the decrease in median half-life induced by transformation is robust to differences in exponential fit quality.

In summary, we conclude there is a robust reduction of ~1.5 hr in the average protein half-life in cells transformed by v-Src kinase activation.

### Proteins showing altered half-life in transformed cells

Next, we assessed how the half-lives of individual proteins were affected by cell transformation induced by v-Src activation. To do this, Z-scores were calculated based on both the difference in half-life between control and transformed cells and the errors determined for individual half-life measurements. These data were visualized in a volcano plot, comparing p-value versus half-life difference ( Figure 17C). Selecting an arbitrary p-value cut-off value of 0.01, 177 proteins showed a significantly changed half-life, with >75% having a lower half-life in the transformed cells ( Figure 17D).

Figure 17E shows Δt _1/2_ in a histogram, illustrating the distribution of protein half-life values for the proteins significantly changing half-life after transformation. Interestingly, the majority of altered proteins (136/177), decrease half-life after transformation, with an average reduction of ~8.3 hrs. Most of these proteins are expressed at medium to low abundance ( Figure 17F).

**Figure 18.**
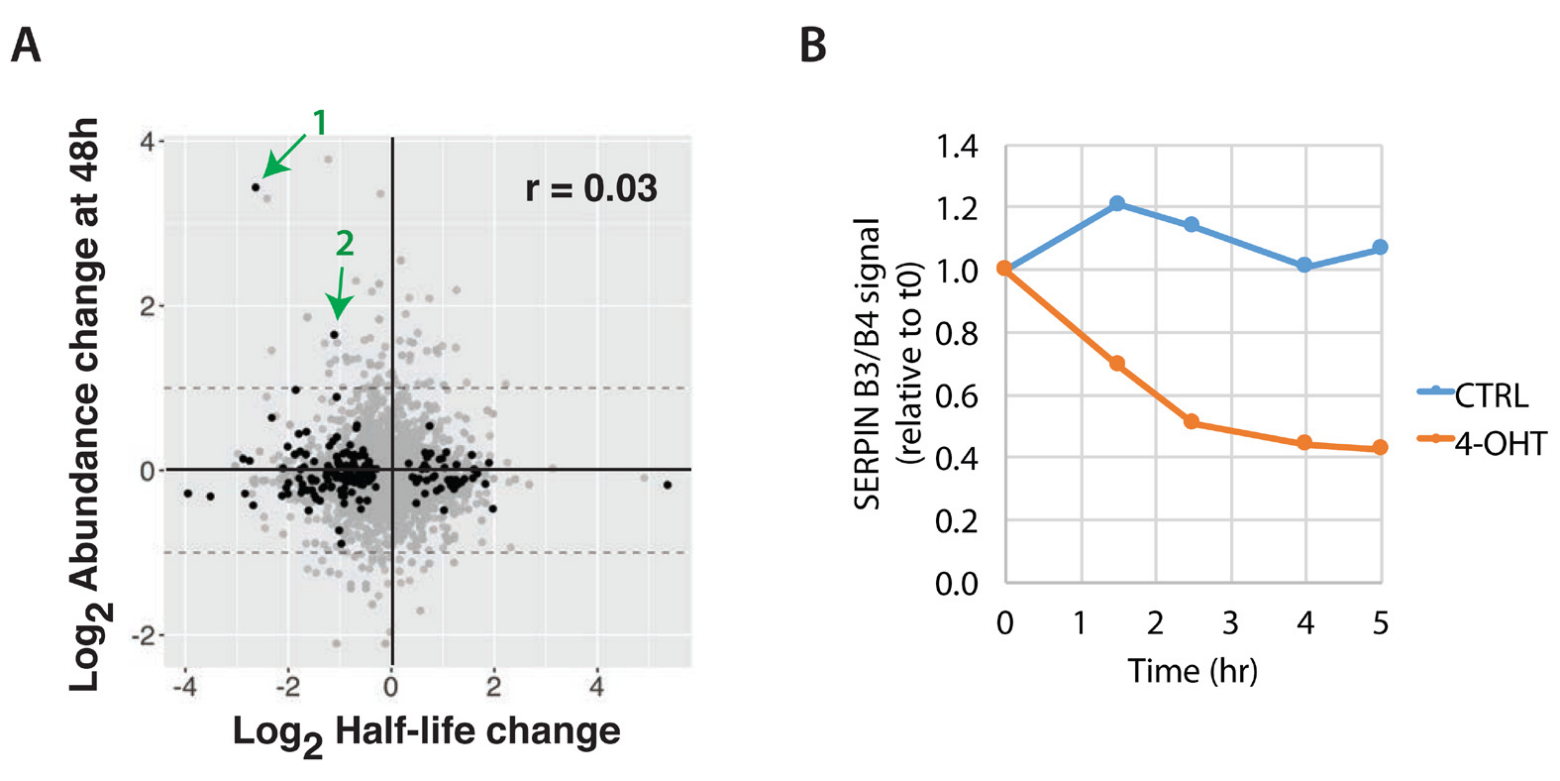
Cross-correlation of protein abundance and half-life changes. **A**) Comparison of log _2_ protein abundance ratio (4-OHT/CTRL) at 48 hr and log _2_ half-life ratio (4-OHT/CTRL). Black points indicate p < 0.01. Green arrows indicate two proteins, SERPIN3&B4 ( **1**) and SERPINB1( **2**). The Pearson correlation (r) is 0.03. **B**) Densitometric quantitation of the immunoblot images obtained from analysis of cells that were treated with emetine (inhibitor of translation) for the indicated time points, harvested, and immunoblotted for beta tubulin and SERPINB3 & B4.

We next analysed further the subset of 177 proteins showing altered half-life in v-Src transformed cells, to examine whether changes in half-life correlated with protein abundance changes between CTRL and 4-OHT treated cells at 48 h ( Figure 18A). We note these proteins include several members of the serpin family, including SERPINB3/B4 and SERPINB1 (cf. Figure 11E). Interestingly, despite showing a decreased half-life, indicating a higher turnover rate, these serpin proteins actually increased in total abundance after v-Src activation. In particular, the abundance of SERPINB3/B4 rapidly increased by ~10-fold within 48 hr of activating v-Src, as compared with control cells ( Figure 11E), while the half-life of SERPINB3/B4 decreases from ~13 hrs to ~2 hrs (see Supplementary Table 7).

The dramatic increase in SERPINB3/B4 levels was independently confirmed by protein blotting analysis of cell extracts, 24 hr after v-Src activation ( Figure 11F). Furthermore, consistent with the pulse SILAC data, immunoblot analysis of cells treated with emetine (a small molecule inhibitor of protein translation) independently confirmed the significant decrease in SERPINB3/B4 protein stability in transformed cells, with an estimated half-life of ~2 hr under transformed conditions ( Figure 18B). One explanation for these unexpected results is that the markedly reduced half-life of SERPINB3/B4 could reflect the transformed cells attempting to reduce the increased levels of SERPINB3/B4 back to the normal abundance seen in control cells.

### Src proteomic signature is prognostic of poor clinical outcome

Next, we evaluated whether the proteomic changes observed here in the Src-ER cellular model would be reflected in cancer patient outcomes in the clinic. To do this we created a protein-level ‘Src signature’, using the high stringency clustering data (cf. Figure 11). The signature comprises in total 248 proteins, which each change in abundance by at least 2-fold and for which we have data across all seven time points analysed after activation of v-Src kinase ( Supplementary Table 8).

We sought to compare this proteomic Src signature with gene expression changes in patient tumours measured in the TCGA data set ( Cancer Genome Atlas Research Network *et al.*, 2013). As described above, we had selected the Src-ER model for this proteomic study after finding a clear positive correlation between increased SFK activity and poor clinical outcome in the TCGA protein array (RPPA) data set (cf. Figure 1). Unfortunately, because most of the proteins we detect in the Src signature were low abundance and not measured in the TCGA RPPA dataset ( Akbani *et al.*, 2014), we could not make a direct comparison of protein level differences with patient outcomes. However, because extensive mRNA characterization has been performed on the TCGA samples, we therefore resorted to comparing our Src signature proteomic data with the TCGA mRNA data. This is justified by previous studies showing that bulk protein and mRNA abundances are moderately positively correlated, ( Lundberg *et al.*, 2010; Ly *et al.*, 2014; Schwanhausser *et al.*, 2011), although we note that the accuracy of this relationship can vary significantly on a per-gene basis.

**Figure 19.**
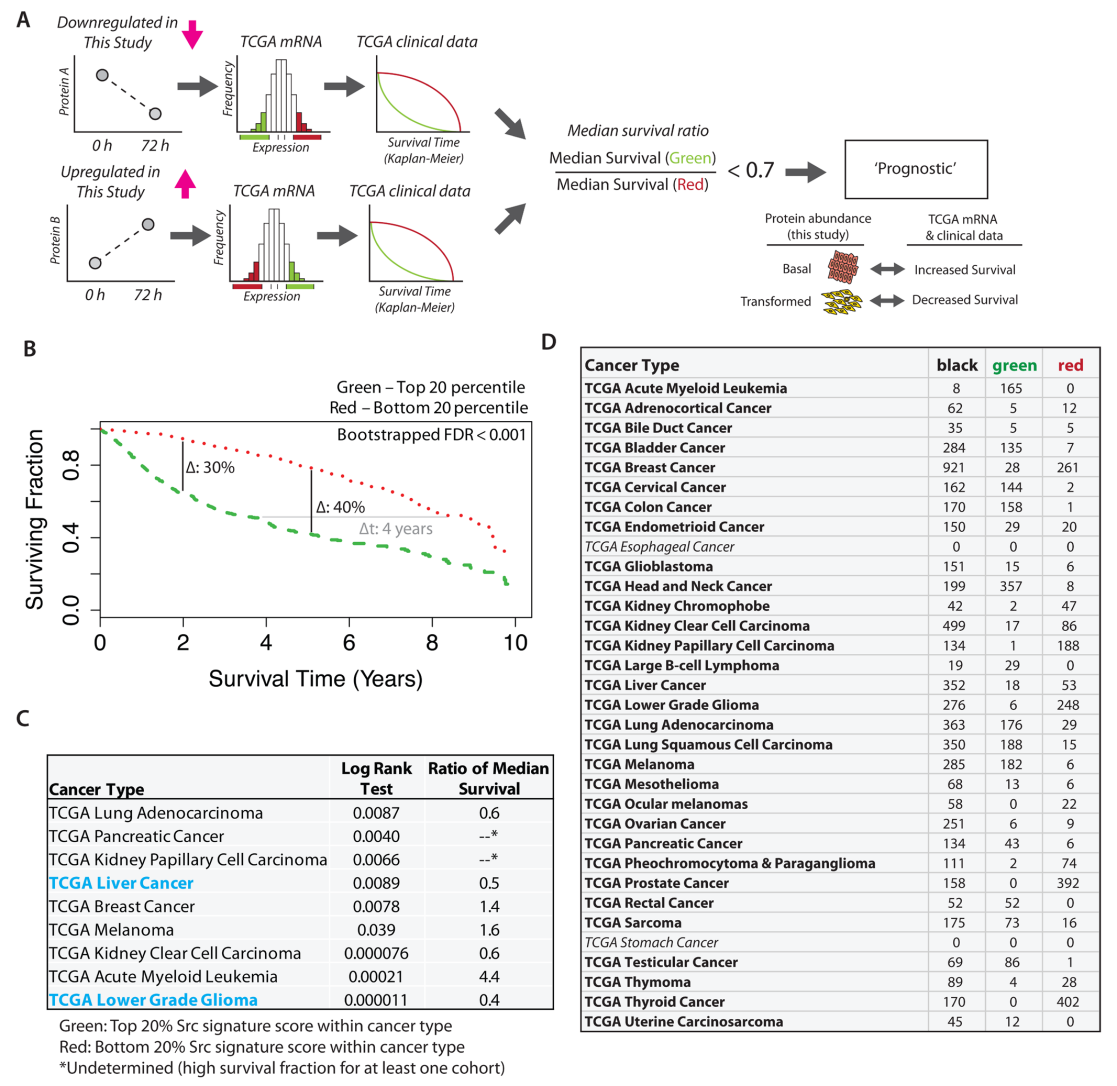
Src proteomic signature is a predictor of poor clinical outcome across distinct cancer types. **A**) Scheme illustrating the concept of the Src signature score. **B**) TCGA patients were stratified into five cohorts based on mRNA intensities for proteins in the Src signature. Survival curves for patients showing the top (green dashed line) and bottom quantile (red dotted line) quantile Src signature are plotted. Log rank test p < 1 × 10 ^-10^. A bootstrapping method was used to calculate FDR for this effect size (~4 years difference in median survival). **C**) Summary of Src signature survival analysis within each cancer type. Cancer types showing significant log rank p-values (p < 0.05, n = 9) are shown. For two cancer types, ratios of median survival were not determined due to a high surviving fraction for at least one cohort. **D**) The number of samples showing top versus bottom quantile Src signature score grouped by cancer type. Italicised cancer types do not have mRNA data currently deposited.

Therefore, using TCGA mRNA measurements as proxies for protein level changes, patient gene expression profiles were scored based on conversion of our Src proteome signature to a corresponding Src gene signature ( Figure 19A). Briefly, expression values from Src signature genes were linearly combined into a ‘Src signature score’, with coefficients (+1 or -1) corresponding to whether the encoded protein was either increasing, or decreasing, in our proteomics data set. Patient cohorts were identified based on the highest and lowest score bins (i.e., top 20 ^th^ vs. bottom 20 ^th^ percentiles). We then evaluated whether there was a significant difference in survival time between patients in the respective cohorts.

Figure 19B shows the resulting KM survival curves plotted for these two cohorts. The green and red dashed lines represent patients that have high and low Src signature scores, respectively. These data show a strong negative correlation between the Src signature score and length of patient survival post tumour diagnosis ( Figure 19B). The median survival difference between the two cohorts is ~4 years. At 5 years post tumour diagnosis, there is a difference between the cohorts of ~40% in the fraction of surviving patients (vertical line).

As a control for this analysis, a permutation-based, bootstrapping algorithm was used to estimate the false discovery rate (FDR). For this we compared 1,000 randomly generated protein signatures, all of equal length to the Src signature. None of the 1,000 random permutations either matched, or exceeded, the experimental result shown from our Src signature data ( Figure 19B).

In summary, we conclude that the major decrease observed in average survival time post tumour diagnosis, between patient cohorts with highest and lowest matches to our Src signature, is highly significant and unlikely to occur by chance (FDR < 0.001).

### Cancer subtypes

The analysis above ranks patient survival for correlation with the Src proteomic signature across all cancer subtypes in the TCGA data set. We next asked whether ranking within cancer subtypes would show differences in clinical outcome. Figure 19C lists the cancer subtypes that show a significant (log rank p < 0.05) difference in length of survival between patient cohorts with the respective highest and lowest Src signature scores within that subtype. A breakdown of patient cohort membership by cancer type is shown in Figure 19D. Interestingly, these data show clear differences in length of survival for different cancer subtypes, albeit with significant variation in both patient numbers and therapeutic histories between the subtypes. Nonetheless, the data show that the Src signature score is prognostic for poor patient outcome within the TCGA data set, with potentially higher predictive power for certain cancer subtypes, notably including liver cancer and lower grade glioma.

### Relation of Src proteomic signature to mRNA signatures

As explained above, to compare our experimentally determined protein-level Src response signature with patient outcomes, we had to convert the protein data into a corresponding transcript signature. It is likely, therefore, that the true prognostic value of our proteomic Src signature is underestimated, because for any of the proteins where a change in their abundance in response to Src activation is controlled by post-transcriptional mechanisms (e.g. change in rates of translation, degradation, and/or secretion), this may not be reflected in a corresponding change in mRNA level and therefore not reported by either a transcriptomics, or microarray assay. For example, we showed that the PRC1 complex subunit PHC3 decreases in abundance at the protein level after Src activation, without a detectable change in its mRNA level ( Figure 15F).

**Figure 20.**
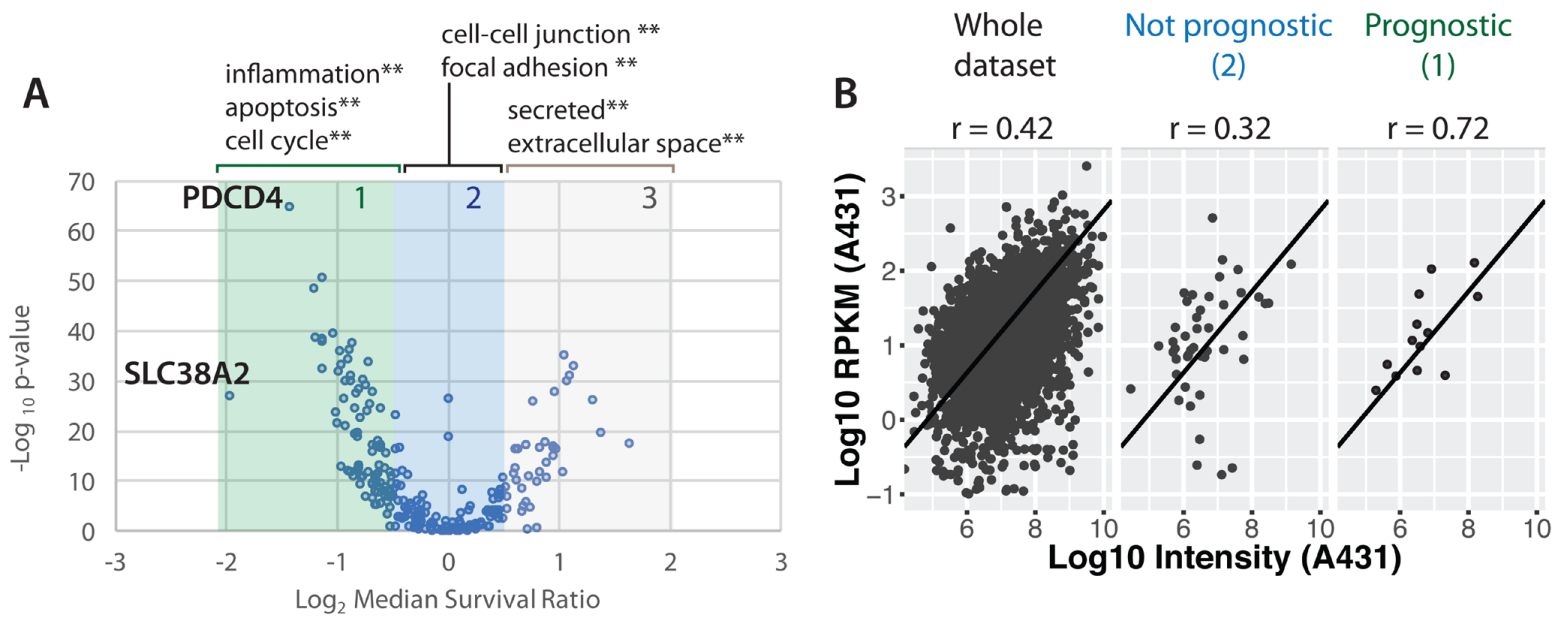
Prognostic markers are more likely to have correlated protein and mRNA levels. **A**) Plot of –log10 p-value from log rank test for individual gene in predicting patient survival. Shaded boxes indicate three classes (‘class 1’, ‘class 2’, ‘class 3’) of ‘prognostic’ versus ‘non-prognostic’ genes. Enriched gene ontology terms for each group are shown above the scatter plot. Asterisks indicate enrichment p-value of less than 0.001. **B**) A scatter plot showing the mRNA and protein levels measured in the epithelial carcinoma cell line A431 ( Lundberg *et al.*, 2010) for all proteins detected in our MCF10A Src-ER data set (left) and proteins that we have deemed ‘not prognostic’ (‘class 2’, middle) and ‘prognostic’ (‘class 1’, right).

We therefore addressed the potential impact of post-transcriptional and/or post-translational regulation of protein abundances within the Src signature, with respect to its ability to predict patient outcomes when used in conjunction with transcript data. To do this, we analysed how well different protein abundances from our Src response data set compare with RNA abundances in a previously published transcriptome study ( Lundberg *et al.*, 2010). As shown in Figure 20, the proteins that provide the highest prognostic value in comparisons between patient survival and transcriptome data are also the proteins that show the strongest positive correlation between corresponding mRNA and protein abundance levels in epithelial adenocarcinoma cells (Spearman’s r ^2^ = 0.72, p-value = 0.002, Figure 20B, right panel). This finding is consistent with our prediction that the prognostic value of the Src proteomic signature is currently reduced, because a subset of the proteins do not show corresponding changes at the mRNA level.

### Src signature stratifies cell lines resistant to Src inhibitors

**Figure 21.**
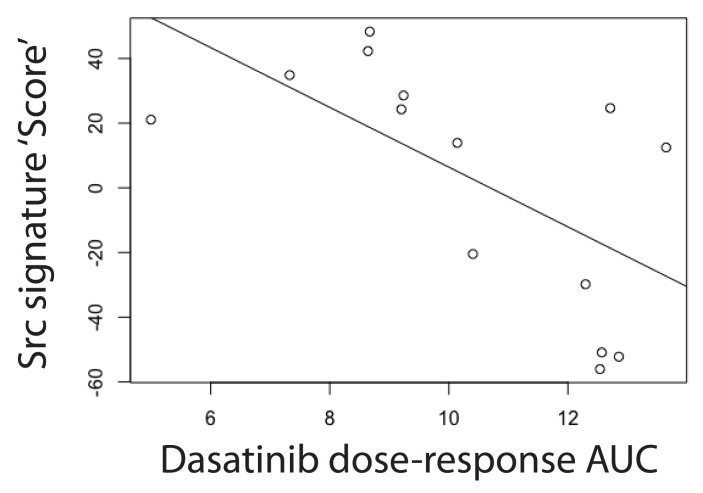
Src signature scores and dasatinib sensitivity. A comparison of Src signature scores calculated from triple-negative breast cancer cell lines ( Lawrence *et al.*, 2015) and dasatinib sensitivity from the CCLE database ( Seashore-Ludlow *et al.*, 2015).

While there is currently a dearth of detailed proteome measurements linked with patient records in the public domain, there are more proteomic datasets available from analysis of human cell lines. Therefore, we next performed a preliminary analysis to test whether our proteomic Src signature could also be used to stratify human cell lines by predicting whether they are either responsive, or resistant, to clinically-relevant Src kinase inhibitors. To do this, we used the Src signature data to reanalyze two previously published data sets. First, a quantitative, MS-based analysis of the proteomes of a panel of human triple negative breast cancer (TNBC) cell lines ( Lawrence *et al.*, 2015). Second, data from CCLE showing the sensitivity of these same human cell lines to the inhibitor Dasatinib ( Seashore-Ludlow *et al.*, 2015), which inhibits Src family kinases (on-target) and also ephrin receptors (off-target) ( Creedon & Brunton, 2012). As shown in Figure 21, Src signature scores calculated using proteome-wide protein abundance data for TNBC cell lines showed a statistically significant correlation with Dasatinib sensitivity (r ^2^ = 0.40, p-value, 0.016, Figure 21). This preliminary analysis, involving a three-way comparison of disparate data sets, each with distinct sources of variability and technical error, has obvious limitations in its sensitivity. Nonetheless, it is striking that a significant level of stratification for Dasatinib sensitivity between these cell lines was obtained by focusing analysis on the expression levels of the proteins within the Src signature.

In summary, the overall results above using the experimentally determined Src signature are consistent with our overarching hypothesis that proteomic response data measured using this epithelial cell model can identify important pathways downstream of SFK activity that contribute to cell phenotypes associated with oncogenic transformation.

### Data visualization through the Encyclopedia of Proteome Dynamics

**Figure 22.**
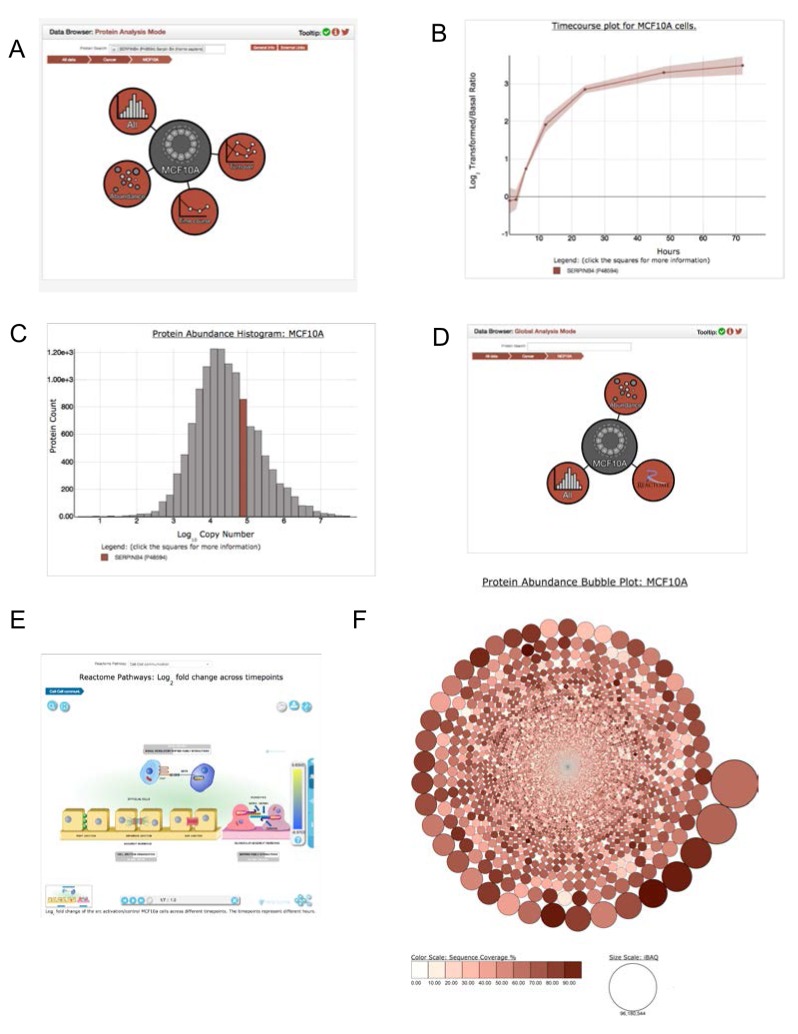
The Encyclopedia of Proteome Dynamics. **A**) Homepage in EPD for the ‘Protein Analysis Mode’ of the interactive viewer, showing data types available. The page includes a dialogue box for searching for either proteins/gene names, GO terms, or protein complexes. Example plots of ( **B**) time-course kinetics and ( **C**) abundance histogram, for SERPINB3 & B4. ( **D**) Homepage for the EPD ‘Global Analysis Mode’, enabling researchers to view the dataset mapped to ( **E**) Reactome pathways and ( **F**) a bubble plot of protein copy numbers. Protein identities show up as tool tips when moused over.

To increase the value of the comprehensive proteomic data presented in this study, we have incorporated all the data into our open access, searchable online database, the Encyclopedia of Proteome Dynamics ( Brenes *et al.*, 2017) (EPD; www.peptracker.com/epd). As illustrated in Figure 22, the EPD provides multiple interactive visualisations, allowing for convenient searching and interactive exploration of all the processed proteomics data. It also provides links to download both the processed data and associated raw MS files, the latter having been deposited in the ProteomeXchange PRIDE repository (accession PXD009270).

The data visualisations in the EPD can be explored by clicking on selected data points, which reveals tooltips providing additional information and links to other related online data resources, including Uniprot and STRING. An important feature in the tooltip box is the ability to ‘search in EPD’. Selecting this option filters all of the data within the EPD database and displays access specifically to all other data sets that include detection of the selected protein of interest. Further, using the accompanying Search Box displayed at the top of the user interface, data visualizations can be searched using multiple selectable criteria, including either proteins/genes of interest, GO terms, or subunits of specific protein complexes ( Figure 22A, D). Identified components can then be readily highlighted on the displayed plot and the resulting annotated visualization also downloaded by the user and saved as a .svg file that can be edited further using external vector graphics software.

As an example of data exploration facilitated by the EPD, protein turnover plots, including calculated half-life values, can be displayed for any of the >9,000 proteins for which kinetic data were obtained from the pulse-SILAC analysis. For each of these proteins, the plot shows a protein synthesis and protein degradation curve, which can be separately toggled off and on. Similarly, the error for each curve, shown as a ribbon, can also be toggled off and on. Also, for each of the 8,412 proteins for which turnover data were obtained under both control and transformed conditions, the respective synthesis and degradation curves, under both conditions, can be displayed on the same plot and each curve toggled off/on, in any combination. Furthermore, by typing the name of a protein of interest in the protein search box at the top of the interface, kinetic data from additional proteins can be added to the plot and compared. The visualization also automatically creates a colour-coded legend at the bottom of the plot, with each element in the legend clickable to reveal a tooltip box with display options.

For more detailed descriptions of the functionality and use of the EPD, see ( Brenes *et al.*, 2017). We also highlight here new functionality for pathway analysis that has been added recently to the EPD and integrated with the Src transformation proteomics data set. Specifically, the EPD now provides pathway analysis via integration with Reactome ( https://reactome.org). As illustrated in Figure 22E, the Reactome module can be selected from the analysis options presented for the Src data set in the EPD user interface. Clicking on the disk labelled ‘Reactome’ provides access to a wide range of graphical displays, each showing different pathways and cell structures, which can be viewed with the cognate protein data from this study overlayed. Here, the protein data are colour-coded to represent abundance levels at each time point following activation of v-Src kinase activity.

In summary, via the combined EPD and PRIDE resources, open access to the entire proteomic data set presented in this study, from processed protein level abundance and kinetic data through to raw MS files, is provided in a uniquely convenient, searchable and interactive format.

## Discussion

In this study, we have performed an in-depth characterization of the proteome of untransformed, human breast epithelial cells. We also carried out a comprehensive ‘time-lapse proteomics’ and functional study on the remodelling of this proteome driven by activation of v-Src kinase, across a time course during which the cells undergo oncogenic transformation, showing profound phenotypic changes in morphology, motility and invasiveness. In addition, we performed the first global analysis to measure how rates of protein synthesis and protein turnover are affected by cell transformation. Our study of this epithelial cell model thus provides the most detailed picture reported to date of the downstream consequences, at the protein level, of cellular transformation induced by activation of any oncogene.

In addition to the unbiased identification of specific proteins affected by Src activation and analysis of their potential clinical significance ( *vide infra*), these data offer several general conclusions, at a proteome-wide level, regarding the effects of cell transformation. First, we conclude that Src-induced transformation significantly alters cell phenotypes while only changing the expression and/or turnover levels of a minor fraction (~3% or less) of the cell proteome. Taking abundance weighting into account, this corresponds to only ~1.5% of the total protein molecules in the cell. Second, we find that the predominant effect of transformation, i.e. for ~75% of Src-responsive genes, is to decrease protein expression and/or to increase protein turnover. However, there are notable exceptions where specific proteins also increase. Third, we show that transformation increases the average rate of proteome turnover. The data suggest transformation can disrupt mechanisms involved in protein homeostasis. Fourth, we identify distinct classes of protein kinetics in response to Src activation. We find that proteins showing similar kinetics share related cellular functions, which can be linked with the kinetics of changes in cell phenotypes. Importantly, fifth, we show that many of the Src-regulated proteins are present in low, to very low, abundance and some are regulated post-transcriptionally. Therefore, some important components of the cellular response to transformation may not have been detected in previous studies, particularly when exclusively RNA-based detection methods (e.g. microarray, RNA-seq etc.), were used to identify gene-expression changes in cancer.

To maximize the value of all these proteomic data, we provide open access to search and interactively explore all of this information via the online, EPD database ( www.peptracker.com/epd).

### Src signature & cancer

Our proteomic data define a ‘Src signature’, corresponding to a set of 248 proteins whose abundance significantly alters after activation of Src tyrosine kinase activity. The identities and functions of some of these signature proteins are discussed further below. The clinical relevance of the Src signature was shown by several observations. First, multiple genes already used in the clinic as cancer biomarkers encode proteins within the Src signature, including Her2, MUC16, PLAU, SERPINE1, Aurora A kinase, Cyclin B1, GRB7 and Ki-67. Second, the signature is highly prognostic of poor patient survival across multiple tumour types, with potentially strongest predictive power for certain cancer subtypes, including liver cancer and lower grade glioma (cf. Figure 20). Thus, in an analysis comparing our data with clinical data from TCGA, cancer patients showing gene expression changes with the closest match to the pattern of the Src signature, on average survive ~4 years less post diagnosis than the patients showing the lowest match.

Amongst the key drivers of poor cancer patient survival in the clinic are the specific changes in the behaviour of cancer cells that contribute to metastases, e.g. phenotypes causing increased cell motility and invasiveness. These phenotypes can arise through multiple different triggering events. Therefore, rather than focusing on the event that may initiate cell transformation, we focussed here instead on measuring the downstream, protein level consequences of transformation. Our hypothesis was that proteome remodelling induced by v-Src activation in cell culture could mirror, at least in part, some of the protein-level effects driving adverse phenotypic changes in cancer patients, even although these may be initiated *in vivo* by different oncogenes and mutational mechanisms. The fact that the proteomic ‘Src signature’ we identified is prognostic of poor patient survival across a range of cancer types supports this hypothesis.

As highlighted above, our proteome remodelling data show that multiple proteins, encoded by genes that are already in use in the clinic as tumour markers, alter their expression levels after the activation of Src kinase activity in this epithelial cell model. In addition, the data also identify new potential protein biomarkers, protein activities and cellular pathways that may be useful as future clinical markers and/or cancer drug targets. We note that since many of the Src-responsive proteins identified are expressed at very low abundance, and since some of these proteins appear to be regulated post-transcriptionally (e.g. PHC3), they may not have been detected in previous screening studies that either relied exclusively on transcriptomic measurements, or that used protein detection methods lacking the depth of our current MS-based proteomics analysis. For example, most of the proteins we identify here in the Src signature were not included in the previous TCGA protein array studies.

Proteogenomic efforts have recently begun to characterise the proteome variation ‘ *in situ*’ using cancer patient material. The challenging nature of analysing limited sample material in complex tissue environments means that these approaches currently have to trade depth of proteome analysis to enable an increase in the breadth of clinical samples that can be analysed. For example, recent analyses achieve an impressive proteomic depth of ~8,000 proteins, quantitatively measured in 77 breast cancer samples, with matched measurements of gene copy number alterations and mRNA abundances ( Mertins *et al.*, 2016). Interestingly, our Src signature includes proteins shown by Mertins *et al*. to be associated with specific breast cancer subtypes. These include GRB7, which was shown to be associated with ERBB2 copy number amplification, and the cell cycle/replication stress kinases Aurora A (AURA) and Chk1 (CHEK1), which were shown to be associated with basal-like subtype.

However, our analysis here on the MCF10A cell model of oncogenic transformation showed that the epithelial proteome extends to expression of ~14,000 proteins or more, with >7,000 of these proteins accounting for less than 5% of the total protein copies in the cell. This set of low abundance proteins includes >75% of the expressed kinases, along with many transcription factors and other functionally important proteins that are typically ‘missing’ from current clinical analyses. Additionally, we show here that many of the Src signature proteins responding to cell transformation belong to this group of low copy number factors in epithelial cells. Therefore, many of the signature proteins will be challenging to detect in unbiased, high-throughput studies, unless increased proteome depth is achieved. Our data suggest that to capture relevant disease phenotype-associated proteins (as identified in our Src signature), further technological development is still required to achieve high breadth of clinical samples without sacrificing the requisite proteomic depth needed to detect important protein biomarkers that respond to transformation.

We have characterized the downstream, protein-level effects of cell transformation resulting from activating Src kinase activity (cf. Figure 1). Previous reports have highlighted a correlation between elevated c-Src expression and cancer patient survival. For example, increased levels of c-Src mRNA were reported to correlate with poor clinical outcome in many tumour types, including colon, liver, lung, breast and the pancreas (reviewed in ( Irby & Yeatman, 2000)). However, an immunohistocytochemical analysis of breast cancer tissues, comparing antibodies recognizing either total SFK protein levels, or active SFKs (i.e., SFKs phosphorylated at Y416), indicated that it is specifically markers of Src activity that are highly correlated with breast tumour malignancy, while total levels of Src protein are not correlated ( Elsberger *et al.*, 2010). Our analyses in this study support the latter view that it is primarily the level of active Src, rather than total Src protein expression, which may be important in regulating molecular mechanisms involved in carcinogenesis and/or cancer progression. (cf. Figure 1).

SFKs were an early target for pharmacological development ( Levitzki & Gazit, 1995) and potential clinical intervention, with several small molecule Src kinase inhibitors now either approved, or in development, for clinical use ( Hennequin *et al.*, 2006). However, despite the strong links between elevated Src kinase levels and poor patient survival, the clinical benefit for patients treated with SFK inhibitors was so far disappointing ( Creedon & Brunton, 2012), particularly in patients presenting with solid tumours ( Fury *et al.*, 2011). One possible reason for this could be that cancer phenotypes that are triggered initially by increased Src kinase levels, subsequently become independent of continued Src activity. For example, a potential mechanism is provided by the observation that v-Src induction can trigger a positive, feed-forward loop, involving the let-7 microRNA and proteins involved in a pro-inflammatory response, e.g. NFkappaB, STAT3, and IL-6 ( Iliopoulos *et al.*, 2009). Additionally, based on our observations that Src activation regulates proteins affecting cell motility and invasiveness phenotypes in culture, it may be worth re-evaluating the clinical use of Src inhibitors to treat the development of metastases, rather than late stage solid tumours.

### Proteome remodelling & cell transformation

Our current data set provides a unique insight into both the identities of the specific proteins whose expression levels change following activation of Src kinase activity, along with the kinetics of their respective responses. As these data are also linked with the corresponding kinetics of change in transformed cell phenotypes, this provides important clues concerning potential molecular mechanisms and signalling pathways that may contribute to changing the behaviour and/or responses of the cells upon transformation. To facilitate hypothesis generation and further exploration of the relationships between altered protein expression and cancer cell phenotypes, we have integrated these time-lapse proteomics data with Reactome pathways and provided open access via the online EPD database ( www.peptracker.com/epd).

**Figure 23.**
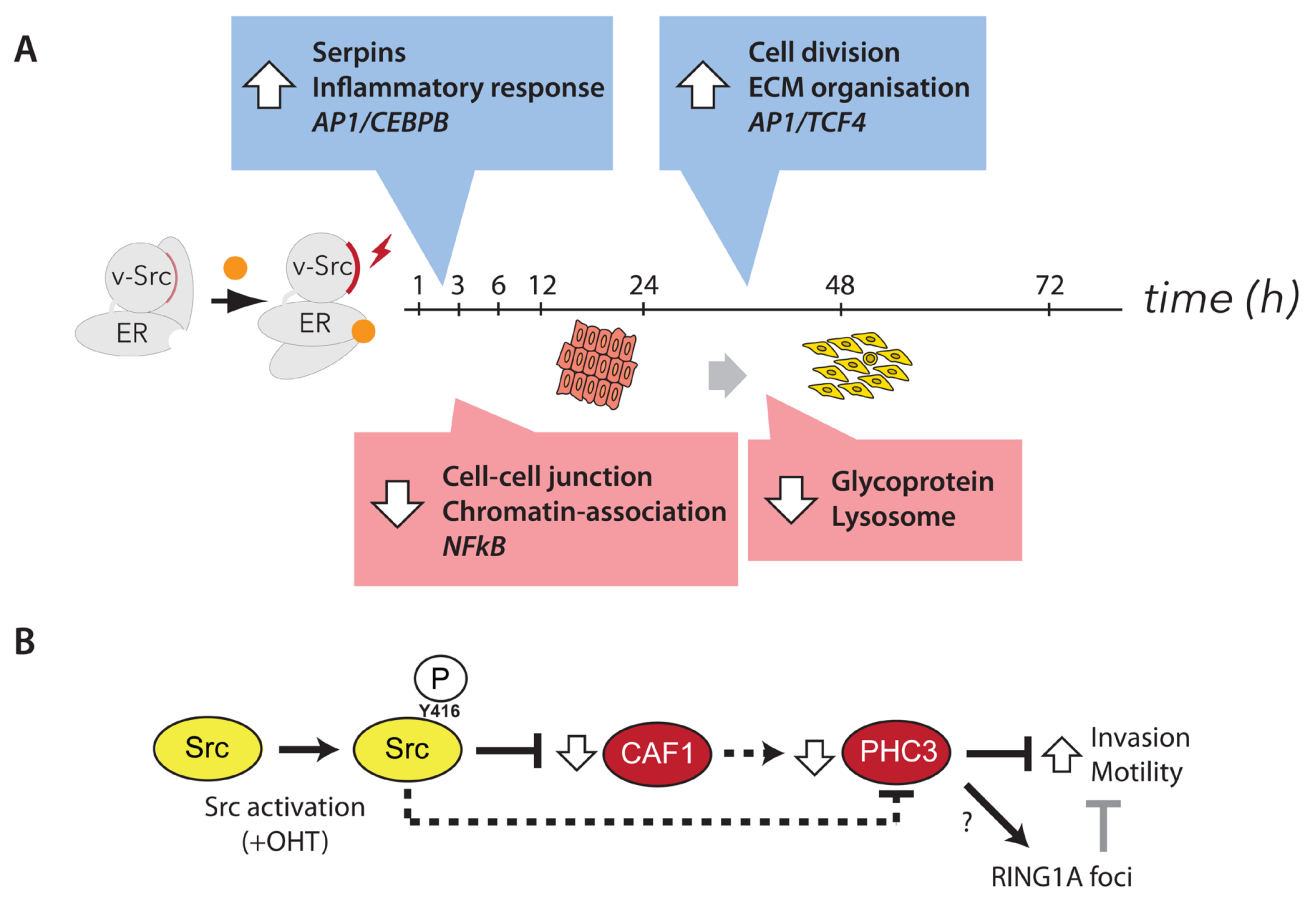
Summary model showing effects of Src activation on the cellular proteome. **A**) Model illustrating the time-course of protein changes observed following v-Src activation. Captions show enriched GO terms from clusters displaying differential response kinetics. **B**) A model summarising the role of CAF1 and PHC3 in suppressing increased cell invasiveness phenotypes.

A major conclusion from our study is that oncogenic transformation of human epithelial cells results in only a small subset of cell proteins (<3%) changing in abundance. Indeed, this represents the abundance-weighted change of an even smaller fraction of the total protein molecules in the cell (<1.5%), because most of the affected proteins are expressed at low copy number. The majority of proteins responding to Src activation (~75%) decrease their expression after transformation, with notable exceptions. Clustering analysis showed that each of the respective groups of proteins showing altered abundance at different times after activation of Src kinase are associated with different cellular functions, as illustrated in Figure 23.

Amongst the earliest detected responses to Src activation are the decreased expression of proteins involved in cell-cell junction interactions and extracellular exosome components, which is consistent with the observed phenotypic changes in cell motility and loss of contact inhibition. Other early responses to Src activation include changes in the abundance of proteins encoded by targets of the transcription factor NF-kappaB. The sprouty homologue 4 (SPRY4) protein, which is involved in suppressing ERK/MAPK signaling, dependent on insulin receptor and epidermal growth factor, also shows a rapid decrease in abundance. With regard to ERK pathways, it is interesting that we also see evidence of Src activation affecting ERK signalling through upregulation of the expression of protein phosphatases DUSP1 and DUSP 4. We also measured a corresponding decreased stoichiometry of phosphorylation of the DUSP target sites, T185 and Y187, on ERK2.

Other early protein responses following Src kinase activation include a rapid increase in the levels of a specific subset of serpin-family protease inhibitors, along with increased levels of proteins involved in the inflammatory response. The response of the serpins to Src activation is of particular interest, as discussed further below also with respect to the effect of transformation on protein turnover. We detect protein expression in the untransformed epithelial cells of 13 different members of the serpin family, but only a subset of these proteins show an abundance change after Src activation, including SERPINs B1/B3/B4/E1/A1, (cf. Figure 11E). These SERPINs inhibit a range of proteases, including papain-like cysteine proteases (SERPINB3) and chymotrypsin-like serine proteases (SERPINB4) ( Schick *et al.*, 1998) as reviewed in ( Sun *et al.*, 2017).

As noted above, SERPINE1 is already used in the clinic as a cancer biomarker. Furthermore, SERPINB3, which shows the most rapid and dramatic increase in abundance, was identified previously as ‘squamous cell carcinoma antigen’ (SCCA1), because it was found in many sera isolated from patients with squamous cell cancers of the cervix (as reviewed in ( Sun *et al.*, 2017)). Subsequently, however, it was also associated with multiple other types of cancer, of either epithelial, or endodermal origins, e.g. lung cancer, head and neck cancer and hepatocellular carcinoma and it was also reported to associate with poor patient survival after chemotherapy and proposed as a predictive biomarker in advanced non-small-cell lung cancer ( Urquhart *et al.*, 2013). Interestingly, SERPINB3 has also been reported to promote oncogenesis and epithelial-mesenchymal transition via the unfolded protein response and IL6 signalling in mammary epithelial cells ( Sheshadri *et al.*, 2014).

Given the very strong clinical links between increased SERPINB3 expression and many forms of aggressive cancers, our finding here that SERPINB3 protein expression is also strongly upregulated by Src activation strongly supports the clinical relevance of studying this human breast epithelial cell model to identify pathways relevant to cancer phenotypes linked with malignancy and poor patient survival. Considering the increased cell motility, invasiveness and morphology phenotypes induced by v-Src activation ( Iliopoulos *et al.*, 2009) ( Endo *et al.*, 2017), it is interesting that we also observe here that transformation coincides with an increase in extracellular matrix (ECM) components, such as laminin proteins (LAMB3, LAMC2), cell surface receptors that interact with the ECM, such as integrins (ITGB, ITGA5), enzymes that remodel the ECM (PLAU, MMP14) and transcription factors that have been shown to play a role in regulating ECM factors (e.g. DLG5). Furthermore, many of the clustered proteins that change in abundance are associated with multivesicular bodies and extracellular exosomes. Such exosome-associated factors include the previously described subset of SERPINS (i.e. B1/B3/B4/E1/A1), which along with IL1β increase in abundance, while there is a parallel decrease in the abundance of the innate immunity factors C3, DCD & M2BP.

In addition to Src activation inducing changes in proteins affecting the ECM and cell-cell interactions, we also see abundance changes in proteins that regulate cell division. For example, there is a rapid decrease in the levels of protein phosphatase 1D (PPM1D), which has a role in inactivation of the checkpoint regulators p53 and Chk1. We also detect an increase in the protein phosphatase CDC25, which is a regulator of the key cyclin-dependent kinase CDK1. Later in the time course (i.e. ‘mid up’ cluster), we detect an increase in the levels of multiple proteins involved in cell division, including CCNB1, AURKB, INCENP and Borealin (CDCA8).

Consistent with cell transformation resulting in specific changes in intracellular signalling pathways, following Src activation we detect abundance changes affecting a small subset of kinases. For example, of the ~330 kinases expressed in the untransformed cells, fewer than 25 alter abundance, including HER-family, Ephrin receptor, Aurora and also Src kinases (cf. Figure 14). Thus, Src, HER3, EphA2, CLK1, Nek6 and AurA kinases all increase, while levels of HER2, CDK4, CHK1, EphB3, EphB4 and TGF-βR2 kinases decrease. It will be interesting in future to link the observed changes in kinase and phosphatase levels with more detailed phospho-peptide quantitation to determine how these responses affect kinase activation and phosphorylation of key protein targets that may influence cell behaviour and contribute to the transformed cell phenotypes. Some of these effects may already be seen with the current data set via analysis of the Reactome pathways available using the EPD database ( www.peptracker.com/epd).

Even although the signature of protein abundance changes we detect as responding to Src activation is linked with poor patient survival, it is likely that the true prognostic value of the proteomic Src signature is underestimated, because not all of these proteins are regulated at the transcriptional level (cf. Figure 20). This is relevant because most of the data in the public domain linking patient survival with gene expression changes use either transcriptome, or microarray assays, to measure mRNA levels and do not directly measure protein expression. Therefore, any cancer-linked changes in protein abundance in patients that arise through mechanisms that do not alter mRNA levels will not be detected.

### Polycomb repressor complex 1 (PRC1)

In considering novel, Src-modulated protein targets that are regulated at the post-transcriptional level, we show here that this includes the Polycomb Repressive Complex 1 (PRC1) subunit PHC3. We have integrated our data from the experiments on PHC3 in this study, with our previous observations that Src downregulates CAF1 ( Endo *et al.*, 2017) and summarized the results in a simplified model ( Figure 22B). We find that either activation of Src kinase activity, or depletion of CAF1 without activating Src, both result in decreased PHC3 protein levels. The data suggest a potential functional role for PHC3, acting downstream of CAF1, in regulating the increases in motility and invasion phenotypes seen in transformed cells. The model therefore highlights that normal expression levels of wild type PHC3 protein are important to suppress cellular motility and invasion phenotypes and that the PHC3 SAM domain is required both for this suppressive function and for localization of PHC3 and RING1A proteins in punctate nuclear foci ( Figure 22A). It will be interesting in future to identify whether any of the genes that are transcriptionally regulated in response to Src activation colocalise at these sites of PRC1 foci.

Our finding here of a potential functional role for the PRC1 complex in cancer cell transformation, and the alteration of phenotypes associated with poor patient survival, is interesting in light of the physiological role of the PRC1 complex in regulating gene expression during embryonic development. The establishment and maintenance of terminally differentiated cell types requires suppression of a range of cell functions that were active in the embryo and that contributed to the programmed cell migrations and interactions required to create tissues and shape the adult organism. Our data are therefore consistent with models in which forms of cancer could result from oncogenes re-activating and/or distorting gene regulators that control cell movement and division in embryos, inducing phenotypes which normally would be suppressed in healthy differentiated cells.

In this regard it is interesting that we find downregulation of PRC1 components occurs downstream of the chromatin associated factor 1 (CAF1) complex, which is also downregulated by Src (illustrated in Figure 23). We previously showed that decreased levels of the CAF1 p150 subunit, in the absence of Src activation, can stimulate cell motility and invasiveness in human epithelial cells ( Endo *et al.*, 2017). In mice, it was shown that the generation of induced pluripotent stem cells (IPSCs), effectively a de-differentiation process, was more efficient when CAF1 subunits were depleted ( Cheloufi *et al.*, 2015). These authors proposed that CAF1 may play an important role in maintaining specific differentiated cell types by regulating the transition state barrier between undifferentiated and differentiated cell states. Therefore, regulation of CAF1 levels by Src, which in turn affects PRC1 expression levels, further supports the idea of cancer phenotypes resulting from a form of ectopic de-differentiation.

PHC3 is a co-factor of the canonical PRC1 complex (cPRC1). All PRC1 complexes contain the core PRC1 components that confer E3 ligase activity, comprising one PCGF protein (e.g. PCGF2/4) and one RING protein (RING1A or RING1B). In addition to the core PRC1 components, cPRC1 complexes also contain one chromobox protein (CBX2, CBX4 and CBX6-CBX8) and one polyhomeotic (Ph) protein (PHC1-PHC3). Additional, non-canonical PRC1 complexes (ncPRC1) have also been described, which contain, for example, RYBP/YAF2. A second set of developmentally and functionally related complexes, the PRC2 complexes have core components that are distinct from PRC1 complexes and have lysine methyltransferase activities (as reviewed in ( Schuettengruber *et al.*, 2017)). The components of PRC1 and PRC2 complexes are illustrated in Figure 15A.

The molecular and functional roles of cPRC1, ncPRC1 and PRC2 complexes are still under investigation. For example, recent work in the fly system, ( *Drosophila melanogaster*) ( Loubiere *et al.*, 2016) has suggested that cPRC1 may have tumour suppressive activity by silencing target genes associated with regulation of cell proliferation, signalling and polarity. They also show in this study that in human Embryonic Stem Cells (hESCs), cPRC1 targeting to these genes is altered in a differentiation-dependent manner. In particular, cPRC1 colocalises with H3K27me3 in hESCs, which then are ‘redeployed’ to genes associated with proliferation, signalling and polarity.

In the original model ( Wang *et al.*, 2004), cPRC1 complexes bind to H3K27me3 marks associated with transcriptional silencing, which are deposited by PRC2. Bound cPRC1 complexes then deposit H2AK119ub marks, which function to transcriptionally silence developmentally regulated genes. However, more recent evidence indicates that the majority of the H2AK119ub marks are mediated by ncPRC1, ( Loubiere *et al.*, 2016) which has contributed to an alternative model (as reviewed in ( Schuettengruber *et al.*, 2017)) where ncPRC1 recruitment to genomic loci via a PRC2-independent mechanism leads to H2A119 ubiquitination, which may then facilitate subsequent binding of PRC2. Our data are consistent with this idea that the majority of H2AK119ub marks are mediated by ncPRC1 complexes. It will be interesting in future to characterise in more detail the different classes of PRC1 complexes that are expressed in differentiated cells and how they are affected by, and contribute to, mechanisms of cell transformation.

### Protein turnover and cell transformation

We, and others, have previously used different pulse-SILAC strategies to measure global protein turnover values for mouse and human cell lines ( Schwanhausser *et al.*, 2011) ( Jovanovic *et al.*, 2015) ( Boisvert *et al.*, 2012) ( Zecha *et al.*, 2018) ( McShane *et al.*, 2016). However, a unique feature of this study is the detailed information it provides measuring protein synthesis, degradation/secretion and turnover rates in both healthy and in transformed cells. To the best of our knowledge, this is the first study to assess the impact of oncogenic transformation on protein half-lives at a systems level. Further, the experimental design chosen allows the most direct comparison of the consequence of transformation because we directly compare protein synthesis and turnover rates in the same epithelial cells, grown either with, or without, activation of an oncogene (i.e. v-Src). Thus, we used our previously described pulse-SILAC method ( Boisvert *et al.*, 2012) to compare protein turnover in the same cells, but under conditions where they exhibited altered phenotypes, following growth -/+ 4-OHT treatment for 48 hrs. This 48 hr time point was selected as the time post v-Src activation where we observed a peak in the protein abundance changes.

All of these turnover experiments were performed in biological triplicate, using cells grown on different days, with the resulting turnover values calculated separately for each peptide identified, as well as for every protein. In total, turnover data were measured for >9,000 separate epithelial cell proteins, including 8,412 proteins where we could determine values in both the untransformed and transformed cells. Open access to interactively explore all of these protein turnover data is provided via the EPD ( www.peptracker.com/epd).

Across the epithelial proteome, protein half-lives showed a log-normal distribution, with a median value of ~11.6 hrs in control, untransformed cells, and a reduced median half-life of ~10.9 hrs in the transformed cells. However, these values are calculated on a per protein basis, which does not take into account the large differences in copy number between proteins with high and low expression levels. To address this, we introduced here the concept of ‘abundance-weighted’ turnover and used this to calculate proteome-level turnover values for the cells under each growth condition. The effect of abundance-weighting is significant, causing the median half-life values for control and transformed cells to increase to 14.2 hrs and 12.7 hrs, respectively.

Our analysis shows that oncogenic transformation resulted in a decrease in the average time taken for the entire epithelial proteome to turn over. Further, the abundance weighted average half-life values determined represent a direct experimental measurement of the time taken for >97% of the total protein molecules in the cell to turn over. We note that when studying global protein turnover, analysing abundance weighted values provides a more comprehensive view of cell proteome turnover and the effect of cell transformation, at a system-wide level, than comparing the unweighted average turnover values. This arises because of the difficulty in practice of accurately measuring half-lives for the many low abundance proteins in the cell. For example, the corollary was that turnover values for ~38% of proteins in the basal proteome were not determined, mostly corresponding to the proteins with lower expression levels. There is therefore a greater uncertainty associated with the value of the unweighted average protein half-life, as compared with the abundance weighted average used here.

Examination of the protein turnover values across the proteome reveal that only proteins with more extreme turnover values show clustering for specific functions and/or activities. Thus, GO term enrichment analysis showed little or no evidence of specific functions associated with the bulk of proteins having half-life values centred around the proteome median value. However, there was clear enrichment of distinct functional classes of proteins with either higher, or lower, than average half-life values.

The proteins showing fast turnover in untransformed cells were enriched for factors involved in cell division. Interestingly, many of the other proteins that show fast turnover include secreted proteins and proteins with a secretion signal peptide and laminin proteins. Since our experiments specifically measured *intracellular* protein turnover, these findings indicate that, at least for these epithelial cells, protein secretion is an important contributing mechanism for many proteins with high turnover rates.

Many of the ECM factors identified have short half-lives and have been shown to be secreted. For example, the secreted enzyme plasminogen activator (PLAU) had a t _1/2_ of 0.6 hr. Structural components of the ECM, such as laminins (LAMA2, LAMA3, LAMA5, LAMB1, LAMB3, LAMC1, LAMC2) and fibronectin (FN1), had a mean t _1/2_ of 2.7 hr, likely resulting from short-lived intracellular residence prior to their secretion. Short half-lives were also seen for many receptors and may reflect ligand binding-mediated receptor recycling. For example, insulin receptor (INSR), had a relatively short half-life, t _1/2_ = 2.7 hr, likely due to rapid recycling of the receptor in the presence of insulin in the cell culture medium ( Okabayashi *et al.*, 1989). Several other receptors also showed short half-lives (< 5 hr), including the IL-6 receptor and the TGFbeta1 and TGFbeta2 receptors; however, it is unclear in these cases whether the short half-life was triggered by ligand binding.

Rapid protein turnover may be contributing to the mechanisms affecting the observed contact inhibition and low cell division phenotypes under the culture conditions used with the untransformed cells during the SILAC pulse. It is likely that the factors associated with mitotic cell cycle and DNA replication show short half-lives because they are actively targeted for degradation during cellular quiescence and G1 phase. Consistent with this idea, previous analyses of protein half-life, which were performed on asynchronous cells that are predominantly in G1 phase, showed short-lived proteins being enriched in ‘cell cycle’ annotations ( Boisvert *et al.*, 2012). Short-lived proteins show an enrichment in Notch signalling, due both to short-lived Notch receptors, NOTCH1 (t _1/2_ = 2.6 hr) and NOTCH3 (t _1/2_ = 3.1 hr), and also downstream factors, many of which regulate the G0/G1 transition, including CCND1 (t _1/2_ = 0.5 hr) and p27 (t _1/2_ = 2.5 hr). Our data are consistent with an important role for targeted protein degradation in repressing cell cycle progression and maintaining the quiescent state. We note that such regulation of steady state protein abundance by a degradation mechanism allows for relatively fast stabilization of protein levels and a rapid response when the cells need to re-activate growth and division.

We also observe a positive correlation in the turnover values for proteins that are predicted to form common complexes, i.e. proteins associated in complexes are more likely to have similar turnover values to other proteins in the complex than to proteins that they do not interact with (cf. Figure 8). These data support the hypothesis that proteins that associate in the same complex can be co-regulated by mechanisms regulating protein stability, such as targeted degradation of unbound, free subunits ( McShane *et al.*, 2016). This is consistent with our previous observations that pools of proteins in different subcellular compartments can show different turnover rates, particularly subunits of large, multi-protein complexes that assemble and function in different compartments, e.g. ribosomes and RNA polymerase II ( Lam *et al.*, 2007) ( Boisvert *et al.*, 2012) ( Boulon *et al.*, 2010).

It is notable that, following cell transformation induced by Src activation, there is a global change in the overall rate of proteome turnover, but only a small number of proteins (<3%), show major alterations in their half-life. The majority of these proteins (>75%), show faster turnover after transformation, with on average a reduction in their half-life of ~8.3 hrs. Interestingly, however, this increased rate of turnover is not always matched by a resulting decrease in protein abundance. Most dramatically, in the case of proteins such as SERPINB3, the exact opposite is observed, i.e., transformation simultaneously results in the protein increasing in intracellular abundance, while also turning over more rapidly.

The parallel destabilization and protein abundance increase of SERPINB3, which we could independently validate using biochemical assays, as well as pulse-SILAC MS, is consistent with our general finding that overall protein abundance and half-life changes are not well correlated (r = 0.03), between control and transformed cells (cf. Figure 16F). We conclude that Src-mediated changes in steady state protein abundances are typically not mediated primarily by altering protein turnover rates. We propose instead that targeted protein degradation may be, at least in part, an important homeostatic mechanism for buffering protein expression levels under normal growth conditions in healthy cells. Thus, for proteins whose expression level is linked with the control of cell behaviour, a temporary increase in expression would result in degradation of the excess protein produced to restore normal levels. We hypothesise that this homeostatic mechanism, involving degradation of excess protein production, is either circumvented, or else simply overwhelmed, by the effects of oncogenes. Thus, we propose that, upon cell transformation, SERPINB3 expression levels are induced to increase by v-Src activation. As the normal homeostatic mechanism tries to reduce levels of SERPINB3 back to that of untransformed cells, the rate of SERPINB3 degradation increases, causing the observed increase in turnover rate. However, in this case, despite the increased degradation, the oncogene-driven increase in SERPINB3 expression still leads to a net increase in the total amount of SERPINB3 protein molecules, which in turn contributes to altering the behaviour and phenotype of the cells.

### The epithelial cell proteome

We have provided here a comprehensive analysis of the protein composition of human breast epithelial cells. Characterisation of cell proteomes, including identification of the specific sets of proteins expressed *and their respective abundance levels*, provides an objective and detailed molecular definition of cell identity. Deep proteome analyses of different mammalian cell types commonly show a wide dynamic range of protein expression levels, with bulk protein abundance typically dominated by proteins expressed by a relatively small number of genes. While some of these hyper-abundant proteins, such as histones and ribosomal proteins, are in common between different cell types and perform core cellular functions, other types of proteins can show major variations in abundance between cell types, linked with the specialised role of the cell. For example, granzymes are amongst the most highly expressed proteins specifically in T lymphocytes, reflecting their role in targeted cell killing by activated T cells ( Hukelmann *et al.*, 2016).

In addition to histones and ribosomal proteins, the proteome of untransformed human breast epithelial cells is dominated by abundant cytoplasmic enzymes and cytoskeleton proteins, e.g. GAPDH and tubulins. Notably, S100 calcium-binding proteins also contribute significantly to the bulk protein composition of these epithelial cells, contributing ~4% of the total protein by copy number. The human genome encodes 21 S100 family proteins, of which 13 were expressed in the untransformed epithelial cells, with 9 ranked in the top 100 most abundant proteins (i.e. S100A2, S100A6, S100A8, S100A9, S100A10, S100A11, S00A13, S100A14, S100A16). Interestingly, S100A8, the third most abundant S100 protein in MCF10A cells, was not detected in a recent deep proteome characterisation of HeLa cells ( Bekker-Jensen *et al.*, 2017). We also see differences in the pattern of S100 proteins expressed in human iPS cells (our unpublished observations; cell lines used can be found on HipSci). This indicates that expression of at least some of the S100 family members is cell type specific.

S100 proteins have been reported to be associated with cancer and to have a role in metastatic disease. For example, high levels of two S100 proteins, i.e., S100A10 and S100A16, are associated with non-small cell lung cancer ( Uhlen *et al.*, 2017). In addition, S100A8 and S100A9 are thought to be important for establishing the cellular niche for metastatic colonisation ( Chaffer & Weinberg, 2011). Interestingly, as observed for SERPINB3/B4, both S100A8 and S100A9 proteins increase in abundance upon Src activation, while simultaneously showing decreased half-life. It is surprising how little is known in detail about the functions and physiological roles of this S100 protein family, considering the major contribution they make to the overall protein abundance in many mammalian cell types and their potential role in cancer cell phenotypes.

The epithelial cell proteome shows that the great majority of genes that are expressed contribute only low, to moderate, numbers of protein molecules. Many of the proteins expressed at low levels nonetheless play very important functional roles. An example is provided by the expression profile of the protein kinase family. While we detect expression of ~330 different kinases, only a small subset of these are expressed at medium to higher abundance levels. Many of the high abundance kinases are metabolic enzymes – e.g. PK, PGK, NME. The most abundant protein kinases in epithelial cells are cAMP-dependent kinase, catalytic subunit, MAPKs, Src and casein kinases. However, ~78% of the kinases expressed in epithelial cells fall within the lowest 5% of expressed proteins by copy number. Nonetheless, this large number of very low abundance kinases can also play critical roles and contribute to regulating major cellular processes and responses, e.g. targeting phosphorylation of proteins that are themselves low abundance factors and thereby modulating cell phenotypes.

It is important to bear in mind the consequence of the wide variation in expression levels of different important protein factors. For example, in screening strategies to identify biomarkers for disease and patient stratification for therapy, technologies that do not detect the many lower abundance protein factors are likely to miss important signatures with prognostic value. Our characterisation here of the proteomic Src Signature, which comprises many proteins expressed at low abundance, is consistent with this view.

Our systematic analysis here of the protein-level consequences of oncogene-induced cell transformation begins the process of mapping an atlas of cell transformation, described at the level of a multidimensional proteome ( Larance & Lamond, 2015). We have focussed here on the effect of transformation on the proteomic dimensions of protein abundance and turnover, together with initial studies also on protein phosphorylation levels. It will be important in future to widen this analysis to also measure the effect of cell transformation on other important proteome dimensions, such as protein-protein interactions and subcellular protein localisation, as well as determining in more detail changes in phosphorylation and other protein post translational modifications. All of these measurements can potentially provide new mechanistic information that cannot be derived from the more widely used current genomics and transcriptomics strategies alone.

Multidimensional proteomics therefore can offer important new insights into the molecular mechanisms responsible for altering cell behaviour and causing the cancer phenotypes resulting in poor patient outcomes. We used this proteomics approach here to characterise the consequences of epithelial cell transformation caused by activation of v-Src. Potentially, other cell transformation mechanisms may affect different downstream pathways to those induced by Src. Therefore, it will be very interesting in future to carry out similar analyses also on cell models where transformation is driven by other oncogenes. This can help to identify the key signalling pathways involved in each case and in particular can reveal common effectors downstream of the activated oncogene that mediate cancer cell phenotypes of clinical relevance for diagnosis and therapeutic intervention.

## Materials and Methods

### Cell culture

MCF10A Src-ER cells (a gift from Kevin Struhl, Harvard Medical School) were grown in F12/DMEM (Life Technologies) media supplemented with 5% horse serum (Life Technologies), 10 µg/ml insulin (Sigma), 100 ng/ml cholera toxin (Sigma), 20 ng/ml EGF (Sigma), 0.5 µg/ml hydrocortisone (Sigma), 0.5 µg/ml puromycin (Roche), 100 units/ml penicillin, and 100 µg/ml streptomycin (Life Technologies) at 37 °C in 5% CO _2_. 293T cells were grown in DMEM (Life Technologies) media supplemented with 10% foetal bovine serum (Life Technologies), 100 units/ml penicillin, 100 µg/ml streptomycin, and 2 mM L-glutamine (Life Technologies) at 37°C in 5% CO _2_. For SILAC labelling, Src-ER cells were grown for 7 days in arginine- and lysine-free F12/DMEM media (Thermo Fisher) supplemented with stable isotope-labelled arginine (R0 or R6) and lysine (K0 or K4) (UK gas), dialyzed horse serum (Dundee Cell Products), and the same supplements as normal cell culture. Src-ER cells were isotopically labelled under exponential growth conditions and allowed to become confluent in 6-well plates.

To measure Src-induced proteome changes (see Figure 2A, Exp A), R0K0 labelled cells were treated with vehicle control (1:1000 v/v dilution of 99%+ ethanol, Sigma) and R6K4 labelled cells were treated with 4-hydroxytamoxifen (4-OHT, Sigma) at a final concentration of 1 µM 4-OHT. Cells were then harvested by trypsinisation at the indicated timepoints after 4-OHT treatment (i.e., 1 hr, 3 hrs, 6 hrs, 12 hrs, 24 hrs, 48 hrs and 72 hrs), counted (Countess, Life Technologies), mixed 1:1 by cell number, washed 2x Dulbecco’s PBS (DPBS, Life Technologies) and then cell pellets collected by centrifugation and snap-frozen.

To measure protein half-life, proteome-wide, in cells treated with vehicle control (See Figure 2A, Exp B), R0K0 and R6K4 labelled cells were both treated with 1:10,000 v/v 99%+ ethanol (Sigma). At 48 hrs, a media change was performed. R0K0 cells were refreshed with new R0K0 media containing vehicle control, whereas R6K4 cells were refreshed with R10K8 media containing vehicle control. Cells were then harvested at the indicated timepoints after media change (1 hr, 3 hrs, 6 hrs, 12 hrs, 24 hrs, 48 hrs and 72 hrs) by trypsinisation, counted (Countess, Life Technologies), mixed 1:1 by cell number, washed 2x Dulbecco’s PBS (DPBS, Life Technologies) and then pellets collected by centrifugation and snap-frozen. A parallel experiment where cells were treated with a final concentration of 1 µM 4-OHT (from a 10 mM stock) was performed to measure protein half-life proteome-wide in Src-activated cells.

The experiments described above (Exp A and Exp B) were performed once a week for three weeks, for a total of three replicates. Each replicate consists of different batches of the same cell line, which were cultured and harvested on different days.

### Sample preparation and Liquid chromatography tandem–mass spectrometry (LC-MS/MS)

Cell pellets were lysed in a buffer containing 2% SDS, 10 mM HEPES, pH 7.4, 1 mM EDTA, 1x cOmplete protease inhibitors mini tablet (Roche) and 1x tablet phosStop (Roche), sonicated at 4C using a probe sonifier (Branson, 10% power, 30 s) and then the homogenate passed through a homogenisation filter (Qiashredder, Qiagen). The protein concentration of the filtrate was determined by BCA assay.

An aliquot of the lysate containing 500 µg protein was reduced using 25 mM TCEP (Pierce) and alkylated using 50 mM iodoacetamide (Sigma). The lysate was precipitated using chloroform-methanol. The resulting pellet was resuspended in 8 M urea in digestion buffer (0.1 M Tris, pH 8.0 + 1 mM CaCl _2_). The pellet was then diluted to 4 M urea with digestion buffer, digested with 1:50 w:w LysC (Wako):protein overnight at 37 °C, diluted to 1 M urea with digestion buffer, and digested with 1:50 w:w trypsin (Pierce):protein for 4 hrs at 37 °C. The peptides were then desalted using 500 mg SepPak cartridges (Waters) using a vacuum manifold (Waters). Desalted peptides were then resuspended in 20% acetonitrile / 80% 0.1 M sodium borate buffer, pH 9.3. Peptides were fractionated by hydrophilic strong anion exchange chromatography (hSAX) ( Ritorto *et al.*, 2013) as previously described ( Ly *et al.*, 2014) and 32 fractions collected. These hSAX fractions were desalted using a tC18 96-well plate (Waters) using a positive pressure manifold (Waters). Peptides were then analysed by LC-MS/MS on RSLCnano-Ultimate3000-Q-Exactive Plus instruments (Thermo-Fisher Scientific). Peptides were trapped on a PepMap C18 precolumn (100 Å) connected to a PepMap C18 EasySpray column (2 µm particle size x 75 µm diameter x 50 cm) using 2 hr gradients (2% to 35% over 120 min) with a constant flow of 200 nl/min. A ‘Top15’ data-dependent acquisition method was used, where the top 15 most abundant ions are selected for MS/MS fragmentation by HCD. The mass spectrometry proteomics data have been deposited to the ProteomeXchange Consortium via the PRIDE partner repository with the dataset identifier PXD009270.

### MS data analysis

MS raw files were processed using MaxQuant version 1.5.2.8, which includes the Andromeda search engine ( Cox & Mann, 2008; Cox *et al.*, 2011). MS/MS spectra were searched against the SwissProt reviewed human reference proteome ( UniProt) accessed on April 15, 2017. Raw files for Exp A were subjected to additional database searches for phosphorylation (STY), methyl (KR), dimethyl (KR) and di-gly (K) post translational modifications. Evidence files from the separate searches were combined using an SQL script (see Supplementary File 2) that removed redundant hits and filtered on 1% FDR at the PSM level. No FDR filtering was performed at the peptide and protein levels; however, using the MAYU normalisation ( Reiter *et al.*, 2009), a protein FDR is estimated at ~5–6%.

Intensities measured in the R0K0 and R6K4 channels at the 48 hr timepoint were used to estimate protein copy numbers for steady state basal and transformed conditions, respectively. Protein copy numbers were calculated using the ‘proteome ruler’ ( Wisniewski *et al.*, 2014), which normalises protein intensities based on the total sum of histone intensities.

Protein turnover data were analysed as described in ( Boisvert *et al.*, 2012). Further details can be found in Supplementary File 1, which contains a description of the ratio normalisation, the kinetic parameter fitting and extra modelling to estimate the contributions from amino acid recycling ( Jovanovic *et al.*, 2015). Weighted medians were calculated using copy numbers as weights. In an ordered list of protein half-lives, the weighted median is taken as the half-life value where the cumulative sum of protein copy numbers equals 50% of the protein copy number total.

Statistical tests were performed using scripts in R (v3.3.0) and Perl (v5.8.9). Differential expression analyses in Exp A were performed using t-tests (R v3.3.0) and shrinkage estimators for variance as described by Opgen-Rhein and Strimmer 2007 and as implemented in LIMMA. Tests for differential half-life (Exp B) were performed by calculating z-scores using the distribution of Δt _1/2_, i.e. t _1/2_ (4-OHT) – t _1/2_ (CTRL) and cut-offs were taken at 95% confidence intervals.

To compare half-lives of proteins within the same CORUM complex, half-life variance was compared between the empirically determined ‘target’ dataset and a ‘decoy’ dataset containing randomly selected proteins, grouped into decoy ‘pseudo-complexes’. The ‘decoy’ dataset contains the same number of protein complexes as CORUM and each protein complex has the same number of protein members. The comparison was then repeated 1000 times, each time using a different seed for randomisation.

### Src signature analysis

A gene signature approach was used to calculate a score for each patient sample in the TCGA dataset. The proteomic gene signature for Src, consisting of 248 proteins, was converted into a normalisation array, i.e. a set of numerals of equivalent length to the gene signature, either -1 or +1, reflecting whether the protein was upregulated or downregulated by Src activation in this proteomic dataset. Normalised gene expression data for the 248 genes were then linearly combined according to the normalisation array (i.e. dot product of normalisation array and expression data) to produce a score (‘Src score’) for each patient in the TCGA dataset. Patients were then grouped into highest and lowest 20% quantiles and their survival compared using KM estimators, ratios of median survival (median survival of highest scoring patients / median survival of lowest scoring patients), and log-rank tests. Quantiles were either calculated using the entire TCGA dataset without cancer type discrimination ( Figure 19A and B), or calculated within each cancer type ( Figure 19C), prior to KM analysis. Bootstrapping was performed by repeating the workflow (score calculation, quantile ranking, and KM analysis) on a set of 248 genes selected at random. To estimate the false discovery rate, the bootstrapping procedure was repeated 1000 times and the number of times the median survival ratio for the randomly selected gene set fell below the target set (i.e. the ‘Src signature’) was recorded. The KM analysis was performed on each gene in the Src signature to identify the most prognostic genes ( Figure 20A).

### siRNA transfection

Cells were transfected with siRNAs using Lipofectamine RNAiMax (Life Technology) at 20 nM final siRNA concentration, according to manufacturer’s protocol. Cells were either harvested, or used for further experiments, 72 h after siRNA transfection. Control siRNA sequence is: 5’-CAGUCGCGUUUGCGACUGG-3’ (MWG). siRNAs utilized pools of four different sequences (Thermo Fisher). p150 siRNAs: LU-019938, PHC3 siRNAs: LU-015805.

### Imaging of cell morphology and wound healing assay

Images of cells were taken under light microscopy, either 48 h after 4-OHT treatment, or 72 h after siRNA transfection. For wound healing assay, wounds were created using a p10 micropipette tip in confluent cells 72 h after siRNA transfection. Cells were washed three times with PBS to remove cell debris and media replaced with F12/DMEM media supplemented with 2% horse serum, 10 µg/ml insulin, 100 ng/ml cholera toxin, and 0.5 µg/ml hydrocortisone. Images of cell wounds were taken under light microscopy at 0 and 16 h after wounding. Opened wound sizes were measured by using TScratch software (v1.0).

### qRT-PCR analysis

Total RNA was extracted with the RNeasy kit (Qiagen). Quantification of mRNA was performed with the Light cycler 450 (Roche), using QuantiFast SYBR Green RT-PCR kit (Qiagen) following manufacturer’s protocol. Briefly, the reverse transcription took place at 50 ˌC for 10 min, followed by activation at 95 ˌC for 5 min, and then 45 cycles of a two-step PCR (denaturation for 10 s at 95 ˌC and annealing/extension for 30 s at 60 ˌC). Quantitation was based on the 2 ^-ΔΔCq^ method. Primers used for qPCR - PHC3 forward: 5’-AGCGGGAAAGAGAACGTGAG, PHC3 reverse: 5’-CAGGCAAAGAATGGATGAAGG, GAPDH forward: 5’CGCATCTTCTTTTGCGTCGCCAG, and GAPDH reverse: 5’GGTCAATGAAGGGGTCATTGATGGC.

### Cell invasion assay

Either 48 h after 4-OHT treatment, or 72 h after siRNA treatment/lentiviral expression, cells were detached with Accutase and counted. Sets of 5x 10 ^4^ cells were spread onto the top chamber of BDBioCoat growth factor reduced MATRIGEL invasion chambers (BD). Assays were performed according to manufacturer’s protocol, by using 5% horse serum and 20 ng/ml EGF as chemoattractants. Positive invading cells were stained with Differential Quik Stain kit (Polysciences) and counted from ten independent fields at 20x magnification (Zeiss Axiovert 25).

### Expression constructs and lentivirus transduction

Human cDNA for PHC3 was obtained from Thermo Fisher. The coding sequence of PHC3 was amplified by PCR from cDNA templates and cloned into pcDNA3(+)-HA vector. To generate pLVX-HA and pLVX-HA-PHC3, the corresponding sequences were amplified by PCR, either from constructs described above, or generated by oligonucleotides synthesis and cloned into pLVX-puro vector (Clontech). PHC3 mutants were generated from pLVX-HA-PHC3 by using a standard mutagenesis protocol. For lentivirus production, 293T cells were triple-transfected by calcium phosphate with two plasmids, encoding essential genes for lentivirus (gifts from Ron Hay, University of Dundee) and either pLVX-HA, pLVX-HA-PHC3 or pLVX-HA-mutants. 16 h after transfection, media was replaced. 72 h after transfection, supernatants containing lentiviruses were filtered and concentrated. Lentiviruses were used to transduce cells in the presence of 8 µg/ml polybrene (Millipore).

### siCAF1 proteomic experiments

Cells were detached with enzyme-free cell dissociation buffer (Life Technologies) and counted using an automated image-based cell counter (Countess, Life Technologies). Equal number of cells were mixed and lysed. Whole cell lysates were reduced and alkylated with 50 mM DTT and 55 mM IAA, respectively, followed by methanol/chloroform precipitation. Samples were then processed as described above for hSAX fractionation and LC-MS/MS analysis.

### Immunoblot analysis

Lysates for SDS-PAGE analysis were prepared in lithium dodecylsulfate sample buffer (Life Technologies) and 25 mM TCEP. Samples were heated to 65 °C for 5 min and then loaded onto a NuPage BisTris 4–12% gradient gel (Life Technologies), in either MOPS, or MES buffer. Proteins were electrophoresed and then wet-transferred to nitrocellulose membranes at 35 V for 1.5–2 h. Membranes were then blocked in 5% BSA in immunoblot wash buffer (TBS + 0.1% Tween-20) for 1 h at room temperature. Membranes were then probed with primary antibody overnight at 4°C, washed and then re-probed with IRdye-conjugated secondary antibodies. All antibodies are listed in Table 1.

**Table 1.** Table of primary antibodies used in immunoblots.

Antibody Name	Supplier	Cataloge Number	Clonality	Species
anti-SerpinB3	Iowa DSHB	CPTC-SERPINB3–2	Monoclonal	Mouse
anti-phospho-Src Family (Tyr416)	Cell Signaling Technology	2101	Polyclonal	Rabbit
anti-Histone H3	Cell Signaling Technology	4499	Monoclonal	Rabbit
anti-PHC3	Bethyl Laboratories	A301-570A	Polyclonal	Rabbit
anti-CHAF1A (p150)	Cell Signaling Technology	5480	Monoclonal	Rabbit
anti-H2AK119ub	Cell Signaling Technology	8240	Monoclonal	Rabbit
anti-Rabbit IgG-IRDye 800CW	Licor	925-32213	--	Donkey
anti-Mouse IgG-IRDye 680RD	Licor	925-68072	--	Donkey

### Immunocytochemistry

Cells were fixed with 4% paraformaldehyde in PBS at RT for 10 min, permeabilized with 0.2% Triton X-100 in PBS at RT for 5 min, and incubated with 5% FBS and 0.1% Tween in PBS on ice for 1 h. After blocking, cells were stained with anti-RING1A (Cell Signaling Technology), and anti-HA (Cell Signaling Technology) antibodies at RT for 1 h. After incubation with primary antibodies, cells were stained with either Alexa Fluor 594-conjugated anti-rabbit IgG antibody (Life Technology), or Alexa Fluor 488-conjugated anti-mouse IgG antibody. To stain nuclei, cells were incubated with DAPI (Sigma) at RT for 10 min after incubation with secondary antibody. Images were captured with a DeltaVision Core Restoration microscope (Applied Precision).

### Data availability

Open access for interactive exploration of all of these proteomic data is provided via the Encyclopaedia of Proteome Dynamics database ( www.peptracker.com/epd).

All MS files are freely available via the ProteomeXchange PRIDE repository using accession PXD009270.

Raw immunoblot images, raw fluorescence microscopy images, raw wound healing images, Ct values from RT-PCRs and cell counts from invasion assays are available at Open Science Foundation. Dataset 1: Proteome-wide analysis of protein abundance and turnover remodelling during oncogenic transformation of human breast epithelial cells. http://dx.doi.org/10.17605/OSF.IO/FWMTN ( Ly, 2018)

Available under a CC-By 4.0 Licence

## Supplementary materials

**Supplementary File 1 – Description of pulse labelling SILAC method to measure protein turnover**

**Supplementary File 2. SQL script.**

**Figure Supplement 1 – (Figure 16). PHC3 mutations affecting Src mediated invasion.**

(A, B) Matrigel-invasion assays of cells expressing PHC3 domain mutants. Error bars indicate s.e.m. with n = 3.

**Supplementary Table 1 – TCGA RPPA analysis**

**Supplementary Table 2 – Proteins Identified in MCF10A and their UniProt Annotations**

**Supplementary Table 3 - Copy numbers in basal cells**

**Supplementary Table 4 – Protein half-lives, basal condition (Exp B)**

**Supplementary Table 5 – Proteins that change during timecourse (Exp A)**

**Supplementary Table 6 – Chromatin and Epigenetic Proteins at 72 hr Timepoint**

**Supplementary Table 7 – Protein half-lives, transformed condition (Exp B)**

**Supplementary Table 8 – Src signature**
