# Supplementary material for: Proteome-wide analysis of protein abundance and turnover remodelling during oncogenic transformation of human breast epithelial cells
Source: Wellcome Open Res. 2018 May 2;3:51. [Version 1] doi: 10.12688/wellcomeopenres.14392.1 (PMC5989152; doi:10.12688/wellcomeopenres.14392.1)
Supplement: Supplementary file 1 [file wellcomeopenres-3-15665-s0000.tgz › 1eeb440b-4da4-4d1d-98ed-69c6e760942e.docx]

## Kinetic Modelling

In order to better understand synthesis and degradation of proteins in a cell, we recall a general mathematical model of Boisvert et al. (2012). Briefly, the model assumes that there is a source of amino acids and the pool of degraded waste products. Both of them contain medium and heavy amino acids. The heavy source pool, is assumed to be the inexhaustible medium in which the cells are grown.

Proteins are synthesized from both medium and heavy amino acids from the source and degraded into the degradation pool. Additionally, degraded amino acids can be recycled from the degradation pool to the source. The model equations can be solved numerically and the degradation curve of the medium proteins is approximately exponential with an asymptotic offset, as illustrated in Fig. 1­­­­. The details of the model can be found in the supplementary material to Boisvert at al. (2012).

The disadvantage of this model is the lack of a simple analytical solution, which makes its predictions less easy to interpret. Here we focus on understanding of the offset observed in experimental data. We consider a simplified model, partially based on Jovanovich et al. (2015), which leads to interpretable analytical solutions. The model equations are written for a protein $i$ with the index dropped for clarity. The protein is synthesised at a constant rate, $S$, and degraded at a constant rate, $k$. There is a contamination of the heavy amino acid source pool via recycling. A fraction $\gamma(t)$ of the amino acids in the source pool is M-labelled (Fig. 2). The master equations are:


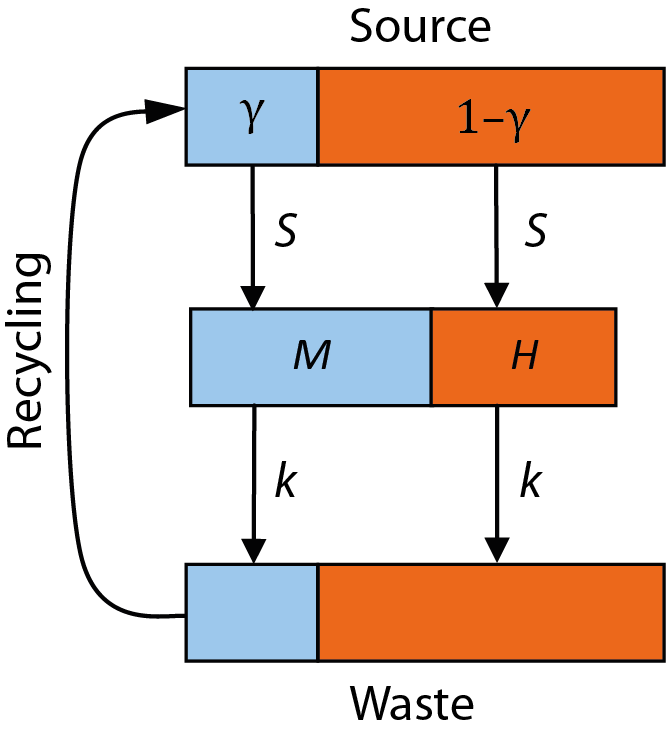


Figure 2. Schematic representation of the synthesis and degradation model.


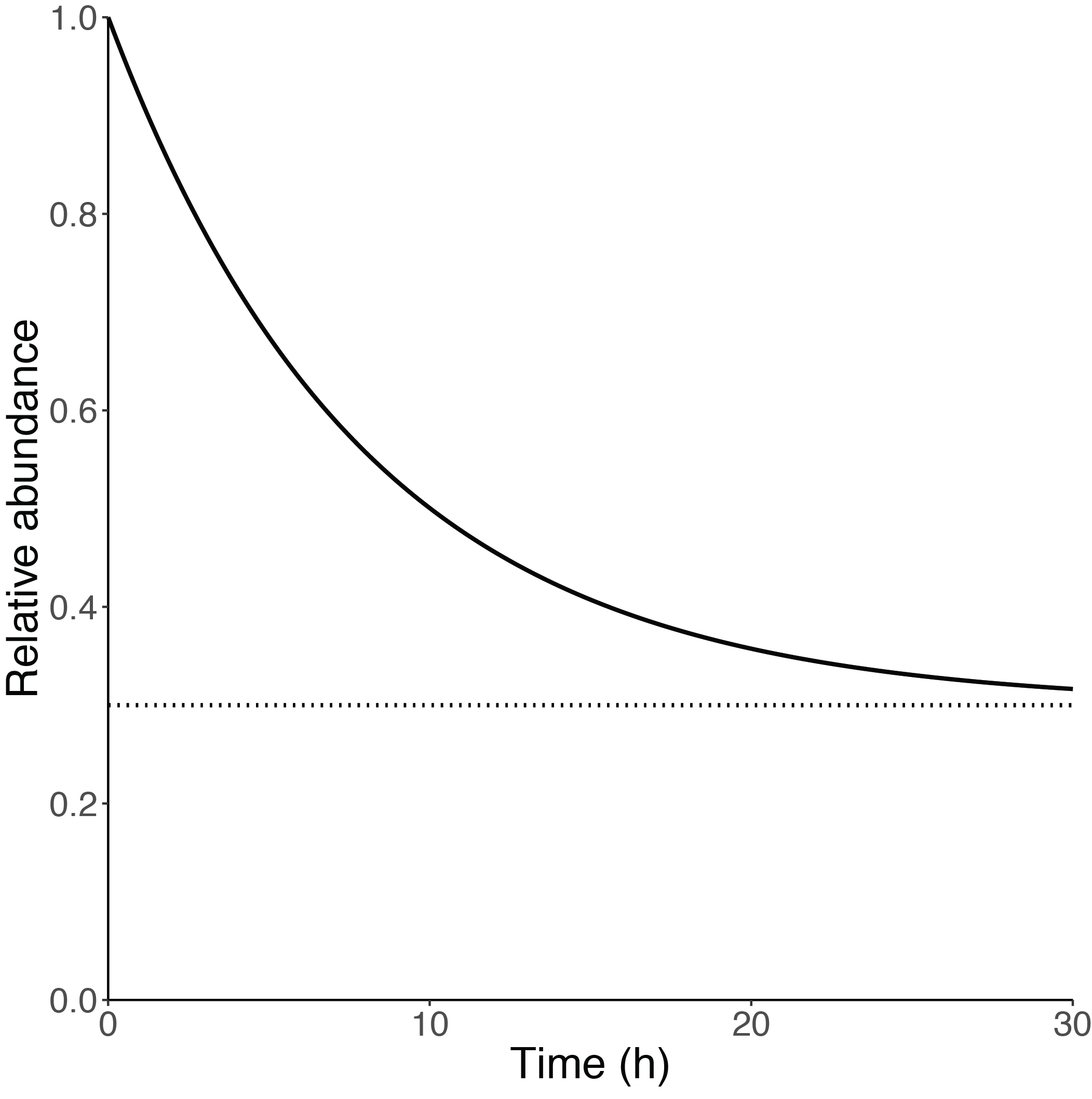


Figure 1. Illustration of the exponential decay with an asymptotic offset, $y\left( t \right)=Ae^{-kt}+\gamma$. The offset, represented by the horizontal dotted line, is $\gamma=0.3$.

| $\frac{dM}{dt}=S\gamma(t)-kM$  $\frac{dH}{dt}=S\left[ 1-\gamma\left( t \right) \right]-kH$ | (1) |
| --- | --- |

where $M=M(t)$ and $H=H(t)$ are dimensionless abundances of medium and heavy proteins, respectively. In experimental data they are represented by L-normalized abundances, that is $M(t)/L(t)$ and $H(t)/L(t)$.

### Constant $\gamma$

In steady state we might expect the contamination fraction to stabilise at a certain level, $\gamma\left( t \right)=\gamma=const$. In such case the equations (1) have a trivial solution:

| $M\left( t \right)=Ae^{-kt}+\frac{S\gamma}{k}$  $H\left( t \right)=Be^{-kt}+\frac{S\left( 1-\gamma\right)}{k}$ | (2) |
| --- | --- |

The initial condition for the bottom equation is $H\left( 0 \right)=0$, hence

| $H\left( t \right)=\frac{S\left( 1-\gamma\right)}{k}(1-e^{-kt})$ | (3) |
| --- | --- |

We require a stationary solution where the total amount of protein is constant, $H(t)+M(t)=const$. Hence,$\frac{d}{dt}\left( H+M \right)=S\gamma-kM+S\left( 1-\gamma\right)-kH=0$ and we find $S=k(H+M)$. If we normalize our data such that $H(t)+M(t)=1$ we find

| $S=k$ | (4) |
| --- | --- |

Synthesis and degradation rates are equal. We can re-write solutions (2) as:

| $M\left( t \right)=Ae^{-kt}$+$\gamma$  $H\left( t \right)=(1-\gamma)(1-e^{-kt}$) | (5) |
| --- | --- |

The degradation curve $M(t)$ follows a simple exponential decay with an offset $\gamma$, as illustrated in Fig. 1. The offset corresponds to the (constant) fraction of contamination of the amino acid source pool (see Fig. 2).

## Asymptotic $\gamma$

The constant $\gamma$ case can be generalized to a scenario where $\gamma(t)$ increases from the initial $\gamma\left( 0 \right)=0$ until the entire system reaches an asymptotic equilibrium state after a while. This seems to be consistent with data where observed $M(t)$ and $H(t)$ reach an equilibrium for many proteins. In the asymptotic state we have

| $\frac{dM}{dt}=0, \frac{dH}{dt}=0$  $\gamma\left( t \right)=\gamma_{\infty}, M\left( t \right)=M_{\infty}, H\left( t \right)=H_{\infty}$ | (6) |
| --- | --- |

Substituting these into the original equations (1) yields

| $0=S\gamma_{\infty}-kM_{\infty}$  $0=S\left( 1-\gamma_{\infty} \right)-kH_{\infty}$ | (7) |
| --- | --- |

Which gives $M_{\infty}+H_{\infty}=S/k$. Again, we require a stationary solution with normalization $M\left( t \right)+H\left( t \right)=1$, hence $S=k$. We reach a similar interpretation of the offset: it is explained by the asymptotic contamination fraction, $M_{\infty}=\gamma_{\infty}$.

## Exponential $\gamma$

One possible functional form for $\gamma(t)$ is an exponential growth, $\gamma\left( t \right)=\gamma_{\infty}(1-e^{-kt})$. In such case the first equation of (1) assumes the form

| $\frac{dM}{dt}=S\gamma_{\infty}(1-e^{-kt})-kM$ | (8) |
| --- | --- |

which can be solved analytically

| $M\left( t \right)=Ae^{-kt}-S\gamma_{\infty}te^{-kt}+\frac{{S\gamma}_{\infty}}{k}$ | (9) |
| --- | --- |

and in a case of balanced synthesis and degradation ($S=k$) we obtain

| $M\left( t \right)=(A-k\gamma_{\infty}t)e^{-kt}+\gamma_{\infty}$ | (10) |
| --- | --- |

This is a modified exponential decay with a constant asymptotic offset, $\gamma_{\infty}$. The shape of this curve resembles a simple exponential decay with an offset. The offset, $\gamma_{\infty}$, is again interpreted as the asymptotic contamination fraction of the source pool.

## Determining gamma

The proportion of contamination in the amino acid pool can be determined by counting double-labelled peptides (that is peptides with one missing cleavage). We follow the derivation of Jovanovich et al. (2015). Let us consider a pool of amino acids where fraction $\gamma$ is M-labelled and $1-\gamma$ is H-labelled. A double-labelled peptide can be produced in four different ways with the following probabilities:

| Labels | Probability |
| --- | --- |
| MM | $\gamma^{2}$ |
| HM | $\gamma(1-\gamma)$ |
| MH | $\gamma(1-\gamma)$ |
| HH | $\left( 1-\gamma\right)^{2}$ |

We cannot distinguish HM from MH, so the probability of observing either of them is $2\gamma(1-\gamma)$. Then, the ratio of HM peptides to HH peptides should be

| $C=\frac{2\gamma\left( 1-\gamma\right)}{\left( 1-\gamma\right)^{2}}=\frac{2\gamma}{1-\gamma}$ | (11) |
| --- | --- |

From which we can find

| $\gamma=\frac{C}{2+C}$ | (12) |
| --- | --- |

Here $C$ is the ratio of abundances of MH-labelled to HH-labelled peptides.
