# Supplementary figures and images for: Proteome-wide analysis of protein abundance and turnover remodelling during oncogenic transformation of human breast epithelial cells
Source: Wellcome Open Res. 2018 May 2;3:51. [Version 1] doi: 10.12688/wellcomeopenres.14392.1 (PMC5989152; doi:10.12688/wellcomeopenres.14392.1)

**A**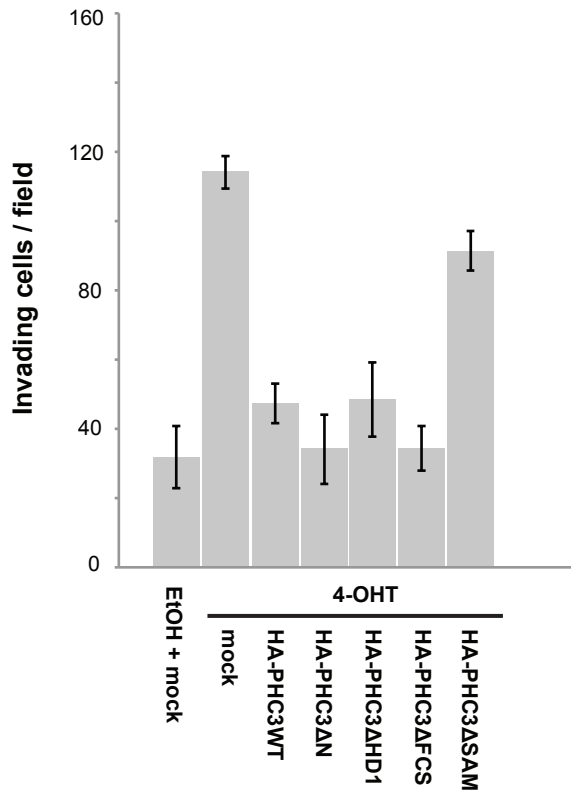**B**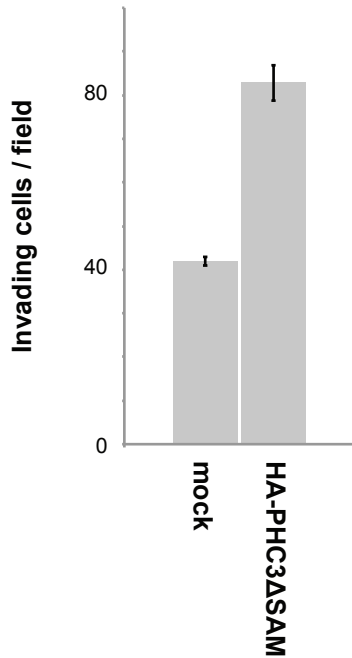

Supplement: Supplementary file 3 [file wellcomeopenres-3-15665-s0002.tgz › 7d94f2e3-3b48-4807-9f46-bed94d7a83cc.pdf]
