# Supplementary material for: Proteome-wide analysis of protein abundance and turnover remodelling during oncogenic transformation of human breast epithelial cells
Source: Wellcome Open Res. 2018 May 2;3:51. [Version 1] doi: 10.12688/wellcomeopenres.14392.1 (PMC5989152; doi:10.12688/wellcomeopenres.14392.1)
Supplement: Peer review history

# Peer review: Referee response for version 1

**DOI:** 10.21956/wellcomeopenres.15665.r33043

**Published:** 2018-06-01

**Authors:** Tony Hunter

**Affiliations:** 1 Salk Institute Cancer Center, Salk Institute for Biological Studies, La Jolla, CA, USA

Here the authors have used deep SILAC-based quantitative proteomic analysis and protein half-life determination to define which proteins change in abundance when MCF10A human breast epithelial cells stably expressing a v-Src-ER HBD fusion protein are treated with 4-OHT to activate the v-Src tyrosine kinase activity for different times (1, 3, 6, 12, 24, 48 and 72 hr). They found that v-Src-induced transformation changed the expression and/or turnover of ~3% of the >12,000 identified proteins in MCF10A cells, which included 330 protein kinases. They also found that v-Src activation increased the average rate of protein turnover of 9,013 measured proteins ~20%, with median protein half-life decreasing from 14.2 to 11.6 hours. A number of v-Src-regulated proteins were found to be present at low abundance and regulated post-transcriptionally. A signature consisting of 248 Src-responsive proteins was found to be predictive of poor patient survival across multiple cancer types when compared to changes in RNA expression level reported in the TCGA database for tumor versus normal. In addition, they showed that protein subunits of the PRC1 chromatin repressor complex, including PHC3 and CBX6, were reduced upon activation of v-Src, and by using siRNA treatment of parental MCF10A cells, they showed that PHC3 depletion was sufficient to elicit increased cell motility and invasiveness.

 These studies represent a huge amount of work, and this dataset and the accompanying analysis provide new insights into the mechanisms underlying malignant transformation of epithelial cells by activated tyrosine kinases, such as v-Src. These results will certainly be useful to the cancer research community, but they would be significantly more valuable if the authors had been able to correlate them with v-Src-induced changes in phosphorylation events and signaling pathways activated downstream of v-Src and also v-Src-induced changes in mRNA levels. Although such additional analyses are beyond the scope of the present paper, it is recommended that the authors carry out such studies in the future, and integrate and correlate the three datasets.

 General points:

1. It would have strengthened their analysis of proteome-wide protein abundance in the treated and untreated MCF10A v-Src-ER cells if they had carried out a parallel RNA-seq analysis to determine whether any of the changes in protein levels were due to changes in RNA levels, and overall how good the correlations between RNA and protein are in this cell system and in response to v-Src activation. For the prognostic analysis, they compared their MCF10A proteome data to an RNA-seq/proteome data set from the human epidermoid carcinoma A431 cell line, but A431 cells and MCF10A cells come from different epithelia and one is also transformed, and therefore it is not clear how useful this comparison is. 
2. The authors indicate that they plan to carry out a parallel phosphoproteomic analysis following v-Src activation, to define direct v-Src substrates as well as all the ensuing downstream Ser/Thr phosphorylation events. Changes in phosphorylation could also be correlated with changes in protein turnover rates, since many proteins are inducibly degraded in response to phosphorylation, which can create phosphodegrons. This will take a significant amount of time, but It would obviously have been ideal if they had been able to combine the proteomic and phosphoproteomic studies..
3. Although it seems likely that most of the observed proteome changes are due to increased v-Src kinase activity, no control was included to rule out the possibility that some of the changes might be due to a noncatalytic function of overexpressed v-Src. While it would be beyond the scope of the present paper to generate an MCF10A cell line that expresses a kinase-dead -v-Src-ER fusion, the authors could use a selective Src inhibitor on the 4OHT-treated cells, e.g. sacrcatinib/AZD0530, and determine whether there are any protein changes observe in response to 4OHT. In this regard, dasatinib will not be useful, because it is a very nonspecific kinase inhibitor, and inhibits many Tyr kinases, including  SFKs, Abl and Arg as well as a number of Ser/Thr kinases. In the same vein, it would be reassuring to have a control in which parental MCF10A cells were treated with 4OHT to ensure that all the observed proteomic changes were due to activation of v-Src-ER.
4. Activation of v-Src not only induces changes in transcription and translation, but also causes dramatic changes in cell morphology leading to transformation, and it is unclear how many of the changes in protein levels observed were due to v-Src induced changes in cell morphology, e.g. cell rounding, partial detachment and disruption of cell-cell contacts, which can induce many changes by altering signaling interactions of integrins, cadherins, etc., independent of any direct effects of v-Src-mediated Tyr phosphorylation. v-Src-induced transformation of epithelial cells is akin to EMT – were proteomic changes noted in proteins known to be causal in this process?
5. Growing MCF10A cells on plastic is a poor recapitulation of how epithelial cells grow in the body, and it would have been better to carry out these experiments with MCF10A cells grown in 3D Matrigel culture, where they form spheroid structures with cell-cell junctions. Moreover, it would be more physiological to grow the cells at 7-8% oxygen, instead of supraphysiological ambient oxygen levels. 
6. The global change in proteome half life in response to v-Src activation is intriguing, but there are no mechanistic insights, i.e. are there changes in the levels of pleiotropic E3 Ub ligases, or changes in global ubiquitylation levels - here a global ubiquitome analysis might be informative.

 

 Other points: 

1. It is not clear what the half-life of a secreted protein means! Apparently, the authors are measuring the kinetics of secretion and not real protein turnover. It seems likely that many of these secreted proteins have long half lives in the ECM, and therefore it is somewhat misleading to say that that they have short half lives, even it this is qualified as an “intracellular” half life.
2. Page 4: It is not clear whether the pY416 Src RPPA signal the authors are referring to specifically measures pY419 c-Src levels or is a combined signal for pY416 Src, pY426 Yes and pY420 Fyn, which share the same autphosphorylation site. In this regard, since the authors have carried out quantitative proteomics analysis, it would be informative to know the relative abundance of the c-Src, Fyn and Yes proteins, and also that of any other SFKs expressed by these cells.
3. Page 9: Proteins present at ≤500 molecules per cell or less, may not represent a truly functional protein population. Depending on RNA and protein half lives, ≤500 protein molecules/cell would probably correspond to fewer than 10 mRNA molecules per cell, and such proteins might be expressed stochastically at the “noise” level. While there may be proteins where only a few hundred per cell are functional (e.g. some surface receptors), for cytoplasmic or even nuclear proteins it has been estimated that a few thousand may be required.
4. It has been reported that c-Myc is overexpressed in MCF10A cells and that Crumbs3, a tight junction protein, is not expressed, which is why MCF10A cells do not form tight junctions ^1^. Did the authors observe high c-Myc and low Crumbs3 protein levels compared to other normal human epithelial cells in their proteomic data?
5. The decreased level of PHC3 induced on v-Src activation and its role in preventing v-Src-induced cell migration is of some interest, but despite the speculation about changes in target gene silencing, there is no evidence that this is in fact the mechanism (would RNA-seq help, or a genome wide methylation analysis?). In this regard, did the authors note changes in the level of proteins involved in EMT, under these conditions.
6. Page 31: The increase in EphA2 RTK levels in response to v-Src activation is interesting, because EphA2 has been implicated as a driver in breast cancer due to its overexpression, but in other cancer types it has been reported to be a tumor suppressor (e.g. Yeddula et al. PNAS 112: E647, 2015).
7. Page 17: Although CDC25C phosphatase activity can be regulated by phosphorylation, since its levels were elevated upon v-Src activation, it would be interesting to know if there a corresponding decrease in pT14/pY15 levels in CDK2 and CDK1 (despite the absence of any alterations in cell cycle phase distribution).
8. Page 3: Eckhart et al. (1979) is not the correct reference for v-Src tyrosine kinase activity - it is Hunter and Sefton, PNAS 77:1311 (1980).

I have read this submission. I believe that I have an appropriate level of expertise to confirm that it is of an acceptable scientific standard.

## References

[1] FoggVCLiuCJMargolisB: Multiple regions of Crumbs3 are required for tight junction formation in MCF10A cells. *J Cell Sci*.2005;118(Pt 13) : 10.1242/jcs.02412 2859-69 10.1242/jcs.02412 15976445

# Peer review: Referee response for version 1

**DOI:** 10.21956/wellcomeopenres.15665.r33045

**Published:** 2018-05-22

**Authors:** Chunaram Choudhary

**Affiliations:** 1 Novo Nordisk Foundation Center for Protein Research, University of Copenhagen, Copenhagen, Denmark

In this work Ly et al. used mass spectrometry-based quantitative proteomics to quantify proteome remodeling during v-Src-induced oncogenic transformation of the human breast epithelial cell line MCF10A. They first characterized the reference proteome of MCF10A cells through in-depth analysis of protein expression as well as by quantifying protein turnover rates in this cell line. Next, they compared the relative changes in proteome expression and turnover rates in control MCF10A cells with v-Src transformed MCF10A cells. These deep proteome analyses, combined with protein turnover rates, allowed the authors to draw several broad, fundamental conclusions, including that v-Src-induced oncogenic transformation requires remodeling of a limited subset of low abundant proteins. Furthermore, the authors show that v-Src significantly increased protein turnover rates in the transformed cells. The oncogene regulated proteins are involved in specific biological processes, such as cell motility and extracellular matrix-interaction. The authors also confirm their previous finding that CAF1 expression is decreased in v-Src transformed cells, and showed that activation of v-Src reduces the expression of PRC1 subunits PHC3 and CBX6. They show that expression of PHC3 is reduced both by activation of v-Src and depletion of CAF1, through a posttranscriptional mechanism. They further show that PHC3 inhibits cell migration; and downregulation of PHC3 is important for v-Src induced cell motility because ectopic expression of PHC3 inhibited v-Src-induced cell migration. By using various deletion mutants, they show that the SAM domain in PHC3 is required for suppressing cell motility. Finally, they show that the “Src signature” proteome, discovered from these systems analyses in MCF10A cell model, is a useful predictive marker for predicting survival of cancer patients. This is despite the fact that the authors used the corresponding mRNA expression profiles for “Src signature” proteins, which likely underestimates the true prognostic value as changes in protein expression is not always accurately reflected in changes in their mRNA expression.

 This tour-de-force study provides a most detailed view of dynamic proteome remodeling in response to oncogenic transformation of human cells, providing several interesting insights in this process. A salient feature of the work is that all the data are made available through a nice web-based resource, which will allow other researchers in convenient mining of the dataset. The dataset will serve as a valuable reference for future studies investigating other aspects of the transformation process, such as v-Src-induced changes in protein phosphorylation or other PTMs.

**Comments:**

 For Fig. 2F, it may be helpful to mention in the text that UniProt keywords include annotation from all cell types in the human proteome. Many of these keywords are related to cell-type-specific processes that are not expected to be covered in the MCF10A proteome. This may help in understanding that 60% coverage of UniProt does not indicate a poor coverage of the MCF10A proteome; rather most of missing terms are likely related to cell-type-specific processes that are not expected to be covered in this cell type.

 Figure 7 should be enlarged so that the text is clearly legible in print.

 Figure 9 (and other figures mentioning fold-change between different conditions), it would be useful to define the conditions that are compared so that it is clearer which conditions are compared. For example: “Log2 fold change protein abundance (4OHT/Ctrl.)”

 For the text related to Fig. 11B, the authors may consider mentioning that v-Src-dependent induction of proteins involved in inflammatory response is consistent with previous work and cite the relevant reference.

 In addition to the global temporal profiles shown in Fig 11, it may be informative to show representative examples of selected proteins from the different clusters (similar to that of Serpins in Fig. 11F).

 The authors show that activation of v-Src reduces protein half-life. It would be useful to show (or at least comment) on the relative proliferation rates of wild-type MCF10A and v-Src transformed MCF10A and whether this has an impact on the calculation of protein half-lives.

 In the text (p 24, second paragraph), the abbreviation KM (Kaplan–Meier) is not defined.

 Figure 19, the text indicating red and green is well-defined and should be explained in the figure legend.

I have read this submission. I believe that I have an appropriate level of expertise to confirm that it is of an acceptable scientific standard.

# Peer review: Referee response for version 1

**DOI:** 10.21956/wellcomeopenres.15665.r33047

**Published:** 2018-05-09

**Authors:** Jeroen Krijgsveld

**Affiliations:** 1 German Cancer Research Center, Heidelberg, Germany

In this study, Ly and colleagues investigate proteome changes that underlie malignant transformation. As a model they use an established MCF10A Src-ER model, where SRC kinase activity can be induced with 4-OHT, initiating a cascade of events leading to cellular transformation within 72h. The authors performed protein expression and protein turnover experiments in a time-course manner to characterise proteome changes during this process. Benefiting from deep proteome coverage, they identified distinct proteome profiles that characterise the different phases of transformation, and provide evidence that protein abundance and turnover are distinct and complementary proteomic entities that do not necessarily correlate. In addition, they identify a ‘Src-signature’ that can also be recognized in gene expression profiles of cancer patients.

 This is a well-designed study containing a wealth of  high-quality data that should be of interest both to the proteomics and cancer biology community. In particular, it contains a number of interesting proteins/observations that call for further functional exploration beyond the ones followed-up on in the manuscript. In addition, it shows some intriguing data where protein turnover and overall expression changes seem to be decoupled (e.g. increased turnover AND expression of Serpin 3/4).

 Remarks:

1. The authors perform deep proteome profiling of MCF10A cells, and then aim to assess ‘how comprehensively our present data set describes the human epithelial cell proteome’. They do so by looking at their data from different angles (sequence coverage, representation of biological functions, etc). Although interesting, it is not hugely insightful, especially since it does not get to the essence in answering the posed question. First, and almost philosophically, it is hard to know how complete the data are without knowing the unknown (i.e. the invisible proteome). Second, beyond ascertaining that most protein classes/functionalities are well-represented in the data, it will be more interesting to explore what biological insights can be gained. For instance, the authors refer to their data as ‘the epithelial cell proteome’, however it remains unclear if/how this contains an ‘epithelial signature’. This is somewhat of a missed opportunity where the authors could have taken advantage of other large proteomics data sets charting proteomes of other (including non-epithelial) cell lines (references in the manuscript). Another suggestion is to compare their data before and after transformation, and observe changes in protein ranking (e.g. S100 proteins?) as a consequence of the loss of epithelial character.
2. Figure 2F is intriguing. First, what is the biological meaning of the fact that a linear fit can be made? After all, it does not intuitively follow (at least for me) that all cellular functionalities should be represented to the same extent, as they may be dictated by the cellular functions that need to be sustained. There will be no cell pushing this to 100%, i.e. expressing proteins that represent all functionalities, so in that sense it is no surprise that the 60% roughly scales with the overall proteome depth. To derive some biological insight from this figure, the authors may look which keywords are off the diagonal, which should reflect functionalities that are under- or over-represented in MCF10A cells.
3. In the original paper describing Src-induced oncogenic transformation using the MCF10A model, Iliopoulos et al (2009) show an inflammatory response as one of the key events, further corroborated in a series of follow-up papers. Do the author see such a response reflected here at the protein level? If not, how may this be explained?
4. Oncogenic transformation is a multi-step process taking several days to complete. The authors do not fully exploit their data to identify and name the order of events happening over time, and possibly connect known phenotypic/morphologic events (e.g. PMID:12132573) to molecular (proteomic) profiles. Figs 7 and 12 are steps in this direction, however the implication for the temporal aspect of malignant transformation is not explicitly made. Looking into this will be of great biological interest, and may lead to an analogous model to step-wise transcriptome/proteome changes as observed during cellular reprogramming to iPS cells (PMID 23260147; 23260666). As an extension, it will be interesting to see discussed how failure to complete these steps may be barriers to transformation, or how this may be pharmacologically targeted.
5. This manuscript is not the first to use MCF10A cells to study malignant transformation. In fact it has been used for profiling isogenic cell lines at various molecular levels, although not necessarily after induction of SRC. Beyond recognizing this related work (e.g. PMID 27512948 and references therein), it will be useful if the authors look at some of these data, with the specific question if the claimed ‘SRC signature’ is really SRC-specific, or rather if it (partly) represents a profile for transformation in general by showing an overlap with signature genes/proteins emerging after Src-independent induction of transformation. If so, this may even expand the scope of the findings presented in this study when comparing this to gene expression profiles of cancer patients.
6. On a similar note, malignant transformation has been studied in other model systems, where the four-stage model based on BJ fibroblasts may be the most relevant, studied at the transcriptome (PMID 23569271) and proteome level (PMID 22313033). Analogous to the point above, comparison with these data may help to refine the ‘Src signature’ or distinguish it from the more general (but equally useful) ‘transformation’ signature.
7. When comparing the proteome data to transcriptome (Fig 20), why did the authors use the Lundberg data? This does not include MCF10A cells, nor does it have a relation to Src. There must be other data (from MCF10A cells) that would better serve the purpose.
8. With regard to the simultaneous increase in turnover AND expression of Serpin 3/4, the authors speculate how this may be achieved mechanistically (essentially assuming that degradation cannot keep up with synthesis), however it remains unclear why this would be the case specifically for Serpinb 3/4, and (thus) if this it serves a function. For instance, and in line with its immediate increase at early time points, the increased turnover/abundance profile may indicate an ‘alarm’ signature reflecting the immediate need for the protein in the face of Src induction, where rapid degradation may ascertain continuous replenishment of the protein pool that may be damaged/modified when executing their function. Since this represents some of the more salient observations in the study, it will be interesting to hear the authors’ view on some of these aspects, with the further notion that SERPINB3 also acts as an inhibitor of JNK1.

I have read this submission. I believe that I have an appropriate level of expertise to confirm that it is of an acceptable scientific standard, however I have significant reservations, as outlined above.
